# New Phosphorus Analogs of Bevirimat: Synthesis, Evaluation of Anti-HIV-1 Activity and Molecular Docking Study

**DOI:** 10.3390/ijms20205209

**Published:** 2019-10-21

**Authors:** Elwira Chrobak, Krzysztof Marciniec, Aleksandra Dąbrowska, Paweł Pęcak, Ewa Bębenek, Monika Kadela-Tomanek, Andrzej Bak, Maria Jastrzębska, Stanisław Boryczka

**Affiliations:** 1 Department of Organic Chemistry, Faculty of Pharmaceutical Sciences in Sosnowiec, Medical University of Silesia in Katowice, 4 Jagiellońska Str., 41-200 Sosnowiec, Poland; kmarciniec@sum.edu.pl (K.M.); pawel.marek.pecak@gmail.com (P.P.); ebebenek@sum.edu.pl (E.B.); mkadela@sum.edu.pl (M.K.-T.); boryczka@sum.edu.pl (S.B.); 2 National Medicines Institute, 30/34 Chełmska Str., 00-725 Warszawa, Poland; aleksandra_dabrowska@o2.pl; 3 Institute of Chemistry, University of Silesia, 9 Szkolna Str., 40-007 Katowice, Poland; andrzej.bak@us.edu.pl; 4 Department of Solid State Physics, Institute of Physics, Silesian Center for Education and Interdisciplinary Research, University of Silesia, 75 Pułku Piechoty 1a, 41-500 Chorzów, Poland; maria.jastrzebska@us.edu.pl

**Keywords:** anti-HIV-1, bevirimat, natural products, phosphate, phosphonate, molecular docking, molecular docking simulations

## Abstract

Since the beginning of the human immunodeficiency virus (HIV) epidemic, many groups of drugs characterized by diverse mechanisms of action have been developed, which can suppress HIV viremia. 3-*O*-(3′,3′-Dimethylsuccinyl) betulinic acid, known as bevirimat (BVM), was the first compound in the class of HIV maturation inhibitors. In the present work, phosphate and phosphonate derivatives of 3-carboxyacylbetulinic acid were synthesized and evaluated for anti-HIV-1 activity. In vitro studies showed that 30-diethylphosphonate analog of BVM (compound **14a**) has comparable effects to BVM (half maximal inhibitory concentrations (IC_50_) equal to 0.02 μM and 0.03 μM, respectively) and is also more selective (selectivity indices: 3450 and 967, respectively). To investigate the possible mechanism of antiviral effect of **14a**, molecular docking was carried out on the C-terminal domain (CTD) of HIV-1 capsid (CA)–spacer peptide 1 (SP1) fragment of Gag protein, designated as CTD-SP1, which was described as a molecular target for maturation inhibitors. Compared with interactions between BVM and the protein, an increased number of strong interactions between ligand **14a** and protein, generated by the phosphonate group, was observed.

## 1. Introduction

Acquired immunodeficiency syndrome (AIDS) is a disease that is still rapidly spreading in many countries and, due to its range, has been called a global epidemic. It is caused by the human immunodeficiency virus (HIV), a member of the retrovirus family, which attacks the cells of the immune system by destroying or impairing their functions. This gradually reduces the host’s ability to fight infections. According to United Nations Program on HIV/AIDS, at the end of 2017, there were 36.9 million people infected with HIV around the world. Intensive research aimed at developing new therapies and implementing prevention programs has reduced viral transmission and the mortality of HIV-1-infected patients. Compared to the number in 2010, the number of the annual global AIDS-related deaths in 2017, which is 940,000, was decreased by 34% [1].

The HIV mutates quickly, resulting in the ineffectiveness of treatment and the development of drug-resistant strains. Therefore, the search for new substances with antiviral activity is still of high importance. Moreover, such new substances should have different mechanisms of action than currently used therapeutics, thus allowing them to be used in a more convenient treatment regimen.

An important method of obtaining new therapeutic agents is the modification of existing leads, that is, substances with known biological activity in terms of optimizing their properties. Such substances include compounds derived from natural sources, like lupane-type pentacyclic triterpene —betulin (lup-20(29)-ene-3*β*,28-diol), as well as its oxidation product—betulinic acid (3*β*-hydroxy-20(29)-lupaene-28-oic acid).

**Figure 1:**
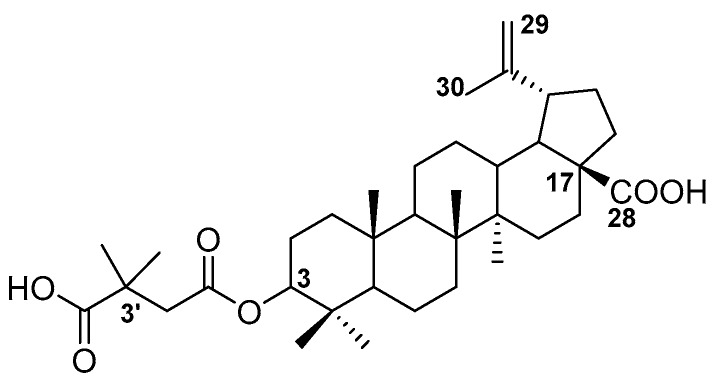
Chemical structure of bevirimat (BVM).

These compounds, as well as their derivatives, have a broad spectrum of biological activity, including anticancer, antiviral, antimalarial, antibacterial, anti-inflammatory and hepatoprotective effects [2,3,4]. However, betulin derivatives inhibiting the HIV-1 replication were described for the first time in 1994 and included betulinic acid (half maximal effective concentration (EC_50_) = 1.6 μM, therapeutic index (TI) = 9.3) and dihydrobetulin (EC_50_ = 0.9 µM, TI = 14) [5]. Betulin, betulinic acid and betulonic acid have weak activity against HIV-1 [6]. However, betulinic acid derivatives represent a promising class of compounds active against the HIV virus. Mayaux et al. described the amide derivatives of betulinic acid designated as RP 70034, RPR 103611 and a derivative of 30-hydroxybetulinic acid, designated as RP 72046, which block the HIV entry into the cell [7]. Studies of primary amides of betulinic acid with a terminal carboxylic group of various alkyl chain lengths, as well as products of their condensation with *α*, *β* or *γ*-amino acids (introduction of an additional side chain amide group), confirmed that the most active compound is the one designated as RPR 103611 and its diastereoisomer IC9564 [8,9]. The greatest hope for a breakthrough in HIV/AIDS therapy was raised by the 3-*O*-(3′,3′-dimethylsuccinyl) betulinic acid (bevirimat, BVM; Figure 1) synthesized by the Kuo-Hsiung Lee team [10].

In relation to HIV-1-infected H9 lymphocytes, an EC_50_ of 0.0035 μM and a TI of 20,000 were determined for this compound [9,10]. The results of further studies have shown that this HIV-1 inhibitor was the first antiretroviral compound defining a new class of drugs with a mechanism of action affecting the virion maturation process and termed maturation inhibitors [11]. The process of replication of HIV-1 ends with virion maturation, which consists of a series of cleavages of virus Gag polyprotein. As a result of this process, a fully processed subpeptides (MA: matrix protein, CA: capsid, SP1: spacer peptide 1, NC: nucleocapsid protein, SP2: spacer peptide 2 and P6: protein) emerge. In the last and slowest step of maturation, HIV protease cleaves a peptide bond between CA and SP1. After that, multiple CA peptides rearrange to create a cone-shaped core, which is critical to the formation of mature and infectious virion [12]. This molecular target is currently used in previously approved antiretrovirals protease inhibitors, such as nelfinavir and indinavir. However, the effectiveness of these drugs is systematically dropping as a result of the emergence of drug-resistant and cross-resistant mutants [13]. Maturation inhibitors use the same mechanism of action, but their molecular targets are distinct. As opposed to inhibitors which affect HIV protease, maturation inhibitors bind to its substrate (CA–SP1 peptide) and prevent the enzyme from cleaving Gag polyprotein. Accumulation of CA–SP1 peptides with the absence of assembled CA proteins leads to loss of infectivity. Interestingly, even mutants resistant to other drug classes remain vulnerable to BVM. BVM has been subjected to clinical testing, which was suspended in phase IIb in 2010, as a result of the emergence of resistance resulting from the mutation of CA–SP1 protein found in about 55% of patients infected with HIV [14]. Nevertheless, BVM has become a prototype of a promising group of maturation inhibitors. Many research groups are working on the synthesis of second-generation inhibitors, also based on the structure of pentacyclic triterpenes, which have improved activity against viruses with SP1 polymorphism [15].

In the search for new active anti-HIV substances that demonstrate high activity and selectivity and reduced toxicity, numerous modifications of the betulin and betulinic acid structure at C3, C28 as well as C30 were performed [16,17,18,19]. However, compounds in which a phosphonate group is directly linked to the triterpene system have not been described so far. 

Compounds containing a phosphorus atom play an increasingly important role in medical chemistry. For example, phosphonates are compounds commonly found in living organisms. They constitute a group of compounds with antiviral and antibiotic activity. Important examples of such antibiotics with broad application in medicine include fosfomycin (a strong effect on Gram-negative bacteria and Gram-positive bacteria), fosmidomycin (antimalarial activity), rhizocticin (antifungal activity) and plumbemycin (antimicrobial activity) [20,21,22,23]. Important antivirals include cidofovir (active against herpes simplex virus (HSV)-1, HSV-2 and Epstein–Barr virus), adefovir and pradefovir (active against hepatitis B virus (HBV)), tenofovir (active against HIV-1, HIV-2 and HBV) and foscarnet (active against herpes viruses and HIV) [24,25,26]. The US patent 2013/0225532 A1 discloses phosphonate compounds active against wild strains of human and bird influenza H1N1, H5N1 and H3N2 viruses resistant to oseltamivir [27]. Moreover, phosphonate derivatives of triterpenes with antiviral activity have been described in several patents. However, the phosphonate group is not linked directly to the triterpene system but is located in the long substituent chain at C3 or C28 positions [28,29,30].

Phosphonates have a P–C bond which, in comparison to the P–O–C bond, has greater stability. As a result, phosphates with a P–O–C bond can be part of a pharmacophore or prodrug element that is cleaved before reaching a molecular target [31]. An example of a phosphate prodrug is fosamprenavir, designed to improve the pharmacokinetics of an oral antiviral drug—amprenavir [32]. To solve the problems of administration and the low bioavailability of triterpenoids, phosphate prodrugs can also be used. For example, phosphate prodrugs of betulin are described in the US Patent 2003/6569842B2 [33].

The subject of this work is the synthesis and evaluation of anti-HIV activity of new compounds, based on the structure of 3-*O*-(3′,3′-dimethylsuccinyl) betulinic acid (BVM), modified by introducing the phosphonate or phosphate substituent directly to the triterpene system. For BVM and for the most active in vitro compounds, molecular docking to the active sites of CA–SP1 protein was conducted. The ligand–target interactions are dependent on the poses (conformation and the relative orientation) of both the host and guest molecule; therefore, molecular dynamics simulations (MDS) were also performed to assess system stability.

## 2. Results and Discussion

### 2.1. Chemistry

**Scheme 1:**
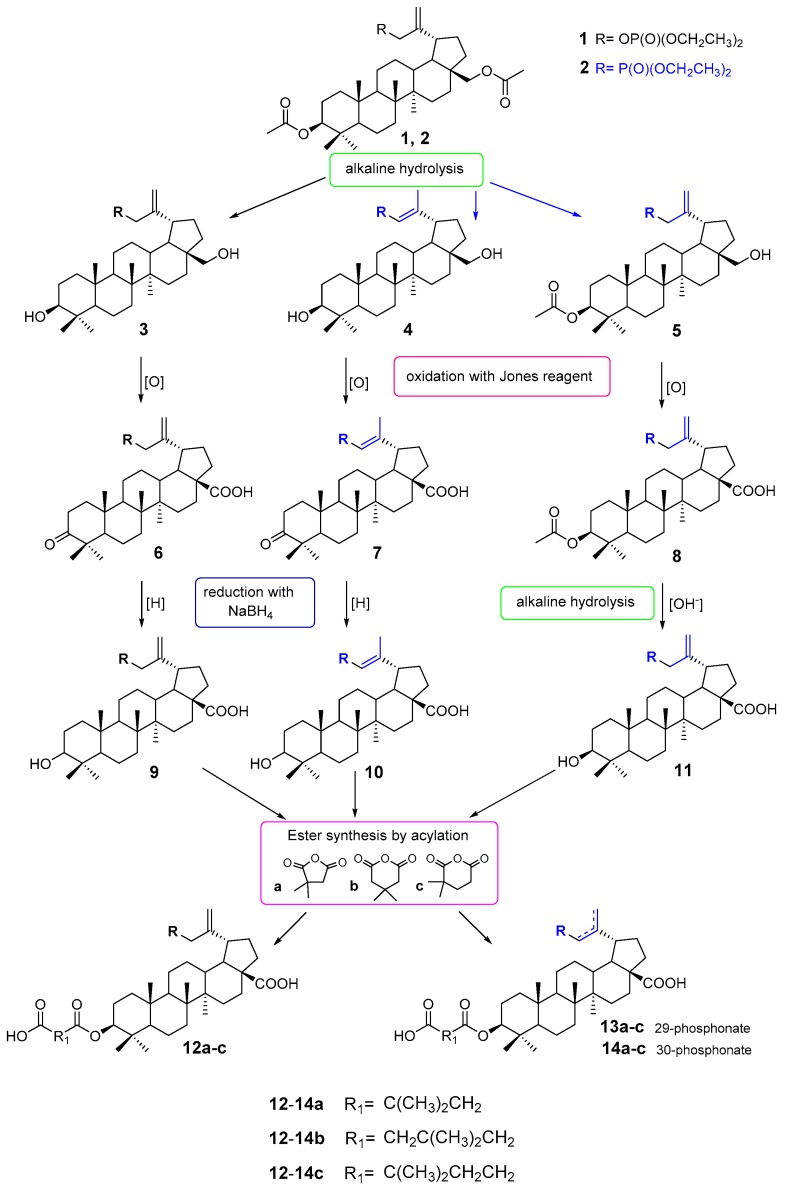
Synthesis of phosphorus derivatives of 3-carboxyacylbetulinic acid **12a**–**c**, **13a**–**c** and **14a**–**c**.

Considering the literature information presented above, it seemed interesting to connect, in one molecule, a residue of a compound with known and reported activity against HIV-1, i.e., 3-carboxyacylbetulinic acid with a phosphate or phosphonate group. This paper is an attempt to answer the question of how this modification of the BVM structure affects the activity of the compounds obtained. For this purpose, three series of phosphorus derivatives were synthesized. These are compounds with the phosphate group in the C30 position (**3**, **6**, **9** and **12a**–**c**), with the phosphonate group in the C29 position (**4**, **7**, **10** and **13a**–**c**) and also with the phosphonate group in the C30 position (**5**, **8**, **11** and **14a**–**c**) (Scheme 1).

The starting substance used in the work was 3,28-diacetylbetulin. This compound was converted into the 30-phosphate **1** and 30-phosphonate derivative **2** according to previously described procedures [34,35]. Betulin phosphate **1** was then subjected to an alkaline hydrolysis reaction to give 30-diethoxyphosphoryloxybetulin **3** with a yield of 85% [34].

As described, the electron-withdrawing effect of the phosphonate moiety in compound **2** increases the acidity of α protons and, under basic conditions, leads to allyl-vinyl isomerization [35]. To hydrolyze the acetyl groups at both the C28 and C3 positions, the reaction should be carried out at room temperature for 6 h. Under such conditions, a mixture of products that are difficult to separate is formed. As previously observed, the hydrolysis reaction of compound **2** carried out in an ethanolic KOH solution at the boiling point proceeds almost entirely with allyl-vinyl isomerization and leads to 29-phosphonobetulin **4** (61%) [35]. Compounds **3** and **4** containing hydroxyl groups at the positions of C3 and C28 were further transformed in the same manner (Scheme 1). First, in the reaction with the Jones reagent, the hydroxyl groups at the C3 and C28 positions were oxidized to the carbonyl group and the carboxyl group, respectively. In this way, 30-phosphate **6** and 29-phosphonate **7** derivatives of betulonic acid (yields of 35% and 31%, respectively) were obtained. The reduction carried out in the next step using sodium borohydride led to the formation of the corresponding betulinic acid derivatives **9** and **10** with yields of 61% and 50%, respectively.

To synthesize the 30-phosphonate derivative of betulinic acid 11, it was necessary to use a different reaction scheme. For this purpose, compound **2** was hydrolyzed under milder conditions (room temperature) and for less time. Such a change in the procedure made it possible to obtain 30-phosphonate of 3-acetylbetulin **5** at a yield of 61% [36]. Then, the hydroxy group at the C28 position was oxidized with the Jones reagent, which resulted in formation of 3-acetyl-30-phosphonobetulinic acid **8** at a 63% yield. This compound was hydrolyzed at the C3 position to give a 30-phosphonate derivative of betulinic acid **11** at a yield of 66%. 

Table 1 shows the different conditions for the alkaline hydrolysis reaction for the formation of phosphonate derivatives **4**, **5** and **11**.

The last step in the synthesis of the target compounds was acylation of the phosphorus derivatives of betulinic acid **9**–**11** with 2,2- and 3,3-dimethyl glutaric and dimethylsuccinic anhydrides. In the literature, several methods of performing this reaction are described. In our work, we adopted a quick and easy method of using a microwave reactor. The reactions were carried out at 130 °C–160 °C to give the corresponding 3-carboxyacyl derivatives **12a**–**c**, **13a**–**c** and **14a**–**c** with yields in the range of 20–41%.

### 2.2. Cytotoxicity and Antiretroviral Activity

The anti-HIV activity of synthesized compounds **9**–**14** were evaluated against HIV-1 in CEM-T4 cell line. The first stage in the study of biological activity was the assessment of cytotoxicity of synthesized compounds using the MTT assay (3-(4,5-dimethylthiazol-2-yl)-2,5-diphenyltetrazolium bromide applied as a reductive coloring agent). The results were expressed as a compound concentration causing 50% cell death (CC_50_). Evaluation of anti-HIV activity consisted of measurements of p24 antigen using the Genscreen ULTRA HIV Ag-Ab Kit. The anti-HIV activity data were expressed as a concentration of tested compound showing 50% inhibition of replication (IC_50_). We included betulin and betulinic acid in our studies in order to broaden the structure-activity study. BVM, the first HIV-1 maturation inhibitor, was applied as a reference compound. The bioassay results are shown in Table 2.

In the study, betulin and betulinic acid did not demonstrate the ability to inhibit HIV replication in the range of concentrations tested. Introduction of a diethylphosphonate substituent in the C29 (compound **10**) or C30 (compound **11**) position of the isopropenyl moiety resulted in an increase in activity as compared to the starting betulinic acid. However, the activity of derivatives **10** and **11** is low, with IC_50_ values above 10 µM. The presence of the diethylphosphate group did not improve the activity of compound **9**. 

As can be seen in Table 2, the presence of a phosphorus atom in the tested betulinic acid derivatives leads to a decrease in the cytotoxic effect relative to the CEM-T4 cell line compared to betulinic acid. This effect is more pronounced for phosphonate derivatives (**10** and **11**) than for the phosphate one (**9**). In addition, the location of the double bond in betulinic acid phosphonate derivatives has little effect on cytotoxicity measured as a cytotoxic concentration CC_50_, that is, as the compound’s concentration required for the reduction of cell viability by 50%. For example, the values for vinyl isomer **10** is 80 ± 1.3 µM and for allyl isomer **11** is higher than 42 µM. Charts of changes in cytotoxicity of all synthesized compounds in the tested concentration range are given in Supplementary Materials (Figures S1–S13). 

When the hydroxyl substituent at the C3 position of the phosphobetulinic acid scaffold was changed to the carboxyacyloxy group, there was an improvement in the activity of the derivatives obtained. Compounds **12c**, **13c** and **14c**, all containing the 4′,4′-dimethylglutaric group, were characterized by the weakest anti-HIV activity and the IC_50_ values determined for them were within the range of 1 to over 10 µM. According to the literature reports, 3′,3′-dimethylglutarylbetulinic acid was characterized by lower activity than BVM (EC_50_ = 0.0023 µM and 0.00035 µM, respectively) [10]. However, triterpene derivatives with 3′,3′-dimethylglutaric moiety such as betulin and 28-aminoanalogs of betulin as well as 28-alkylaminobetulin have demonstrated a very high ability to inhibit HIV replication [37,38]. Sun at al. described 3,28-di(dimethylglutaryl)betulin, which demonstrated strong activity (EC_50_ = 0.00066 µM) and high selectivity (TI = 21515) [37]. Phosphorus derivatives **12b**, **13b** and **14b** with 3′,3′-dimethylglutaric substituent showed significantly lower activity (values of IC_50_ equal to 0.6 ± 0.04, 8.8 ± 1.70 and 0.9 ± 0.12 µM, respectively) (Table 2).

The highest activity in the group of compounds that were tested was found for 3-(3′,3′-dimethylsuccinyl) betulinic acid derivatives **12a**, **13a** and **14a**, for which the IC_50_ values are below 2 µM (Table 2).

**Figure 2:**
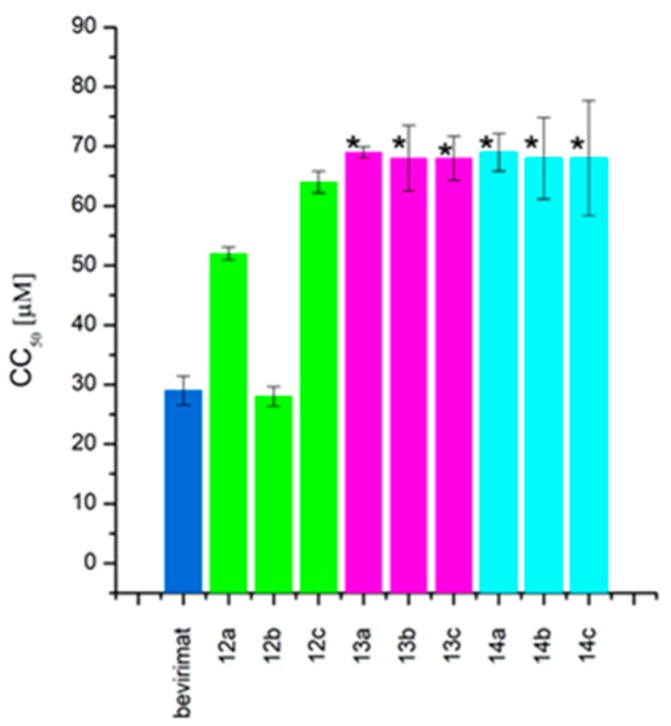
Cytotoxicity (CC_50_) of 3-carboxyacylbetulinic acids divided into groups of corresponding phosphorus derivatives (30-phosphate: **12a**–**c**; 29-phosphonate: **13a**–**c**; 30-phosphonate: **14a**–**c**) and BVM used as a reference. (* the mean measuring error of absorbance at the highest concentration.)

The succinic derivatives **13a** and **14a** have the same cytotoxicity (CC_50_ value of >68 µM, Figure 2) and at the same time show very different activities against HIV-1. The IC_50_ value for the allylic derivative **14a** is 100-fold higher than for the vinylic derivative **13a** (IC_50_ value equal to 0.02 ± 0.01 and 2 ± 0.72 µM, respectively).

**Figure 3:**
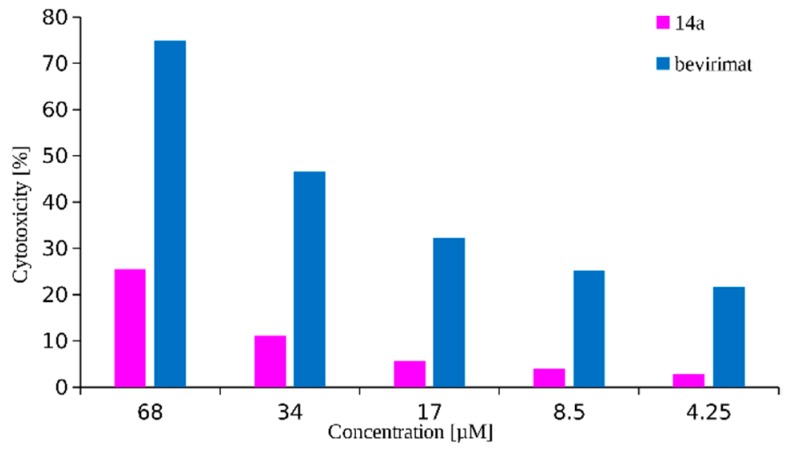
Cytotoxicity of compound **14a** and BVM (reference) in the tested concentration range.

Compound **14a** inhibits viral replication at a level comparable to the reference—BVM (values of IC_50_ equal to 0.02 ± 0.01 and 0.03 ± 0.009 µM, respectively). It should be noted that the advantage of the synthesized derivative **14a** is that it has lower cytotoxicity to the cells tested compared to BVM (Figure 3). The selectivity index for this substance is 3450 and for the reference BVM is equal to 967 (Table 2). 

In the case of derivatives with 3′,3′-dimethylsuccinic and 3′,3′-dimethylglutaric substituents in the C3 position, their activities increase in the order: 29-phosphonate < 30-phosphate < 30-phosphonate.

**Figure 4:**
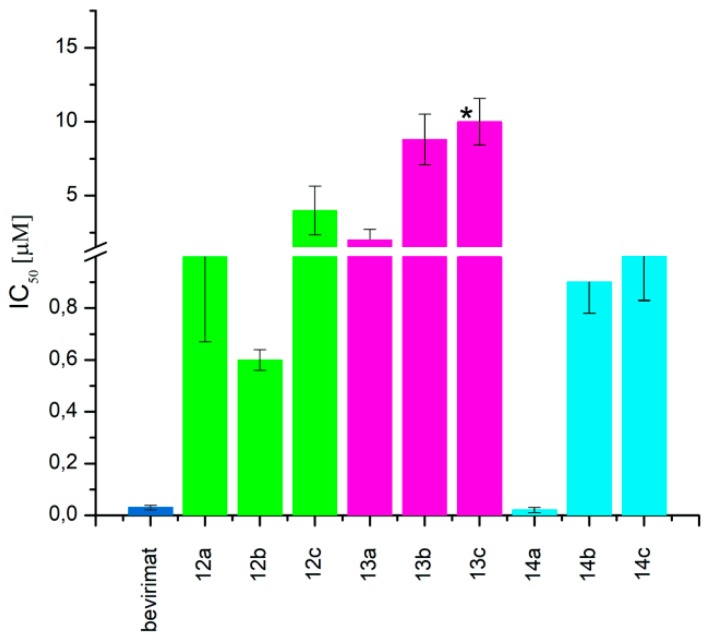
Anti-HIV-1 activity (IC_50_) of 3-carboxyacylbetulinic acids divided into groups of corresponding phosphorus derivatives (30-phosphate: **12a**–**c**; 29-phosphonate: **13a**–**c**; 30-phosphonate: **14a**–**c**) and BVM used as a reference. (* the mean measuring error of absorbance at the highest concentration.)

After analyzing the results, it can be concluded that the introduction of a diethylphosphonate group in the C30 position of the 3-carboxyacylbetulinic acids has increased the ability of the obtained products (**14a**–**c**) to inhibit HIV-1 replication. The corresponding derivatives containing the diethylphosphonate group in the vinyl position (C29), **13a**–**c**, have the lowest activity, regardless of the substitution in the C3 position. Similarly, in the case of 30-phosphate derivatives **12a**–**c**, the presence of an additional oxygen atom does not improve the anti-HIV properties of the compounds obtained (Figure 4). 

Moreover, the higher activity of 3′,3′-dimethylsuccinic (**12a** and **14a**) and 3′,3′-dimethylglutaric (**12b** and **14b**) derivatives as compared to that of 4’,4’-dimethylglutaric (**12c** and **14c**) confirms that an important structural element which determines activity is the presence of two methyl groups on the third carbon atom in the C3 ester substituent. 

### 2.3. Molecular Docking Study

In the group of triterpenoids acting against HIV-1, compounds acting by blocking the virus entry into cells, inhibition of reverse transcriptase and protease, as well as inhibition of virus maturation have been described [4]. Additionally, studies of anti-HIV betulinic acid derivatives showed different mechanisms of their action [39]. Depending on the structure of the substituents at C3 and C28, these compounds act at various stages in the virus life cycle. Derivatives, in which the carboxyl group at the C17 position has been converted to an amide function with various substituents, inhibit the entry of the virus into the cell. Replacement of the hydroxyl group at the C3 position with the amide substituent reduces the activity, whereas the introduction of the ester group gives compounds with high activity and a different mechanism of action, the so-called maturation inhibitors [40]. The derivative **14a**, the most active in vitro synthesized compounds, is a 30-diethylphosphonate analog of BVM, so it can be expected to interact with the same molecular target.

Molecular docking techniques were applied to investigate binding mechanisms for the series of phosphorus derivatives of 3-carboxyacylbetulinic acid (**12a**–**c**, **13a**–**c**, **14a**–**c**) and BVM was selected as a reference compound. The tested compounds can exist as two chemical species: carboxylic acids or carboxylate salts depending on the pH of the aqueous solution. Calculations performed with the ACD/Percepta software [41] for BVM and **14a** show that in both cases the content of the carboxylate states at physiological pH is 100%. Therefore, for docking studies, hydrogens present in the carboxylic groups were removed to mimic the protonation states of these groups.

NMR and cryo-EM data show that in CA–SP1 tubes assembled in vitro, which represent the features of an intermediate assembly state during maturation, the SP1 peptide exists in a dynamic helix–coil equilibrium, and that the addition of the maturation inhibitor, i.e., BVM, causes stabilization of the helical form of SP1. Dynamics of CA and SP1 are critical for orderly HIV-1 maturation and the small molecule can inhibit maturation by perturbing molecular motions [42].

**Figure 5:**
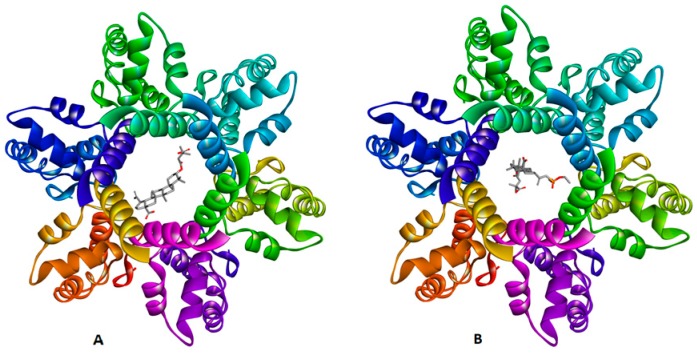
Docking poses of HIV-1 capsid (CA) C-terminal domain (CTD)-spacer peptide 1 (SP1) protein complexes with BVM (**A**) and **14a** (**B**) from a top view.

Based on atomic coordinates of the C-terminal domain (CTD) of CA and SP1, designated as CTD-SP1 fragment of HIV-1 Gag and deposited in the Protein Data Bank (PBD ID 5I4T), one active site cavity was predicted [43]. This cavity was located inside the pore formed by the six-helix SP1 bundle (Figure 5). As a result of docking the computational models of betulin derivatives to the active sites of CTD-SP1, 21 possible ligand–protein complexes were obtained. \All docked compounds bind in the pore region but bind to several different residues through different types of interactions. The superpositions of studied compounds inside CTD-SP1 (poses) were scored according to their binding energies (Table 3).

**Figure 6:**
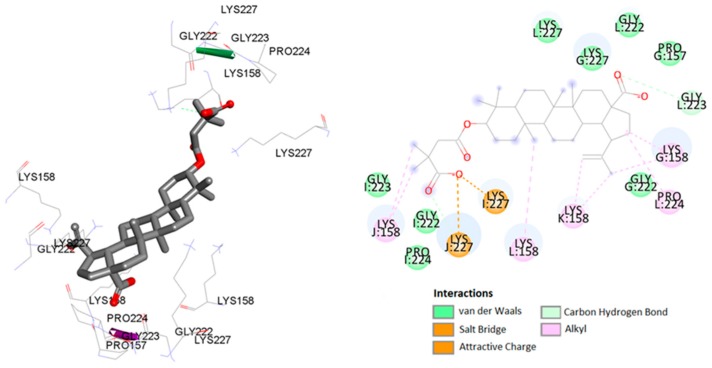
Predicted binding model of BVM with the HIV-1 CA CTD-SP1 protein.

According to the literature data on interaction of BVM with CA CTD-SP1 protein [42], carboxylate moiety of the ligand has ionic interactions with common residue Lys227 in chain I and Lys227 residue in chain L (Figure 6). In addition, numerous hydrophobic interactions, including carbon–hydrogen bonds and carbon–carbon bonds, influence the increased stability of the complex (Table 4).

**Figure 7:**
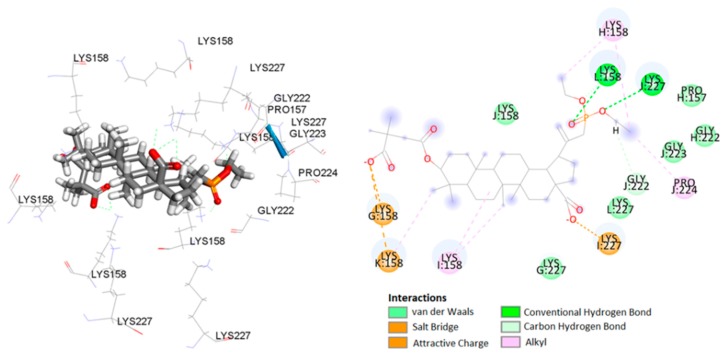
Predicted binding model of **14a** with the HIV-1 CA CTD-SP1 protein.

The analyses of the complex of **14a**, the most in vitro potent compound, included calculations, distance measurements, and pose geometries that determined salt bridge interactions of the ligand pose with Lys227 in chain I and Lys158 residues of chains G and K. Moreover, the phosphonate group forming additional hydrogen–bond interactions of the ligand pose with Lys227 (chain J) and Lys158 (chain L) (Figure 7).

Numerous hydrophobic interactions increase the stability of the complex (Table 4).

**Figure 8:**
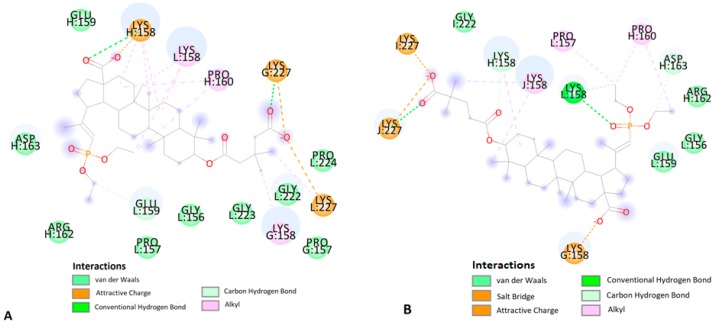
(**A**) Interactions between **13b** and HIV-1 CA CTD-SP1 protein; (**B**) interactions between **13c** and HIV-1 CA CTD-SP1 protein.

The analysis of the docking results presented in Table 3 shows that some of the tested compounds (e.g., compounds **13b** and **13c**) show lower binding energy values compared to compound **14a**. However, detailed analysis of binding modes of **13b** determined hydrogen bonds, formed by carboxylate groups without involving a phosphonate substituent. The analysis of the complex of **13c** determined salt bridge interactions of the carboxylate groups of the ligand pose with Lys227 in chains I and J, and with Lys158 in chain G. It is worth mentioning that, the phosphonate group formed only one, hydrogen–bond interaction of the ligand pose with Lys158 (chain L) (Figure 8). 

The obtained results indicate that the introduction of the diethylphosphonate group into the C30 position of betulin system causes increased stability of the ligand–receptor complex by increasing the number of strong interactions between the ligand and the HIV-1 protein.

### 2.4. Molecular Dynamics Simulations 

**Figure 9:**
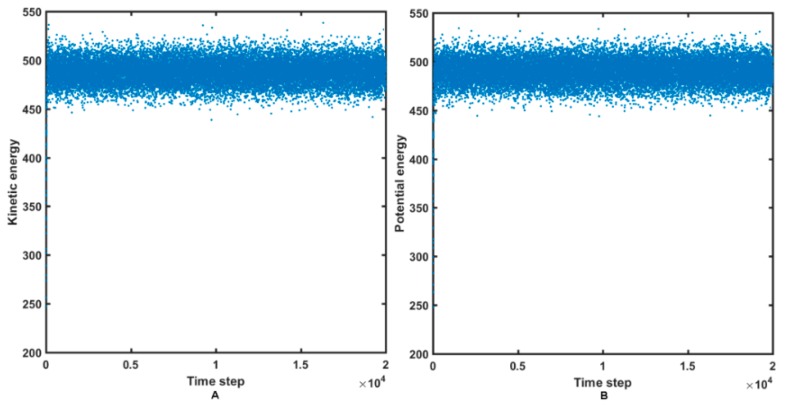
Kinetic (**A**) and potential (**B**) energy variations in the reduced molecular dynamics (RedMD) simulations of HIV-1 CA CTD-SP1 protein.

The molecular dynamic study can provide valuable knowledge about the ligand–target stability and mechanism of the host binding in the active site as well. The liganded states (holo) of the HIV-1 CA CTD-SP1 protein with BVM and the most potent derivative **14a** were used as the initial structures in the MDS. Due to time and computational resource restrains, docking-based MDS of BVM and **14a** molecules were performed with the flexible ligand and the rigid target protocol implemented in Sybyl-X 2.2.1 package. On the other hand, the simulations of the internal motions in a large biomolecular system (protein) were conducted using reduced molecular dynamics (RedMD) strategy, where each amino acid or nucleotide is represented by a single spherical particle (“bead”) centered on an alpha carbon (Cα) or phosphorous (P) atom with mass corresponding to the total mass of a specified amino acid or nucleotide, respectively [44,45,46]. RedMD simulations were carried out in the NVT ensemble using the Berendsen algorithm for the temperature control. Initially, the atom velocities were assigned at 293 K, but then, the system was heated and the conformational investigation was performed at 300 K. The overall length of the RedMD simulations was set to 20 ns. The relatively constant fluctuations of the protein kinetic/potential energy are illustrated in Figure 9A,B.

**Figure 10:**
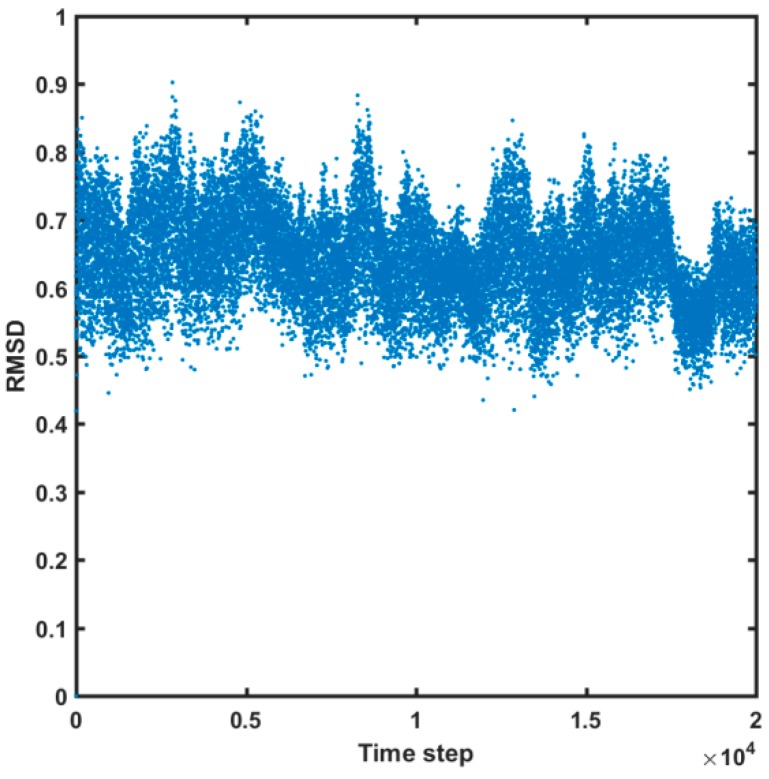
Root-mean-square deviations (RMSDs) of HIV-1 CA CTD-SP1 protein “bead” atoms in RedMD simulations.

Moreover, the potential stability of the HIV-1 protein model can be confirmed by “pretty” small variations of the root-mean-square deviation (RMSD) parameter, as shown in Figure 10.

**Figure 11:**
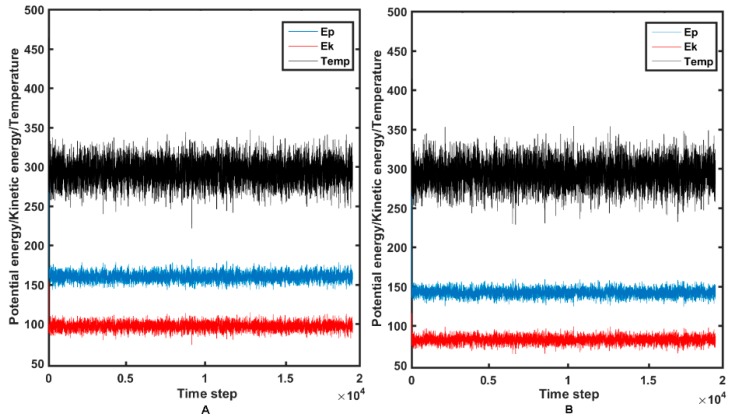
Kinetic, potential energy variations and temperature changes in the molecular dynamics simulations (MDS) of molecule **14a** (**A**) and BVM (**B**).

The set of conformers for BVM as a reference compound and the most active **14a** molecule was generated using the NVT ensemble at a temperature of 300 K. Variations of the potential/kinetic energies as well as the temperature during the 20 ns trajectory for **14a** and the reference BMV molecule are presented in Figure 11A,B, respectively.

**Figure 12:**
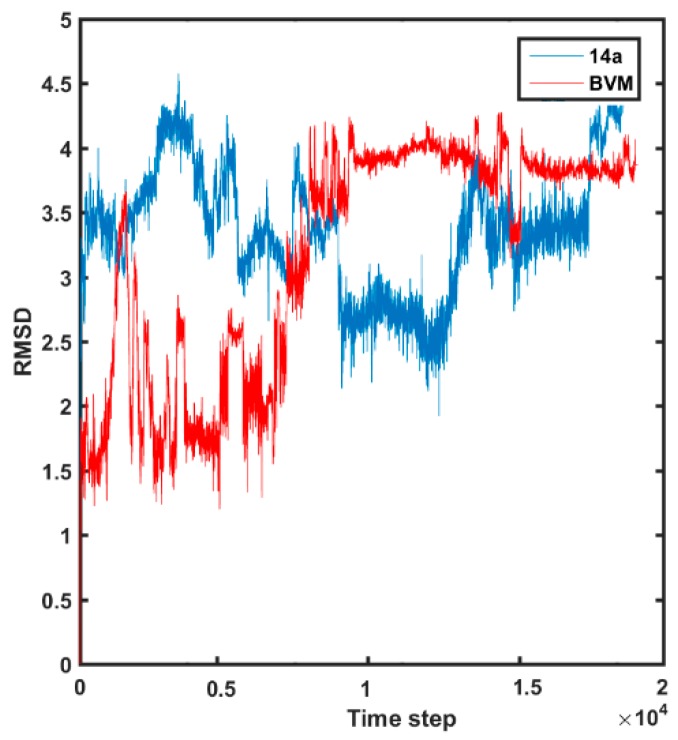
Variations of RMSD values in MDS of molecule **14a** and BVM.

The conformational flexibility of molecule **14a** and BVM can be compared using the RMSD parameter calculated for non-hydrogen atoms, as depicted in Figure 12.

**Figure 13:**
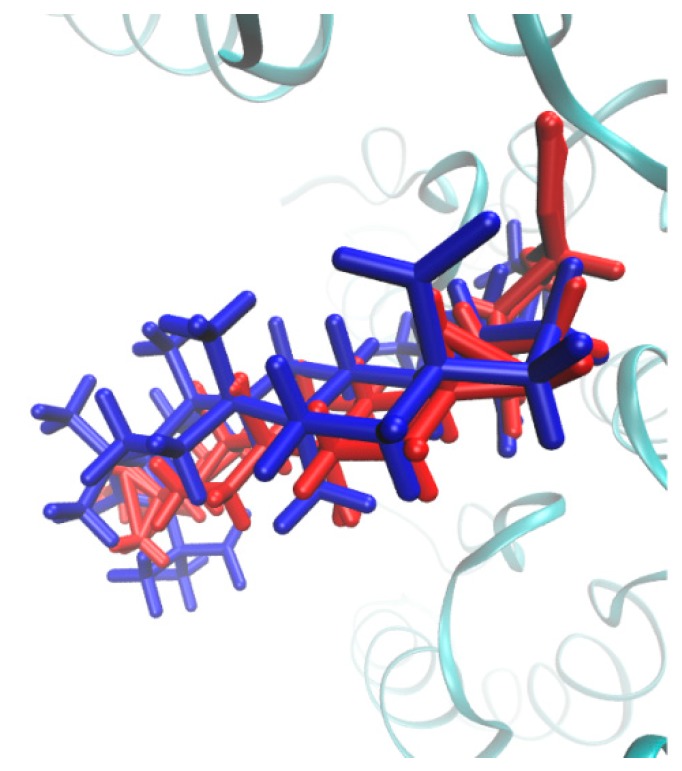
The comparison of the initial (blue) and MD-averaged (red) three-dimensional (3D) geometries of molecule **14a**.

Interestingly, the reference BVM compound is characterized by lower values of RMSD parameters compared to the potent molecule **14a** at the initial steps of the MDS. Obviously, the side chains of compound **14a** have direct impact on the whole molecular flexibility, especially at the beginning of MDS. The comparable RMSD values are reached at the final step of the MDS, which confirms the relative stability of the ligand–protein systems. In Figure 13, the initially docked-based three-dimensional (3D) structure is compared with the averaged spatial geometry of molecule **14a** produced during the MDS.

It seems that the docking protocol generated fairly stable conformation of the most active molecule **14a** in the analyzed series of compounds.

## 3. Materials and Method

### 3.1. Biological Activity

#### 3.1.1. Cytotoxicity

For the determination of compounds cytotoxicity, CEM-T4 cells were obtained from the NIH AIDS Reagent Program (NIH, US) and were cultured in RPMI supplemented with 10% FCS (Biochrom) and antibiotics at 37 °C in 5% CO_2_ on 96-well culture plates. The experiments were carried out in media containing tested compounds in concentrations of 4.25, 8.5, 17, 34 and 68 µM. Cultures in a neat medium (RPMI, 10% FCS) were used as a control. Viability of cells was determined after 7 days using the MTT assay [47] in which 10 μL of MTT solution (5 mg/mL) was added to each culture plate well, and cultures were incubated for 3 h at a temperature of 37 °C. After the centrifugation, the supernatant was removed, and DMSO was added for lysis of the cells and to dissolve crystals of formazan. Color intensity was measured with a plate reader at 560 nm. Cell viability was determined by the Equation (1):

Viability (%) = (OD of samples enriched in test drugs/OD of control samples) × 100(1)

where OD means optical density.

Based on the calculations, a graph was created regarding the relationship between the concentration of the compound and the viability from which CC_50_ was established.

#### 3.1.2. Anti-HIV Activity

CEM-T4 cells were preincubated (culture plates with 96 flat bottom wells) for 24 h under standard conditions (37 °C, 5% CO_2_) and in a standard medium (RPMI, FCS 10%) enriched with tested compounds in the concentration range from 0.02 µM to 10 µM. In each well, 20,000 cells were suspended in the solution of a tested compound (200 μL). For each concentration, cultures were run in triplicate. A wild-type HIV-1 was isolated from the HIV-positive patient in the Laboratory of Virology of the National Medicines Institute (Warsaw, Poland) and was used as a reference. A culture of CEM-T4 lines in a standard neat medium (RPMU, FCS 10%) was used to produce viruses. After 24 h of incubation in a medium enriched with a tested compound, cells were inoculated with a known amount of HIV, and after 7 days, HIV replication was evaluated through the measurement of secreted viral protein p24 carried out with the enzyme-linked immunosorbent assay (ELISA) technique [48]. For each tested compound and for each concentration, the measurements of p24 antigen were done in triplicate using the Genscreen ULTRA HIV Ag-Ab Kit (Biorad, Warszawa, Poland) and following manufacturer’s instructions. The absorbance of the sample was determined as an OD measured at 650 nm. HIV replication capacity was calculated using the Equation (2):

Virus replication (%) = (OD of drug-enriched samples/OD of control samples) × 100(2)

The results were presented by a graph showing the ratio of HIV replication to the concentration of the tested preparation. The concentration of tested compound showing 50% inhibition of replication (IC_50_) was read from the graph.

### 3.2. Synthesis

All solvents used in reactions were dried and purified according to standard procedures. Melting points of obtained compounds were measured in open capillary tubes on an Electrothermal melting point apparatus without correction. 

^1^H NMR (600 MHz), ^13^C NMR (150 MHz) and ^31^P NMR (243 MHz) spectra were recorded on a Bruker AVANCE III HD 600 spectrometer (Bruker, Billerica, MA, USA) in deuterated CDCl_3_. Chemical shifts (δ) values were reported in parts per million (ppm) relative to the external standards. Multiplicity was designated as singlet (s), doublet (d) and multiplet (m). 

Infrared spectra (pellets, KBr, Merck, Darmstadt, Germany) were obtained using an IRAffinity-1 FTIR spectrometer (Shimadzu, Kyoto, Japan) and the measurement was recorded in the range of 4000–1000 cm^–1^ at 295 K. High-resolution mass spectra have been measured with Bruker Impact II (Bruker, Billerica, MA, USA). Calculation of the theoretical molecular mass for compounds was performed using “The Exact Mass Calculator, Single Isotope Version” (http://www.sisweb.com/referenc/tools/exactmass.htm).

The progress of reaction was monitored by thin-layer chromatography (TLC) (silica gel 60 254F plates, Merck, Darmstadt, Germany) and developed in a mixture of chloroform or dichloromethane and ethanol (15:1, *v*/*v*). The spots were visualized by spraying them with a solution of 5% sulfuric acid and heating to 100 °C. The compounds were purified by column chromatography (silica gel 60, particle size < 63 μm, Merck) in the solvent system indicated. 

Acylation reactions were performed in a microwave reactor (Discover SP-D, CEM Corporation, Matthews, NC, USA).

Synthesis of compounds **1**–**5** were performed as was previously described. The melting point and ^1^H, ^13^C, ^31^P NMR spectral data for these compounds were consistent with the literature information [34,35,36,37]. 

BVM [3-*O*-(3′,3′-dimethylsuccinyl) betulinic acid], used in the study as a reference, was obtained from betulin, which was oxidized and the obtained betulonic acid was first reduced and then acylated with dimethylsuccinyl anhydride. The synthesis used the procedures described below for phosphorus derivatives. The final product was obtained at a yield of 70%. The melting point and spectroscopic characteristics of the compound were consistent with the literature data [10]. Characteristics and descriptions of spectroscopic data for newly synthesized compounds are included in Supplementary Materials.

#### 3.2.1. Synthesis of 3-Acetyl-30-Diethoxyphosphorylbetulin 5

The compound **2** (0.66 g, 1 mmol) was added to 50 mL of a 0.2 M solution of sodium hydroxide in a mixture of methanol, tetrahydrofuran and water (1:2:1, *v*/*v*), and stirred at room temperature for 0.5 h. When the reaction was completed, 30 mL of dichloromethane was added, and next, the mixture was washed with a 10% solution of hydrochloric acid and then with water. The organic layer was dried with anhydrous sodium sulphate and evaporated. Purification of the residue by column chromatography (SiO_2_; chloroform/ethanol ratio: 15:1, *v*/*v*) gave product **5**.

#### 3.2.2. General Procedures for Oxidation with the Jones Reagent

A cold solution of the Jones reagent (4 mL) was added in drops to a cooled solution of 1 mmol of compound **3** or **4** in 14 mL of acetone, keeping the mixture temperature at about 0 °C. Stirring was continued for 1.5 h at room temperature. Then, the mixture was placed in a water bath at 10 °C, 3 mL of ethanol was gradually added, and stirring was continued for 30 min. After that, the mixture was poured into 15 mL of water with crushed ice. The suspension formed was extracted with dichloromethane. The extracts were dried with anhydrous sodium sulphate and, after concentrating, were purified by column chromatography (SiO_2_; dichloromethane/ethanol ratio: 15:1, *v*/*v*) to obtain compounds **6** and **7**.

Oxidation of compound **5** was carried out in the same way using 2 mL of the Jones reagent.

#### 3.2.3. General Procedures for the Sodium Borohydride Reduction (Synthesis of Compounds **9** and **10**)

To a suspension of 0.12 g (about 0.2 mmol) of compound 6 or 7 in 8 mL of tetrahydrofuran, 0.03 g (0.72 mmol) of sodium tetrahydroborate (NaBH_4_) was added and stirred at room temperature for 2 h. The reaction mixture was diluted with 15 mL of water and extracted with dichloromethane. Then, the extracts were dried over anhydrous sodium sulfate and concentrated to dryness in a vacuum evaporator. The residue was purified by column chromatography (SiO_2_; dichloromethane/ethanol ratio: 15:1, *v*/*v*) to give the corresponding product (**9** or **10**).

#### 3.2.4. Synthesis of 30-Diethoxyphosphorylbetulinic Acid **11**

Compound **8** (1 mmol) was added to 60 mL of a 0.2 M solution of sodium hydroxide in a mixture of methanol, tetrahydrofuran and water (1:2:1, *v*/*v*/*v*), and stirred at room temperature for 24 h. When the reaction was completed, the volatile constituents were evaporated at a reduced pressure, and 30 mL of dichloromethane was added to the evaporation residue, which was washed with a 10% solution of hydrochloric acid and then with water. The organic layer was dried with anhydrous sodium sulphate. Upon purifying, the crude product by column chromatography (SiO_2_; chloroform/ethanol ratio: 15:1, *v*/*v*), compound **11** was obtained.

#### 3.2.5. General Method of Synthesis 3-Carboxyacyl Derivatives **12a**–**c**, **13a**–**c** and **14a**–**c**

To the solution of 1 mmol of appropriate acid 9, 10 or 11 in 2 mL pyridine, N,N-dimethylaminopyridine (DMAP; 1.5 mmol, 0.19 mg) and 5 mmol of appropriate acid anhydride (2,2-dimethylsuccinic anhydride or 3,3-dimethylglutaric anhydride or 2,2-dimethylglutaric anhydride) was added. The reaction vessel was placed in a microwave reactor and the reaction was carried out for 1.5 h at a temperature of 130°C–160 °C at a maximum wave power (300 W). After cooling, the mixture was diluted with 25 mL of ethyl acetate, and then washed with a 20% hydrochloric acid solution and with water. The organic layer was dried with anhydrous sodium sulphate and concentrated until dry in a vacuum evaporator. The crude product was purified by column chromatography (SiO_2_; dichloromethane/ethanol ratio: 15:1, *v*/*v*) to obtain phosphorus derivatives of 3-carboxyacylbetulinic acid **12a**–**c**, **13a**–**c** and **14a**–**c**.

### 3.3. Molecular Docking Study 

The 3D structures of studied compounds were generated in their low-energy conformation using the Gaussian 16 (revision A.03) computer code [49] with the density functional theory (DFT, B3LYP) and 63–11 + G(d,p) basis sets.

A target macromolecule for molecular docking studies was obtained from the Protein Data Bank (https://www.rcsb.org/). We used 3D hexameric crystal structure of the CTD-SP1 fragment of HIV-1 Gag (PDB ID 5I4T). Incomplete or missing side chains of CTD-SP1 protein were restored with the SWISS-MODEL Workspace (https://swissmodel.expasy.org/) [50]. Geometry optimization of model was done by the optimization protocol in YASARA Energy Minimization Server (http://www.yasara.org/-minimizationserver.htm) [51] and a ligand-binding site in protein was calculated using AutoLigand module implicit in AutoDockTools [52].

In this study, AutoDock software version 4.2.6 was employed to perform molecular docking. AutoDock incorporates limited flexibility in the receptor, and it combines an empirical free-energy force field with a Lamarckian Genetic Algorithm, providing fast prediction of bound conformations with predicted free energies of association. The AutoDockTools 1.5.6 software was employed to prepare the docking procedures. The default spacing of 0.375 Å was adopted for the grid box, and the volume was set as 60 × 60 × 60 Å. The number of runs was set as 100. The region of interest used for AutoDock docking was fixed as X = 0.041, Y = 0.010 and Z = 3.855. After calculations, only the 10 highest-scored poses were returned as a docking result for ligand–cavity configuration. All the obtained results were ranked according to their score values and presented in kcal/mol. Molecular docking details were visualized using the BIOVIA Discovery Studio virtual environment [53].

### 3.4. Molecular Dynamic Simulations 

Sybyl-X 2.0/Certara software package running on an HP workstation with a Debian 6.0 operating system was used to perform the majority of the molecular modeling/dynamic simulations in ligand–protein systems. The RedMD 2.3 program was employed for the macromolecular simulations [44,45,46].

## 4. Conclusions

The 3-carboxyacylbetulinic acid derivatives reported in the paper were formed by attaching a phosphorus substituent directly to the triterpene system. The diethylphosphate group was introduced at the C30 position (**12a**–**c**), while the diethylphosphonate group at the C29 (**13a**–**c**) or C30 position (**14a**–**c**). The compound **14a** described in the work shows an in vitro anti-HIV-1 activity with an IC_50_ value similar to that for the BVM (IC_50_ values are 0.02 µM and 0.03 µM for **14a** and BVM, respectively) and, at the same time, it has a lower cytotoxicity (CC_50_ values are 68 µM and 29 µM for **14a** and BVM, respectively). This effect showed that the introduction of the diethylphosphonate group into the C30 position of 3-(3′,3′-dimethylsuccinyl) betulinic acid increases selectivity of this maturation inhibitor.

Analysis of the obtained results shows that among the in vitro tested phosphorus analogues of BVM, 30-dialkylphosphonate derivatives (**14a**–**c**) are more active against HIV-1 than 30-dialkylphosphates (**12a**–**c**). Introduction of the phosphonate group at the C29 position (**13a**–**c**) provided derivatives with the lowest activity. 

In molecular docking to the CA CTD-SP1 Gag fragment, a molecular target of maturation inhibitors, an increased number of strong interactions between the ligand **14a** and the protein was observed in comparison to interactions between BVM and the protein. This finding suggests that the potential molecular mechanism of **14a** anti-HIV-1 activity is the same as that of BVM, but is additionally enhanced by introduction of phosphonate moiety into the C30 position. The comparable RMSD values are reached at the final step of the MDS, which confirms the relative stability of the ligand–protein systems for compound **14a**.

## Figures and Tables

**Table 1:** A comparison of the alkaline hydrolysis reaction conditions leading to the formation of phosphonate compounds **4**, **5** and **11**.

Compound	Solvent	Temperature (Temp.) [°C]	Time [h]	Yield [%]
**4**	KOH in CH_3_CH_2_OH	Reflux	2	61
**5**	NaOH in CH_3_OH/H_2_O/THF	Room temp.	0.5	61
**11**	NaOH in CH_3_OH/H_2_O/THF	Room temp.	24	66

**Table 2:** Anti-HIV-1 replication activities in human immunodeficiency virus (HIV)-infected CEM-T4 cell line.

Compound		CC_50_ (μM)	IC_50_ (μM)	TI
**bevirimat** **betulinic acid** **betulin**		29 ± 2.43	0.03 ± 0.009	967
		5 ± 2.44	NO	-
		46±3.01	NO	-
**9**	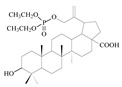	18 ± 1.79	NO	-
**10**	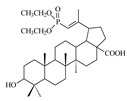	80 ± 1.3	>10	<8
**11**	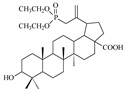	>42	>10	>4
**12a**	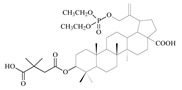	52 ± 1.07	1 ± 0.33	52
**12b**	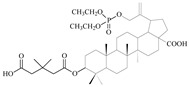	28 ± 1.64	0.6 ± 0.04	47
**12c**	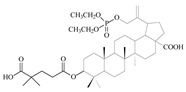	64 ± 1.84	4 ± 1.65	16
**13a**	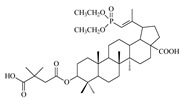	>68	2 ± 0.72	>35
**13b**	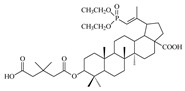	>68	8.8 ± 1.70	>8
**13c**	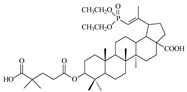	>68	>10	>7
**14a**	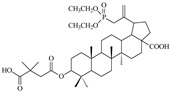	>68	0.02 ± 0.01	3450
**14b**	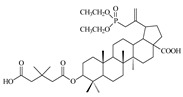	>68	0.9 ± 0.12	>75
**14c**	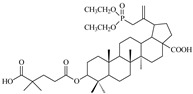	>68	1 ± 0.17	>68

NO: not observed.

**Table 3:** Binding affinities of 3-carboxyacylbetulinic acids (**12a**–**c**, **13a**–**c**, **14a**–**c**).

Compound	12a	12b	12c	13a	13b	13c	14a	14b	14c	BVM
Binding energy ΔG [kcal/mol]	−6.13	−5.61	−7.14	−6.73	−7.55	−8.20	−7.30	−6.89	−6.74	−8.12

Docking solutions with the lowest total score correspond to the highest binding affinities and present the most probable systems in vivo.

**Table 4:** Interactions between BVM, **14a**, and HIV-1 CA CTD-SP1 protein.

Protein		Ligand		Interaction	
Name	Chain: Residue	Name	Residue	Type	Distance [Å]
CTD-SP1 (5I4T)	I:Lys227	BVM	carboxylate	salt bridge	1.90
	J:Lys227		carboxylate	attractive charge	3.67
	I:Pro224		C-21 of betulin	carbon–hydrogen bond	2.90
	J:Lys227		carboxylate	alkyl–alkyl	2.11
	L:Gly223		carboxylate	alkyl–alkyl	2.30
	G:Lys158		C-21 of betulin	alkyl–alkyl	4.69
	L:Pro224		C-21 of betulin	alkyl–alkyl	5.41
	L:Lys158		C-25 of betulin	alkyl–alkyl	5.28
	K:Lys158		C-29 of betulin	alkyl–alkyl	3.67
	K:Lys158		C-30 of betulin	alkyl–alkyl	3.89
	G:Lys158		C-21 of betulin	alkyl–alkyl	4.55
	J:Lys158		C-5′ of BVM	alkyl–alkyl	3.71
	J:Lys158		C-6′ of BVM	alkyl–alkyl	4.76
	I:Lys227	**14a**	carboxylate	salt bridge	1.99
	G:Lys158		carboxylate	carbon–hydrogen bond	2.98
	K:Lys158		carboxylate	carbon–hydrogen bond	5.18
	J:Lys227		phosphonate	conventional hydrogen bond	2.23
	L:Lys158		phosphonate	conventional hydrogen bond	1.97
	J:Lys227		ethyl	carbon–hydrogen bond	2.40
	L:Lys158		phosphonate	carbon–hydrogen bond	2.19
	J:Gly222		ethyl	carbon–hydrogen bond	3.59
	I:Lys158		C-25 of betulin	alkyl–alkyl	5.23
	K:Lys158		C-24 of betulin	alkyl–alkyl	4.94
	I:Lys158		C-7 of betulin	alkyl–alkyl	4.38
	H:Lys158		ethyl	alkyl–alkyl	5.23
	J:Pro224		ethyl	alkyl–alkyl	4.08
	H:Lys158		ethyl	alkyl–alkyl	3.69

## Supplementary Materials

Supplementary materials can be found at https://www.mdpi.com/1422-0067/20/20/5209/s1.

## Abbreviations

BVM	Bevirimat
EC_50_	Half maximal effective concentration—the concentration of a compound at which 50% of the Population exhibit a response
TI	Therapeutic index
CC_50_	Cytotoxicity concentration of 50%—concentration required for the reduction of cell viability by 50%
IC_50_	Half maximal inhibitory concentration—the concentration of a compound required to inhibit 50% of a specific biological or biochemical function
HSV	Herpes simplex virus
HBV	Hepatitis B virus
ELISA	Enzyme-linked immunosorbent assay
MTT	3-(4,5-Dimethylthiazol-2-yl)-2 5-diphenyltetrazolium bromide
