# Supplementary material for: New Phosphorus Analogs of Bevirimat: Synthesis, Evaluation of Anti-HIV-1 Activity and Molecular Docking Study
Source: Int J Mol Sci. 2019 Oct 21;20(20):5209. doi: 10.3390/ijms20205209 (PMC6829466; doi:10.3390/ijms20205209)
Supplement: Supplementary file 1 [file ijms-20-05209-s001.pdf]

New Phosphorus Analogs of Bevirimat: Synthesis, Evaluation of Anti-HIV Activity and Molecular Docking Study

Elwira Chrobak[1*], *Krzysztof Marciniec*[1], *Aleksandra Dąbrowska*[2], *Paweł Pęcak*[1], *Ewa Bębenek*[1], *Monika Kadela-Tomanek*[1], *Andrzej Bak*[3], *Maria Jastrzębska*[3], *Stanisław Boryczka*[1]

[1] Medical University of Silesia in Katowice, School of Pharmacy with the Division of Laboratory Medicine in Sosnowiec, Department of Organic Chemistry, 4 Jagiellońska Str., 41-200 Sosnowiec, Poland; kmarciniec@sum.edu.pl (K.M.); pawel.marek.pecak@gmail.com (P.P.); ebebenek@sum.edu.pl (E.B.); mkadela@sum.edu.pl (M.K.-T.); boryczka@sum.edu.pl (S.B.)

[2] National Medicines Institute, 30/34 Chełmska Str., 00-725 Warszawa, Poland; aleksandra_dabrowska@o2.pl (A.D.)

[3] Institute of Chemistry, University of Silesia, 9 Szkolna Str., 40-007 Katowice, Poland; andrzej.bak@us.edu.pl (A.B.);

[4] Silesian Center for Education and Interdisciplinary Research, University of Silesia, Institute of Physics, Department of Solid State Physics, 75 Pułku Piechoty 1a, 41-500 Chorzów, Poland; maria.jastrzebska@us.edu.pl (M.J.)

*Corresponding author

E-mail address: echrobak@sum.edu.pl

Characteristics of synthesized compounds

3-Acetyl-30-diethoxyphosphorylbetulin **5** (^{1}H, ^{13}C and ^{31}P NMR spectra) …………………….. **3**

30-Diethoxyphosphoryloxybetulonic acid **6** (^{1}H, ^{13}C and ^{31}P NMR spectra)…………………..... **5**

29-Diethoxyphosphorylbetulonic acid **7 (**^{1}H, ^{13}C and ^{31}P NMR spectra) …………………….…..... **7**

3-*O*-Acetyl-30-diethoxyphosphorylbetulinic acid **8 (**^{1}H, ^{13}C and ^{31}P NMR spectra).………..… **9**

30-Diethoxyphosphorylbetulinic acid **9** (^{1}H, ^{13}C and ^{31}P NMR spectra) ……………………….. **11**

29-Diethoxyphosphorylbetulinic acid **10** (^{1}H, ^{13}C and ^{31}P NMR spectra) ……………………… **13**

30-Diethoxyphosphorylbetulinic acid **11** (^{1}H, ^{13}C and ^{31}P NMR spectra) ……………………… **15**

30-Diethoxyphosphoryloxy-3-*O*-(3’,3’-dimethylsuccinyl)betulinic acid **12a (**^{1}H, ^{13}C and ^{31}P NMR spectra) ………..…………………………………………….......................... **17**

30-Diethoxyphosphoryloxy-3-*O*-(3’,3’-dimethylglutaryl)betulinic acid **12b** (^{1}H, ^{13}C and ^{31}P NMR spectra)……………………...………………………………………………….. **19**

30-Diethoxyphosphoryloxy-3-*O*-(4’,4’-dimethylglutaryl)betulinic acid **12c** (^{1}H, ^{13}C and ^{31}P NMR spectra)…………………. …………………………………………………... **21**

29-Diethoxyphosphoryl-3-*O*-(3’,3’-dimethylsuccinyl)betulinic acid **13a** (^{1}H, ^{13}C and ^{31}P NMR spectra) ……………………………………………………………………………….. **23**

29-Diethoxyphosphoryl-3-*O*-(3’,3’-dimethylglutaryl)betulinic acid **13b** (^{1}H, ^{13}C and ^{31}P NMR spectra) ……………………………………………………………………………… **25**

29-Diethoxyphosphoryl-3-*O*-(4’,4’-dimethylglutaryl)betulinic acid **13c** (^{1}H, ^{13}C and ^{31}P NMR spectra) ……………………………………………………………………………….. **27**

30-Diethoxyphosphoryl-3-*O*-(3’,3’-dimethylsuccinyl)betulinic acid **14a (**^{1}H, ^{13}C and ^{31}P NMR spectra) ……………………………………………………………………………….. **29**

30-Diethoxyphosphoryl-3-*O*-(3’,3’-dimethylglutaryl)betulinic acid **14b** (^{1}H, ^{13}C and ^{31}P NMR spectra) ……………………………………………………………………………… **31**

30-Diethoxyphosphoryl-3-*O*-(4’,4’-dimethylglutaryl)betulinic acid **14c** (^{1}H, ^{13}C and ^{31}P NMR spectra) ……………………………………………………………………………….. **33**

Charts of changes in cytotoxicity of compounds in the tested concentration range…...….. **35**

3-Acetyl-30-diethoxyphosphorylbetulin 5

Yield 61%; mp 118-122 °C; R_f = 0.37 (chloroform/ethanol, 15:1, v/v); IR (KBr) ν (cm^{-1}): 3439, 2941, 1732, 1246, 1028, 648; ^{1}H NMR ($CDCl_3$) δ (ppm): 4.96 (m, 1H, H29), 4.92 (m, 1H, H29), 4.40 (m, 1H, H3), 4.05 (m, 4H, 2 x OCH_2CH_3), 3.72 (m, 1H, H28), 3.25 (m, 1H, H28), 2.50 (m, 2H, H30), 2.40 (m, 1H, H19), 1.98 (s, 3H, $C(O)CH_3$), 1.25 (m, 6H, 2 x OCH_2CH_3), 1.20 – 2.05 (m, 23H, CH, CH_2), 0.95 (s, 3H, CH_3), 0.90 (s, 3H, CH_3), 0.78 (s, 6H, 2 x CH_3), 0.76 (s, 3H, CH_3), 0.72 (m, 1H, H5); ^{13}C NMR ($CDCl_3$) δ (ppm): 171.1, 151.1, 112.9, 80.9, 71.2, 68.3, 61.9, 60.5, 58.5, 55.3, 50.2, 50.1, 47.7, 42.7, 41.0, 40.9, 38.4, 37.8, 37.1, 37.0, 34.1, 33.9, 33.6, 29.2, 27.9, 27.0, 26.9, 23.7, 21.3, 20.9, 18.4, 18.1, 16.4, 16.2, 16.0, 14.7; ^{31}P NMR ($CDCl_3$) δ (ppm): 27.8; HR-MS (APCI) *m/z*: $C_{36}H_{60}O_6P$ [$(M–H)^-$], Calc. 619.4128; Found 619.4136.

^{1}H NMR

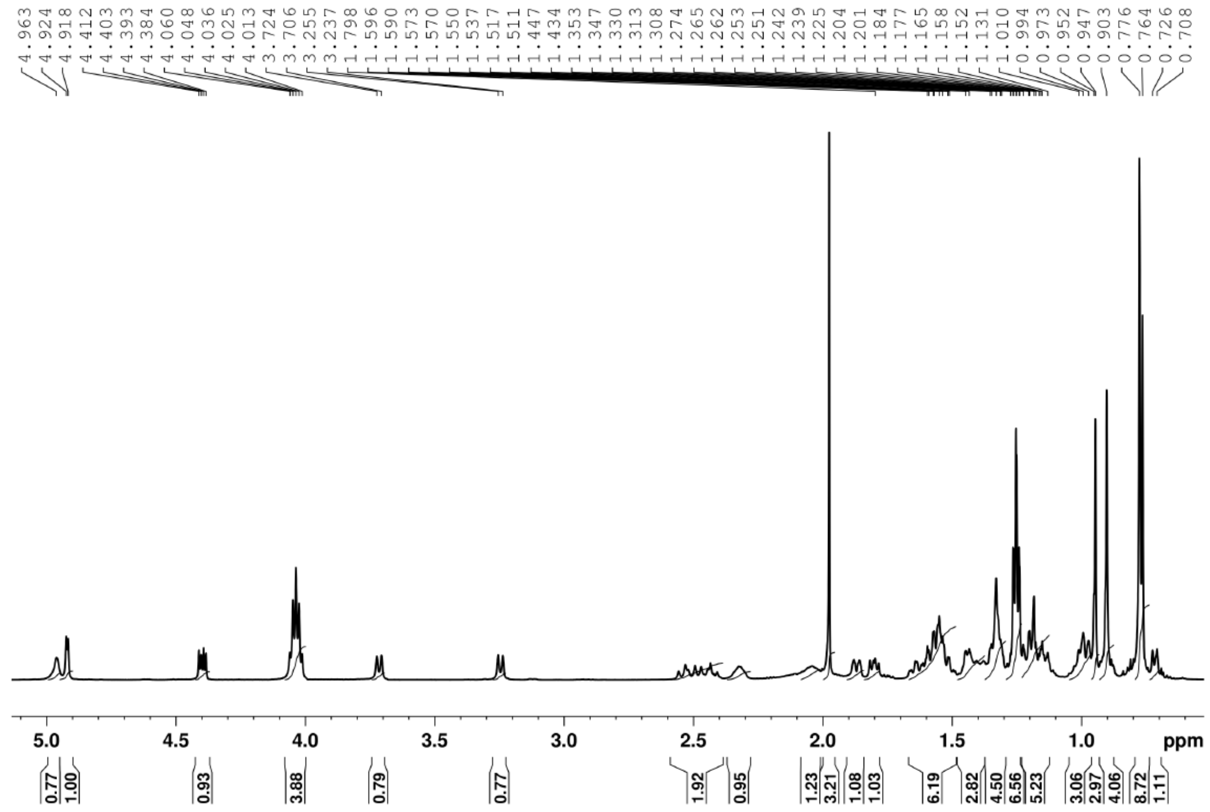

^{13}C NMR

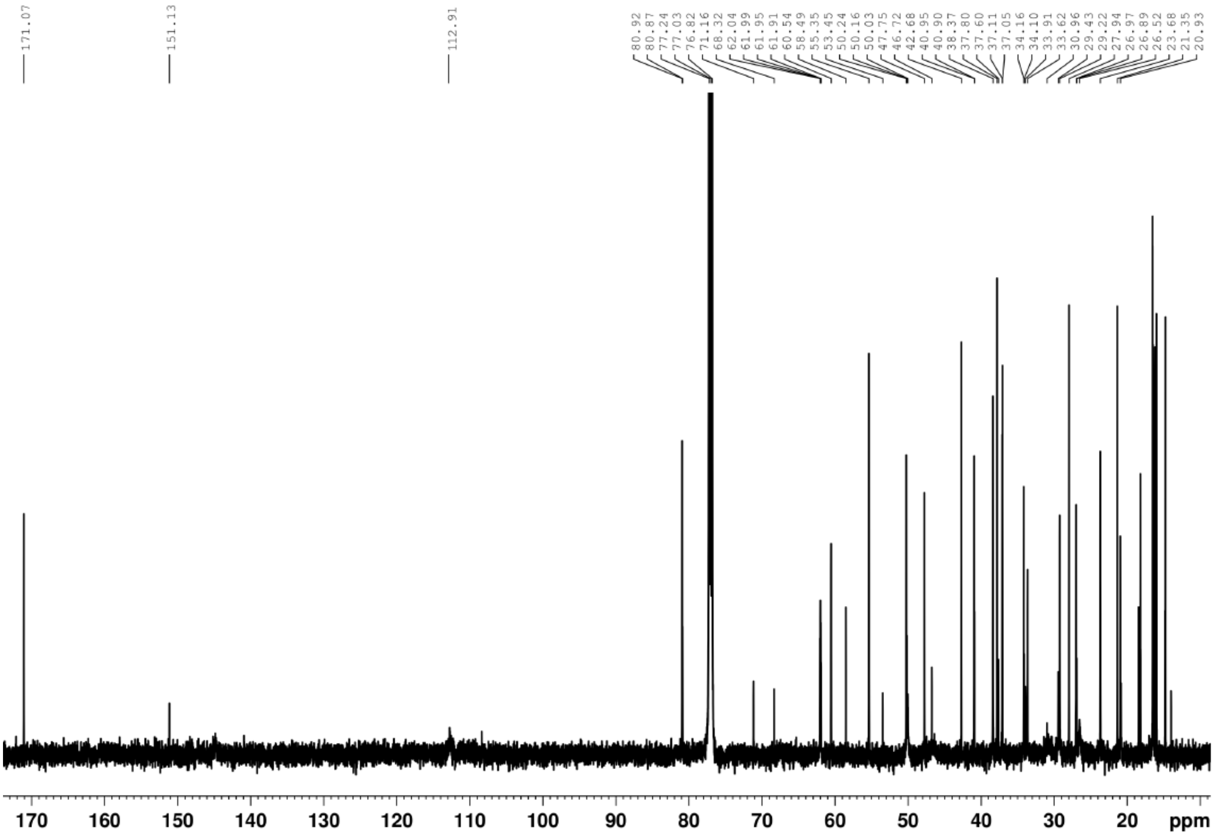

^{31}P NMR

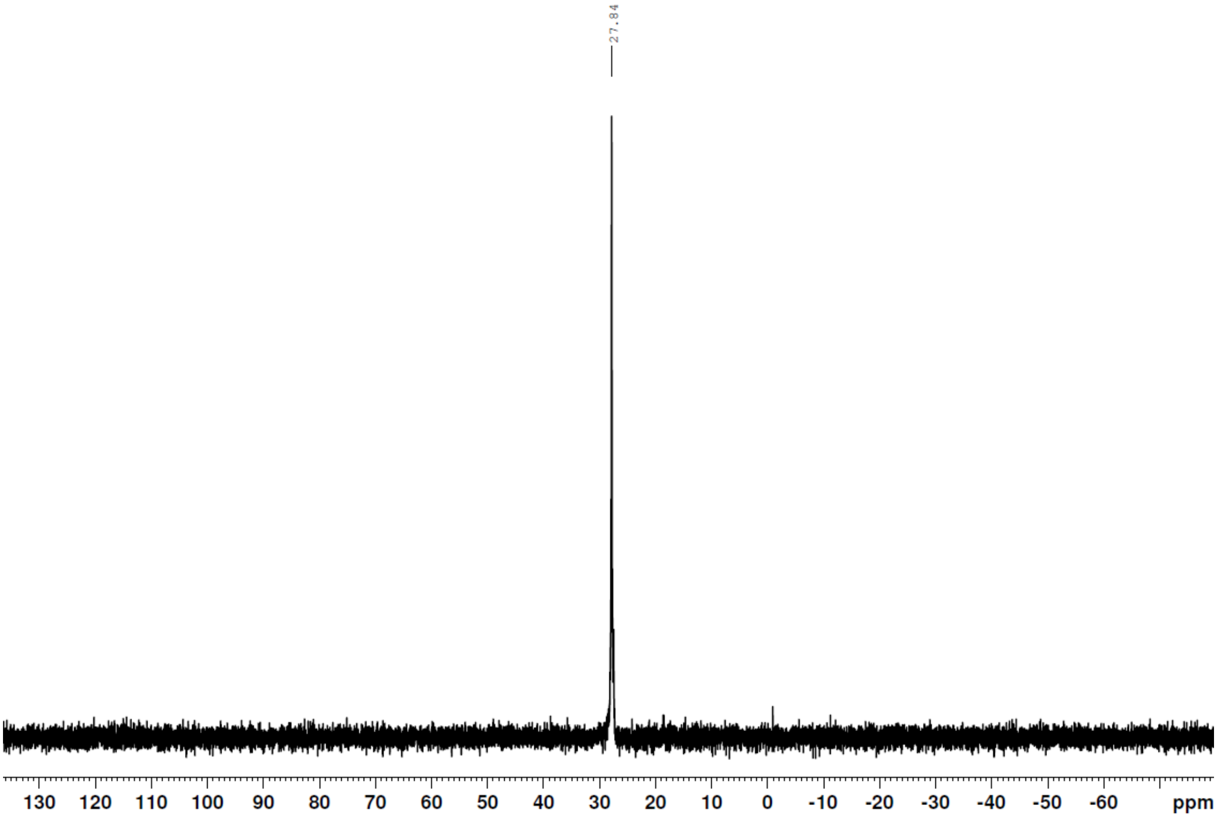

30-Diethoxyphosphoryloxybetulonic acid 6

Yield 62%; mp 220-223 °C; R_f = 0.33 (chloroform/ethanol, 15:1, v/v); IR (KBr) ν (cm^{-1}): 2967, 1701, 1257, 1039; ^1H NMR ($CDCl_3$) δ (ppm): 4.98 (m, 1H, H29), 4.93 (m, 1H, H 29), 4.44 (d, *J*=6 Hz, 2H, H30), 4.07 (m, 4H, OCH_2CH_3), 2.85 (m, 1H, H19), 0.90 – 2.55 (m, 24 H, CH, CH_2), 1.27 (m, 6H, OCH_2CH_3), 1.02 (s, 3H, CH_3), 0.96 (s, 3H, CH_3), 0.94 (s, 3H, CH_3), 0.89 (s, 3H, CH_3), 0.85 (s, 3H, CH_3); ^{13}C NMR ($CDCl_3$) δ (ppm): 218.2, 179.9, 149.4, 109.8, 69.1, 63.9, 63.9, 56.2, 54.9, 49.8, 47.3, 42.4, 42.2, 40.6, 39.6, 38.4, 36.9, 36.7; 34.1, 33.6, 32.0, 31.9, 29.7, 26.7, 26.6, 21.5, 21.0, 19.6, 16.2, 16.2, 16.0, 15.8, 14.6; ^{31}P NMR ($CDCl_3$) δ (ppm): -0.97; HR-MS (APCI) *m/z*: $C_{34}H_{54}O_7P$ [(M–H)$^-$], Calc. 605.3607; Found 605.3617.

^1H NMR

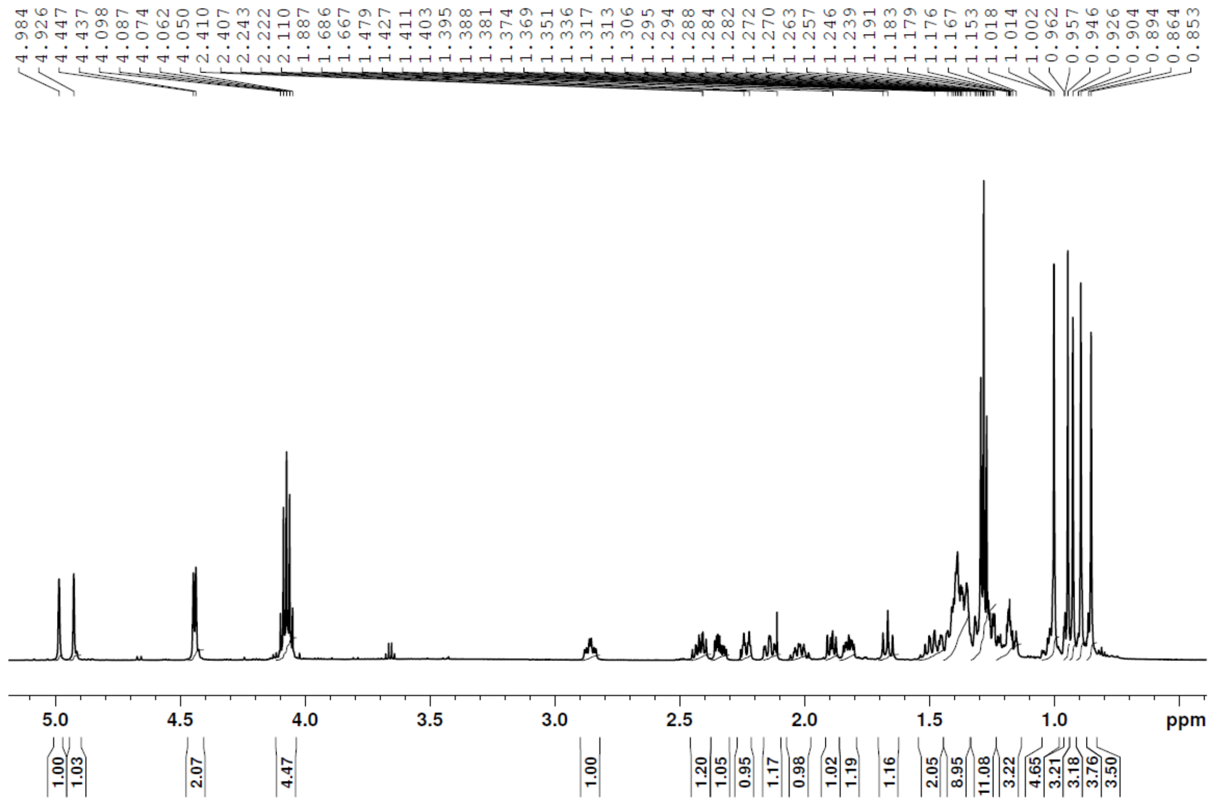

^{13}C NMR

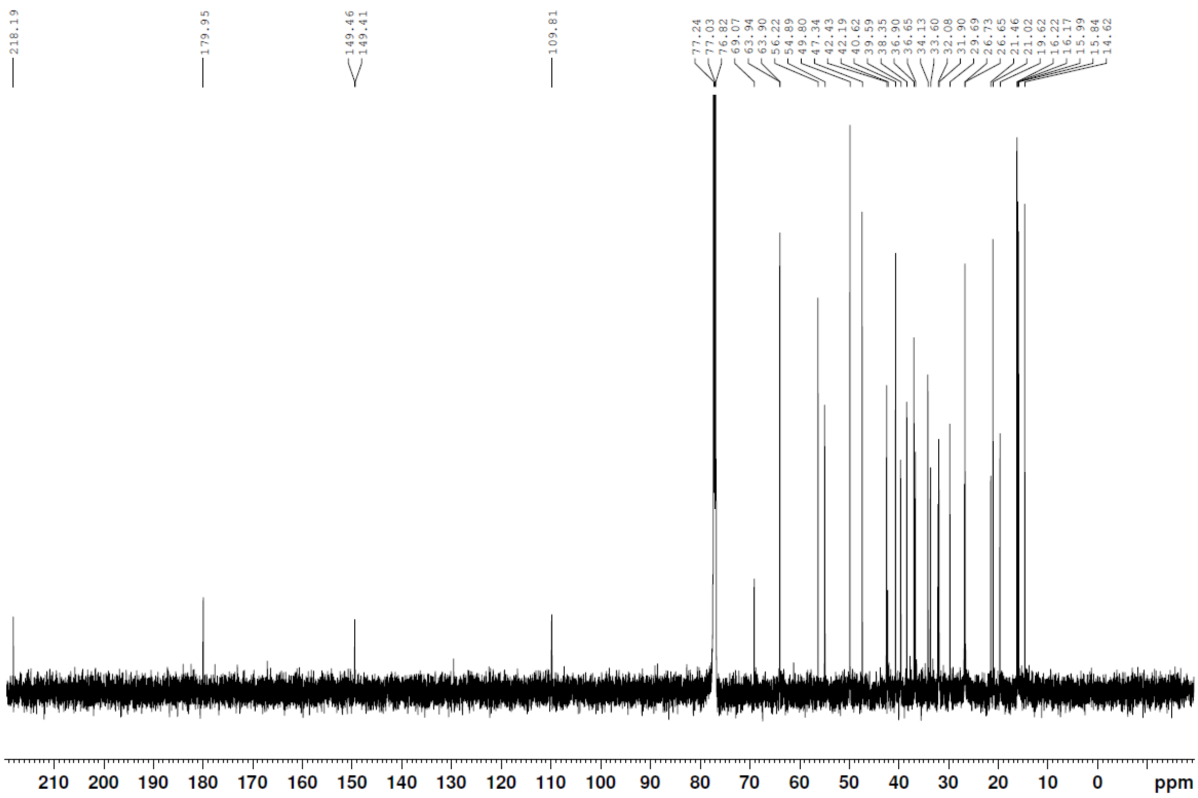

^{31}P NMR

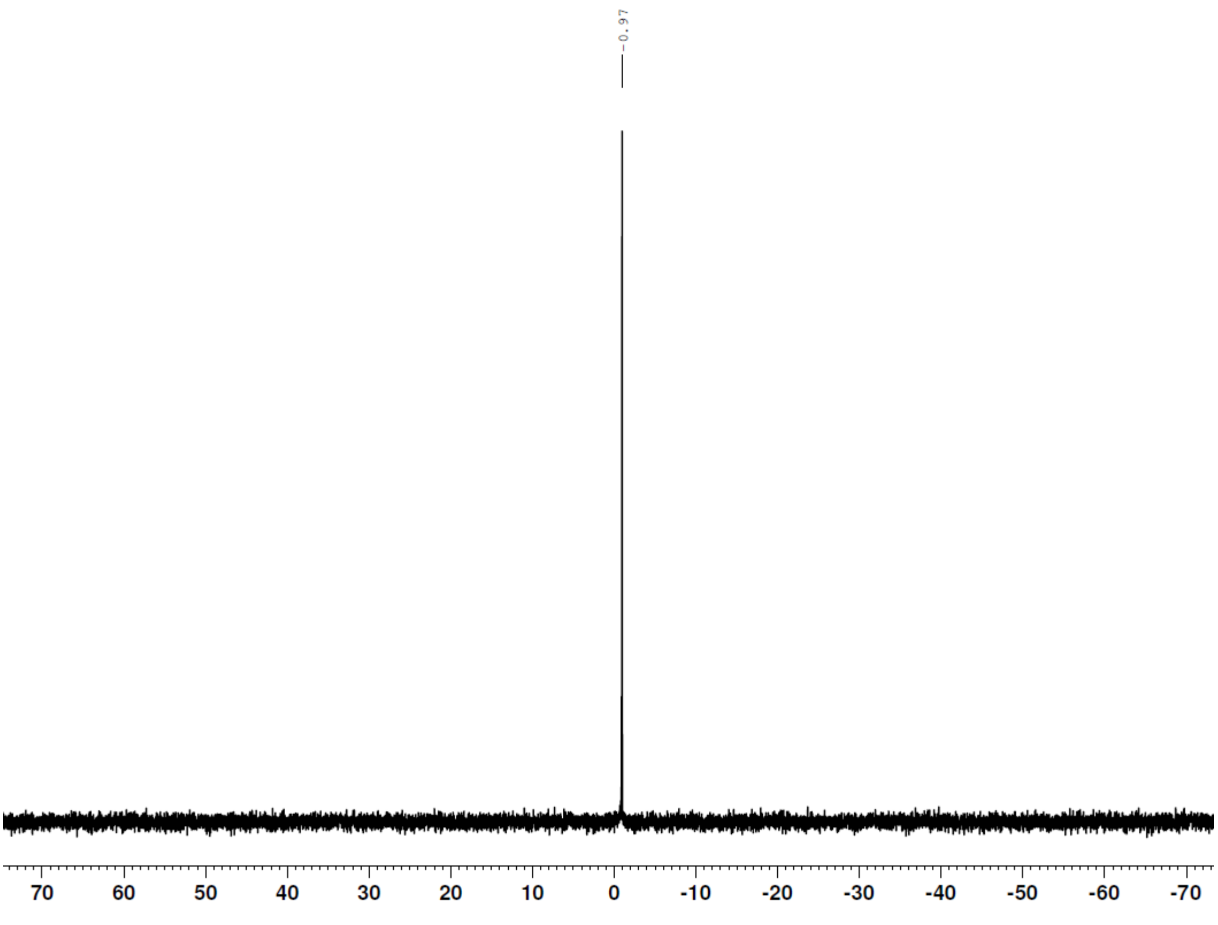

29-Diethoxyphosphorylbetulonic acid 7

Yield 31%; mp 244-245 °C, R_f = 0.56 (chloroform/ethanol, 15:1, v/v); IR (KBr) ν (cm^{-1}): 2956, 1717, 1236, 972, 799; ^1H NMR ($CDCl_3$) δ (ppm): 5.52 (d, 1H, $^2J_{PH}$=18.6 Hz, H29), 4.06 (m, 4H, 2x OCH_2CH_3), 3.13 (m, 1H, H19), 2.50 (m, 1H, H2), 2.45 (m, 1H, H2), 2.08 (d, *J*=3 Hz, 3H, H30), 0.93–2.36 (m, 22H, CH, CH_2), 1.33 (m, 6H, 2 x OCH_2CH_3), 1.10 (s, 3H, CH_3), 1.04 (s, 3H, CH_3), 1.00 (s, 3H, CH_3), 0.99 (s, 3H, CH_3), 0.94 (s, 3H, CH_3); ^{13}C NMR ($CDCl_3$) δ (ppm): 218.0, 179.8, 167.5, 111.2, 61.3, 61.2, 58.5, 56.2, 54.9, 50.7, 50.5, 50.0, 49.7, 47.3, 42.5, 40.6, 39.5, 38.2, 37.0, 36.9, 34.1, 33.6, 32.0, 30.5, 29.6, 26.7, 25.9, 21.3, 21.0, 19.6, 16.4, 16.0, 15.8, 14.6; ^{31}P NMR ($CDCl_3$) δ (ppm): 18.75; HR-MS (APCI) *m/z*: $C_{34}H_{54}O_6P$ [$(M–H)^-$], Calc. 589.3658; Found 589.3664.

^1H NMR

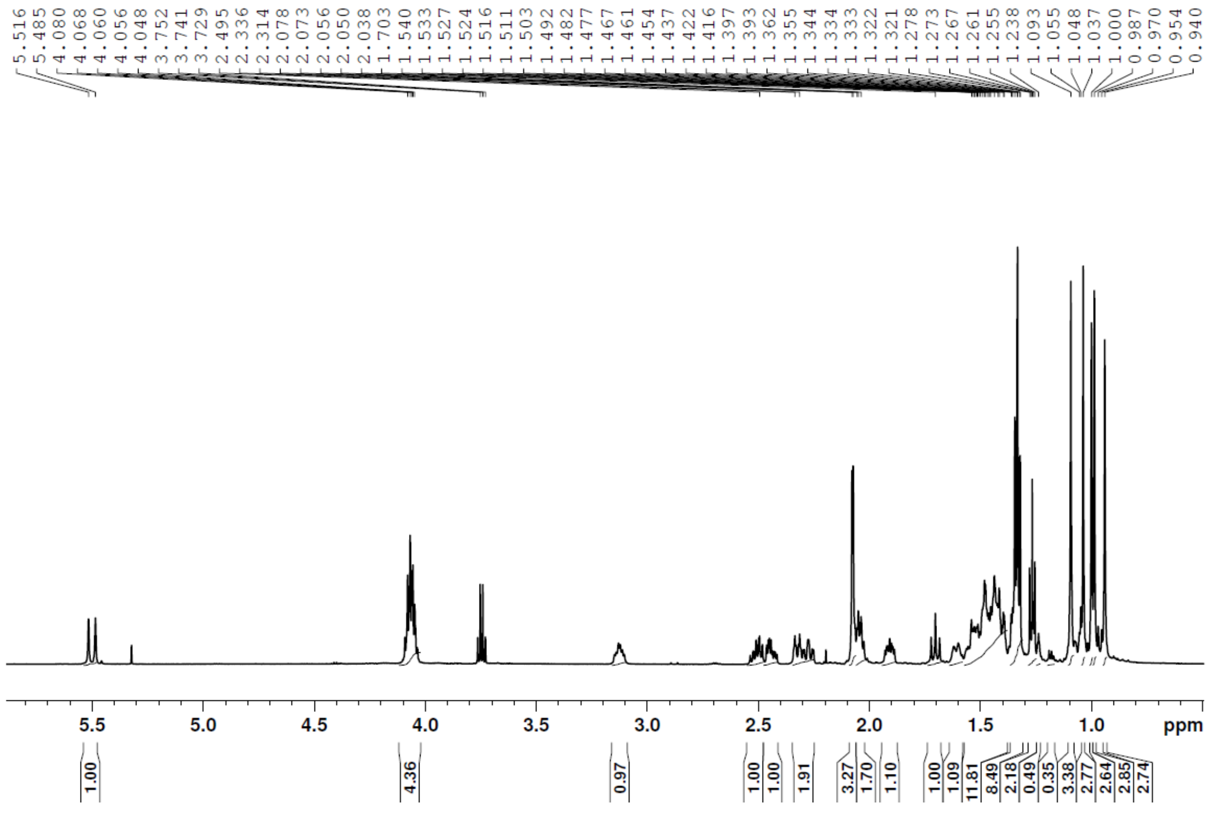

^{13}C NMR

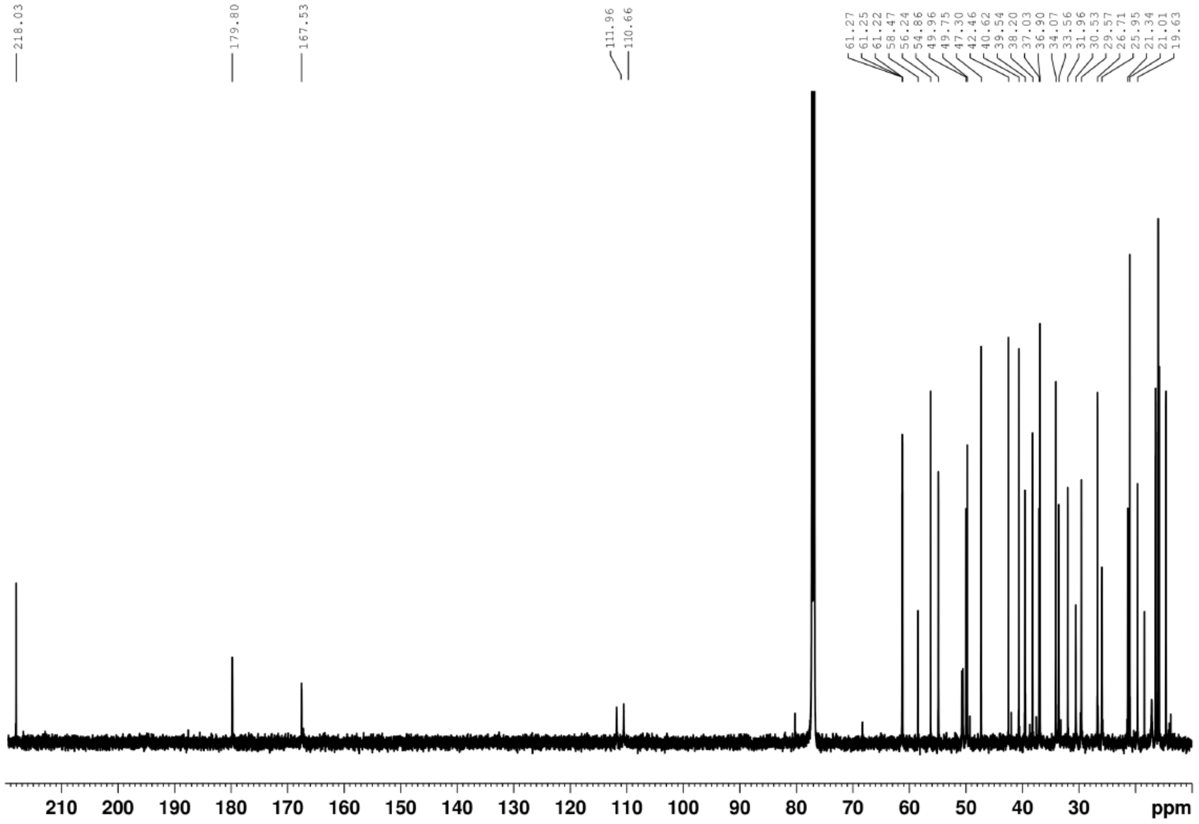

^{31}P NMR

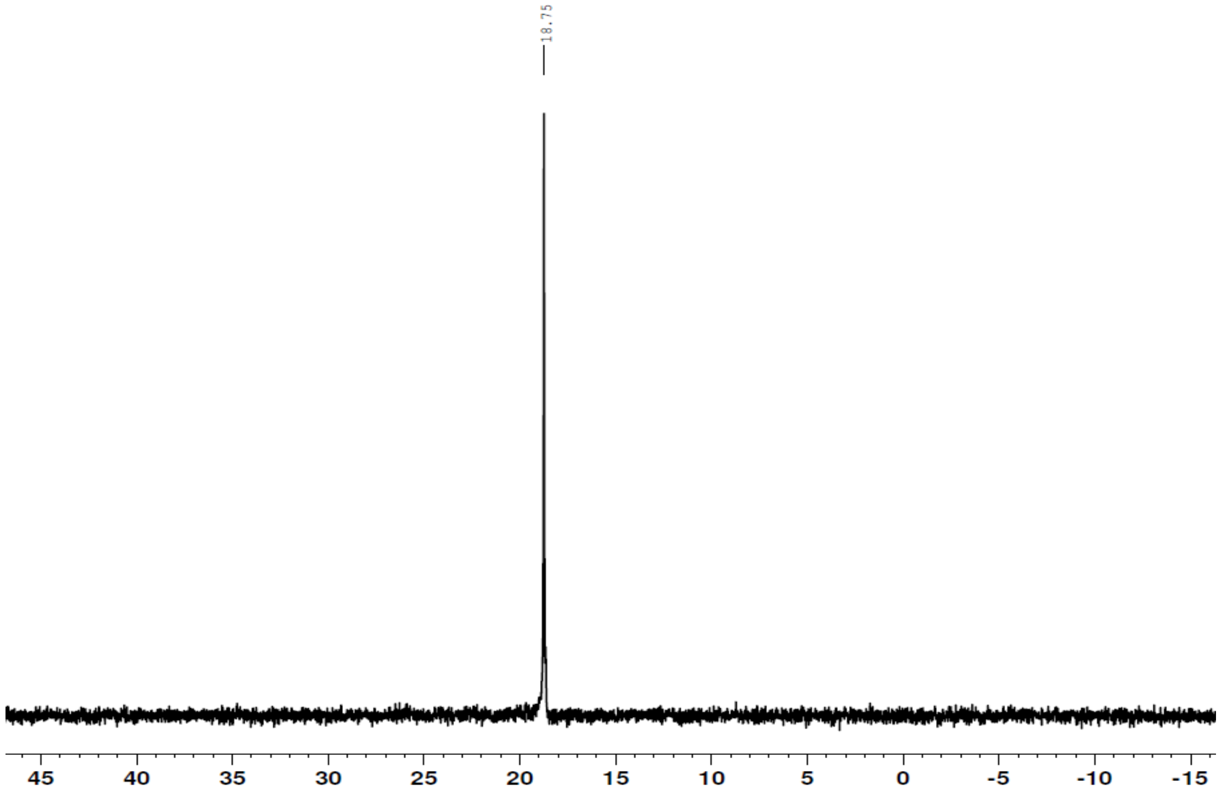

3-O-Acetyl-30-diethoxyphosphorylbetulinic acid 8

Yield 63%; mp 231-233 °C; R_f = 0.35 (chloroform/ethanol, 15:1, v/v); IR (KBr) ν (cm^{-1}): 3434, 2943, 1738, 1246, 1024, 753; ^{1}H NMR ($CDCl_3$) δ (ppm): 5.07 (m, 1H, H29), 5.01 (m, 1H, H29), 4.53 (m, 2H, H30), 4.41 (m, 1H, H3), 4.16 (m, 4H, 2 x OCH_2CH$_3$), 2.94 (m, 1H, H19), 2.09 (s, 3H, C(O)CH$_3$), 1.20 – 2.35 (m, 23 H, CH, CH$_2$), 1.37 (m, 6H, 2 x OCH$_2$CH_3), 1.00 (s, 3H, CH$_3$), 0.99 (s, 3H, CH$_3$), 0.94 (s, 3H, CH$_3$), 0.84 (s, 3H, CH$_3$), 0.77 (s, 3H, CH$_3$), 0.70 (m, 1H, H5); ^{13}C NMR ($CDCl_3$) δ (ppm): 178.5, 171.0, 151.1, 112.9, 80.9, 61.9, 58.5, 56.2, 56.1, 55.4, 50.4, 50.3, 42.4, 40.7, 38.4, 38.3, 38.1, 37.8, 37.1, 36.7, 36.3, 34.3, 32.1, 30.9, 29.6, 27.9, 23.7, 21.3, 21.0, 18.4, 18.2, 16.4, 16.2, 16.0, 14.7; ^{31}P NMR ($CDCl_3$) δ (ppm): 28.0; HR-MS (APCI) *m/z*: $C_{36}H_{58}O_7P$ [$(M–H)^-$], Calc. 633.3920; Found 633.3913.

^{1}H NMR

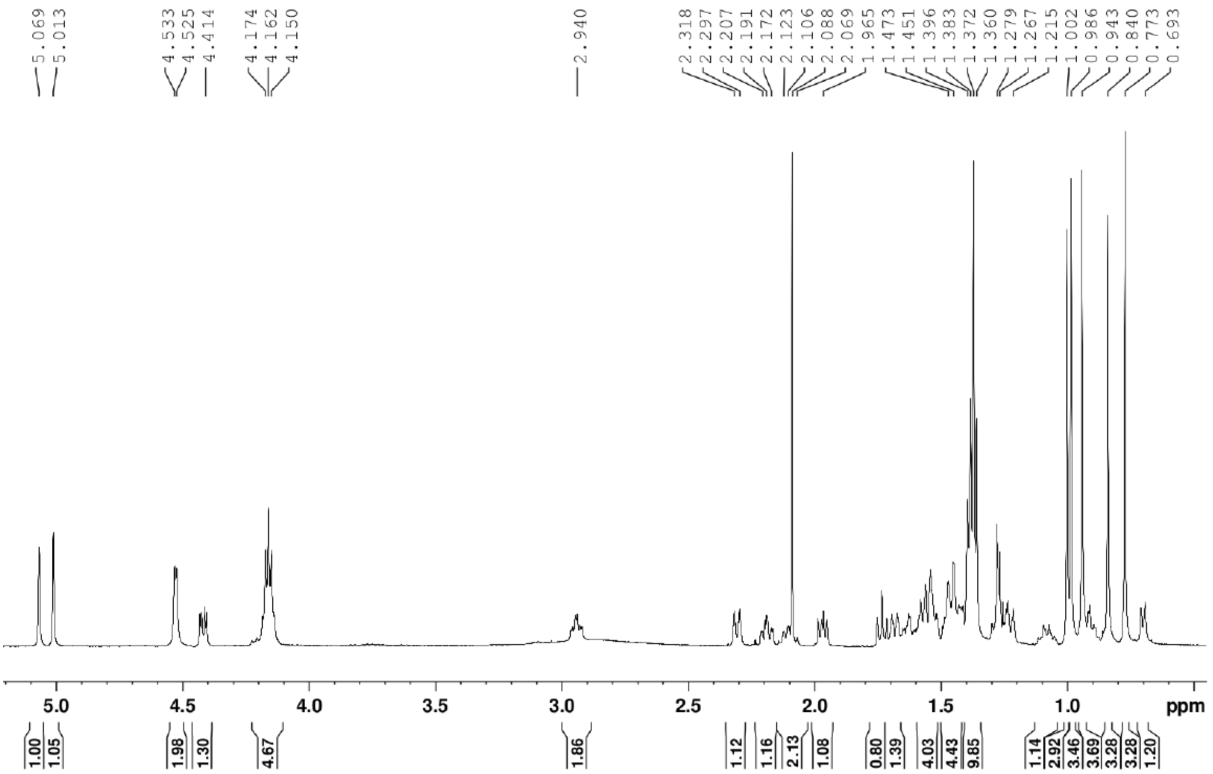

^{13}C NMR

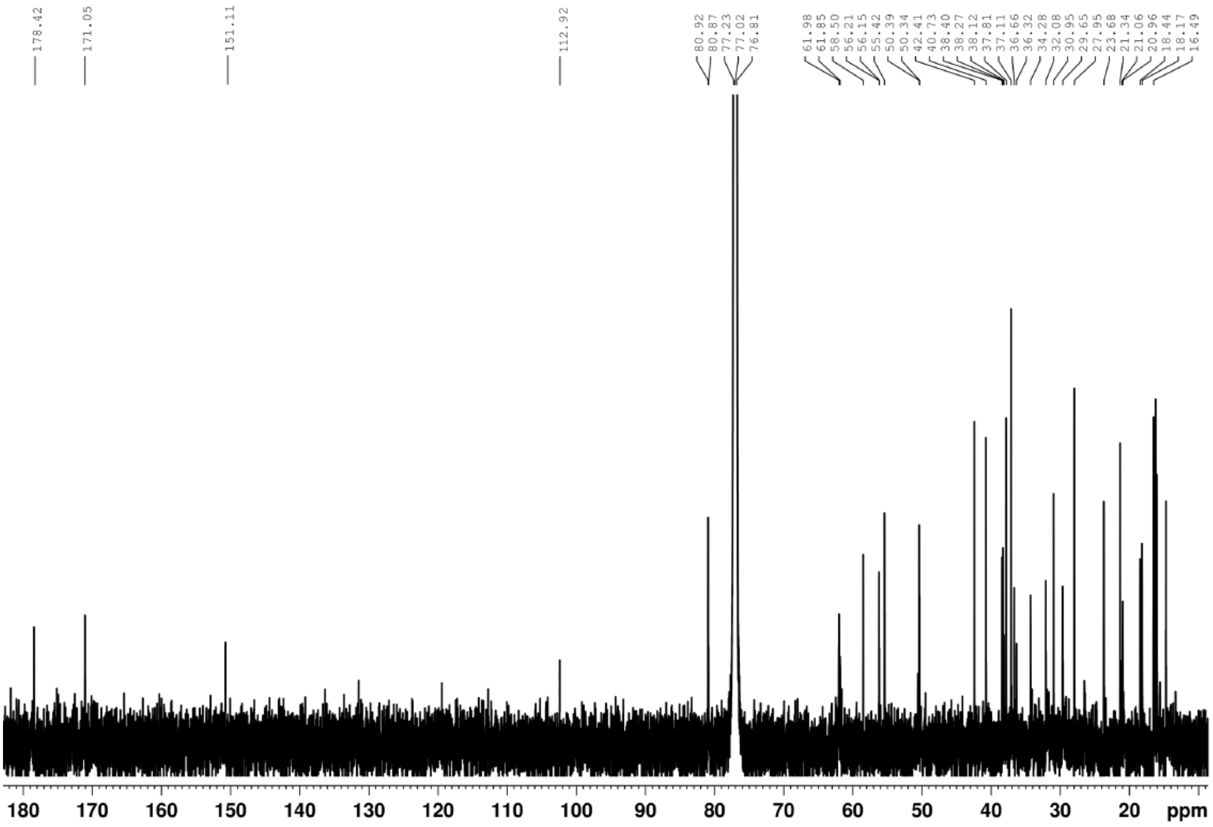

^{31}P NMR

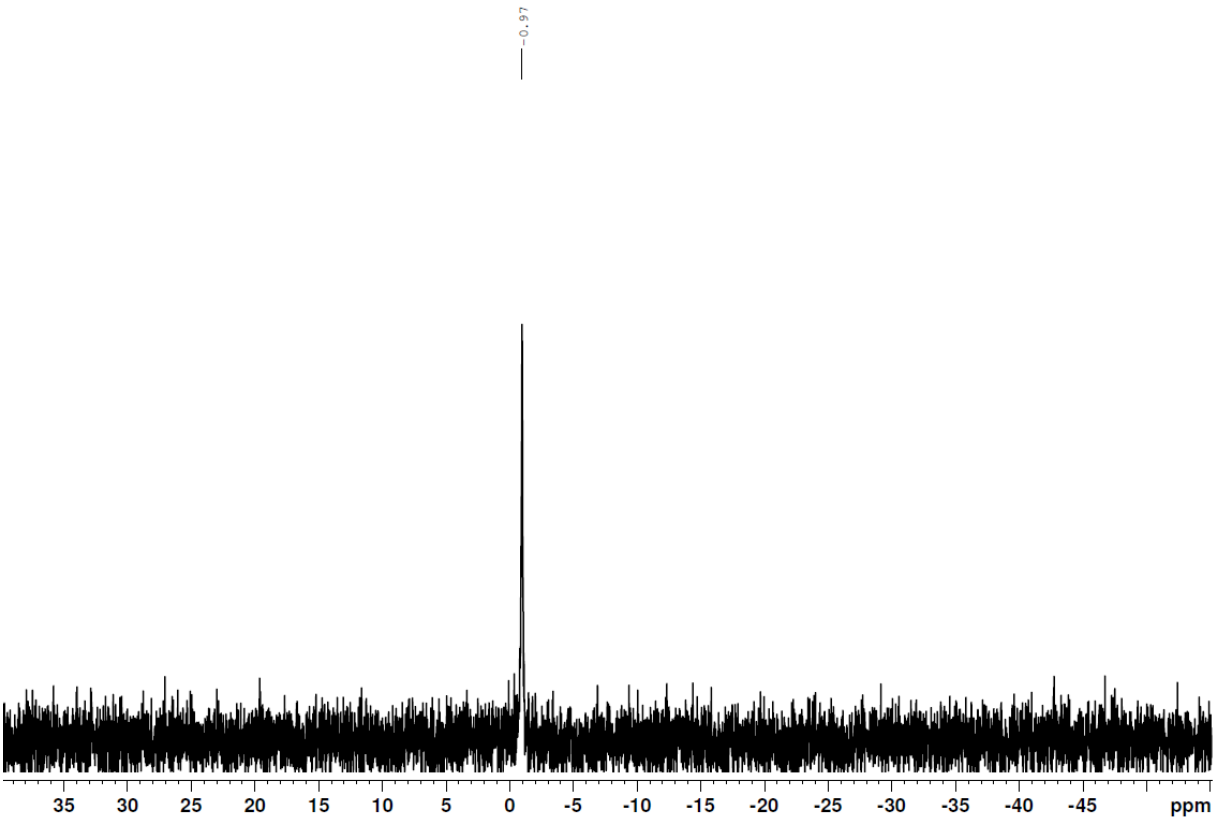

30-Diethoxyphosphorylbetulinic acid 9

Yield 66%; mp 264-266 °C; R_f = 0.31 (chloroform/ethanol, 15:1, v/v); IR (KBr) ν (cm^{-1}): 3431, 2941, 1716, 1244, 1030; ^{1}H NMR ($CDCl_3$) δ (ppm): 5.07 (m, 1H, H29), 5.01 (m, 1H, H29), 4.52 (d, 2H, H30), 4.16 (m, 4H, OCH_2CH_3), 3.21 (m, 1H, H3), 2.95 (m, 1H, H19), 1.38 (m, 6H, OCH_2CH_3), 0.75 – 2.35 (m, 23 H, CH, CH_2), 1.00 (s, 3H, CH_3), 0.98 (s, 3H, CH_3), 0.94 (s, 3H, CH_3), 0.84 (s, 3H, CH_3), 0.78 (s, 3H, CH_3), 0.70 (m, 1H, H-5); ^{13}C NMR ($CDCl_3$) δ (ppm): 180.3, 149.5, 109.7, 79.0, 69.1, 63.9, 63.9, 56.2, 55.3, 50.4, 49.9, 42.4, 42.3, 40.7, 38.9, 38.7; 38.3, 37.2, 36.7, 34.3, 32.1, 32.0, 29.7, 28.0, 27.3, 26.7, 20.9, 18.3, 16.2, 16.2, 16.1, 16.0, 15.4, 14.7; ^{31}P NMR ($CDCl_3$) δ (ppm): -0.98; HR-MS (APCI) *m/z*: $C_{34}H_{56}O_7P$ $[(M-H)^-]$, Calc. 607.3764; Found 607.3757.

^{1}H NMR

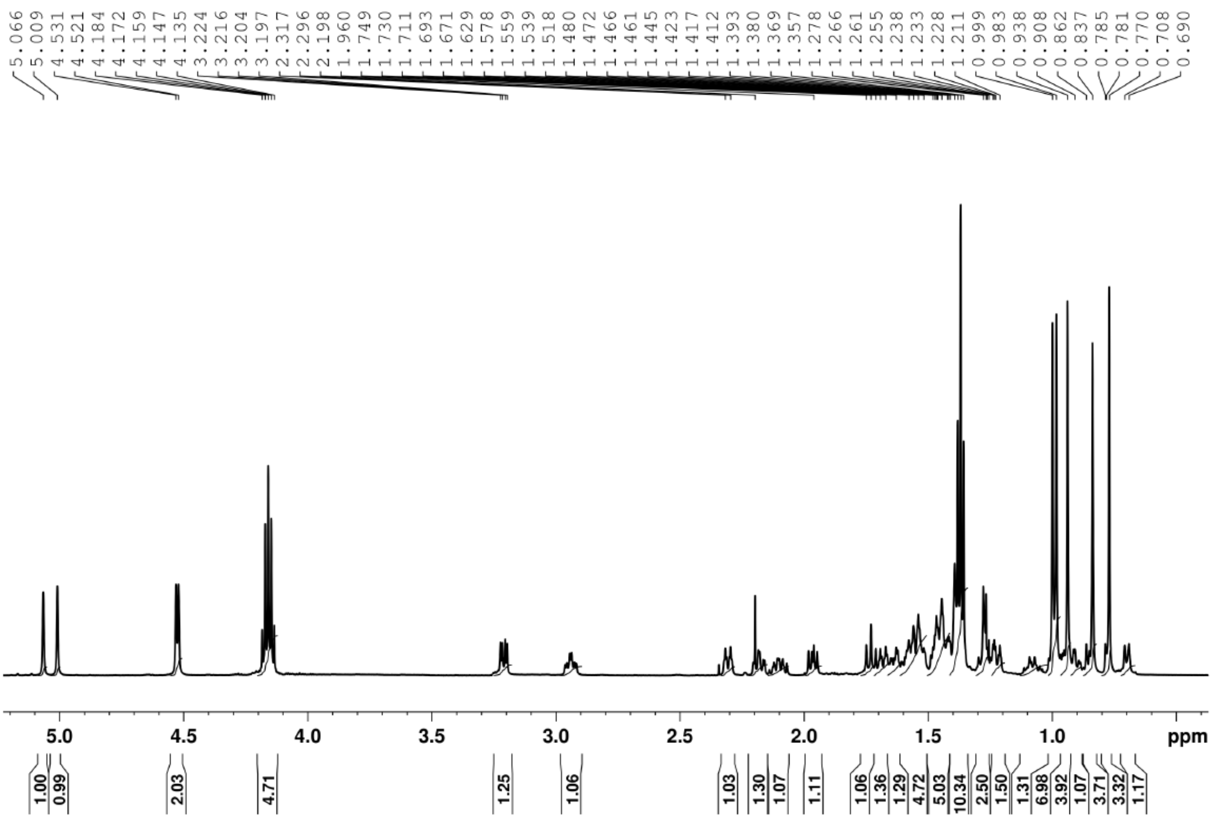

^{13}C NMR

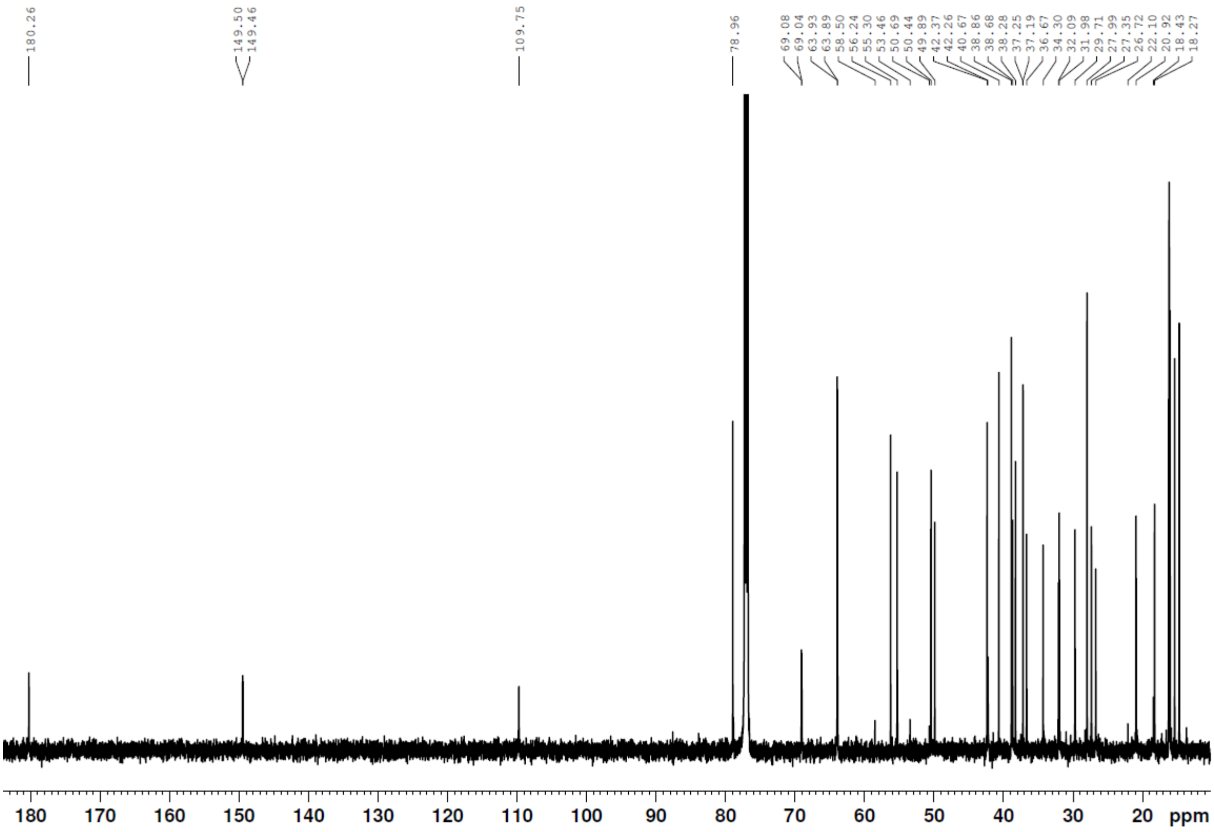

^{31}P NMR

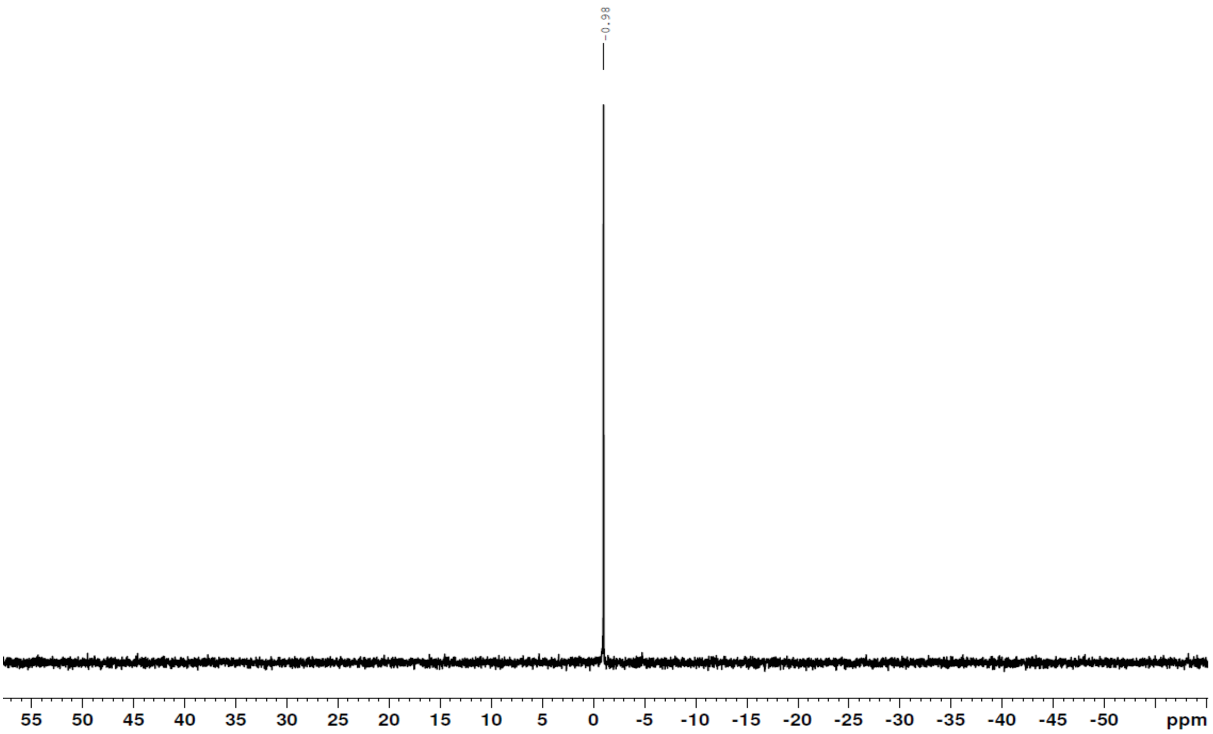

29-Diethoxyphosphorylbetulinic acid 10

Yield 50%; mp 271-273 °C; R_f = 0.31 (chloroform/ethanol, 15:1, v/v); IR (KBr) ν (cm^{-1}): 3417, 1707, 1227, 1027, 751; ^1H NMR ($CDCl_3$) δ (ppm): 5.49 (d, 1H, $^2J_{PH}$=18.6, Hz, H29), 4.06 (m, 4H, 2x OC$H_2$$CH_3$), 3.22 (m, 1H, H3), 3.12 (m, 1H, H19), 2.08 (d, *J*=3Hz, 3H, H30), 0.90– 2.10 (m, 23 H, CH, CH_2), 1.34 (m, 6H, 2 x $OCH_2C$$H_3$), 0.99 (s, 6H, 2 x CH_3), 0.95 (s, 3H, CH_3), 0.84 (s, 3H, CH_3), 0.78 (s, 3H, CH_3), 0.71 (m, 1H, H5); ^{13}C NMR ($CDCl_3$) δ (ppm): 179.7, 167.7, 111.2, 78.9, 61.2, 56.2, 55.3, 50.7, 50.4, 50.0, 42.4, 40.7, 38.9, 38.7, 38.1, 38.1, 37.2, 37.0, 34.3, 32.0, 30.5, 29.6, 28.0, 27.3, 26.0, 20.8, 18.3, 17.1, 16.4, 16.1, 16.0, 15.4, 14.7;^{31}P NMR ($CDCl_3$) δ (ppm): 18.80; HR-MS (APCI) *m/z*: $C_{34}H_{56}O_6P$ [(M–H)$^-$], Calc. 591.3815; Found 591.3823.

^1H NMR

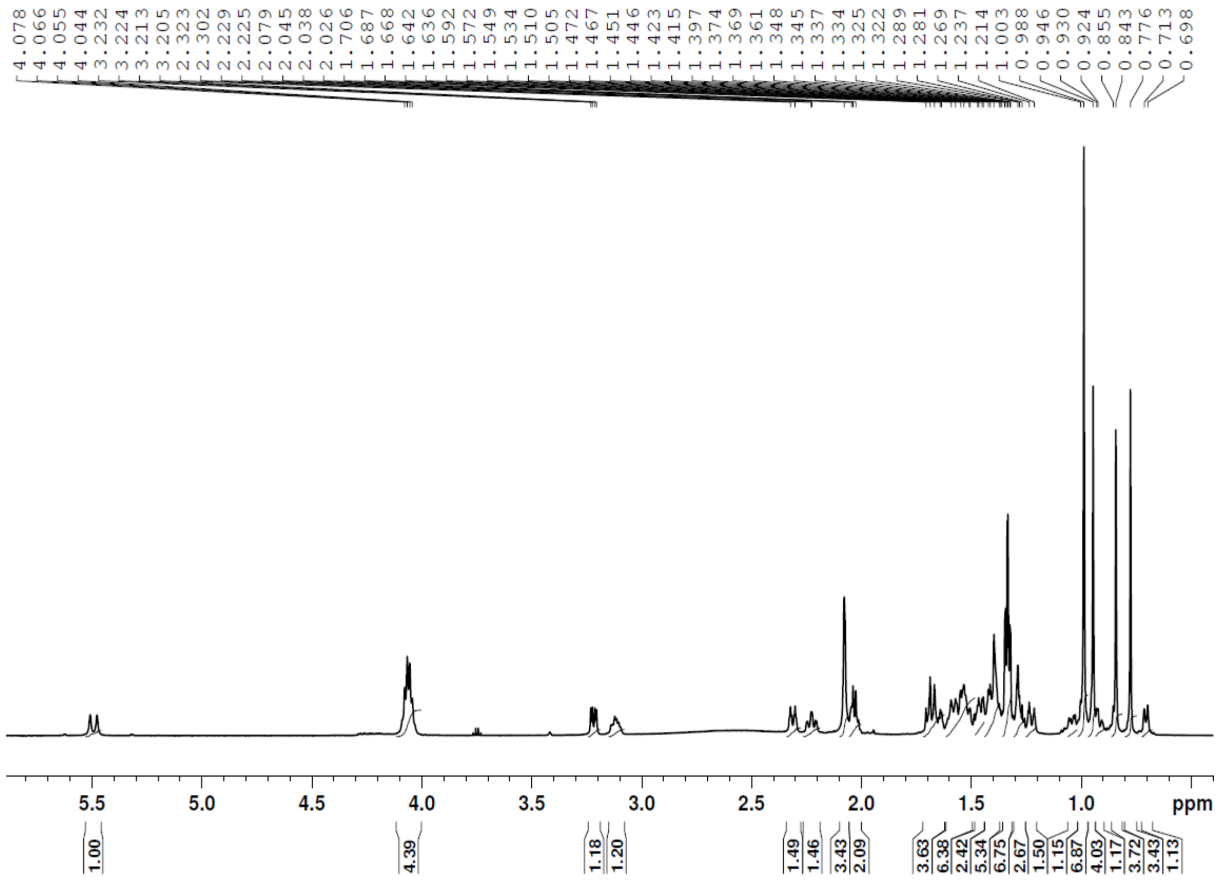

^{13}C NMR

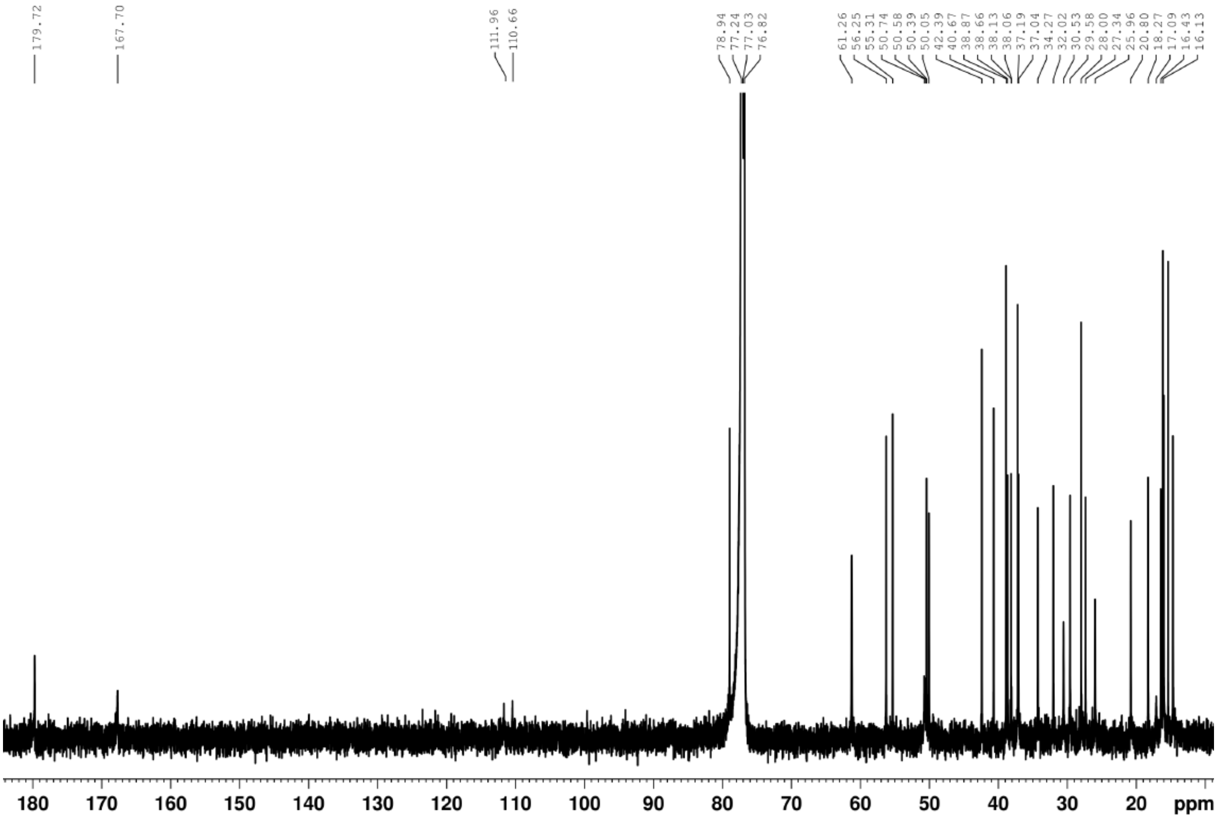

^{31}P NMR

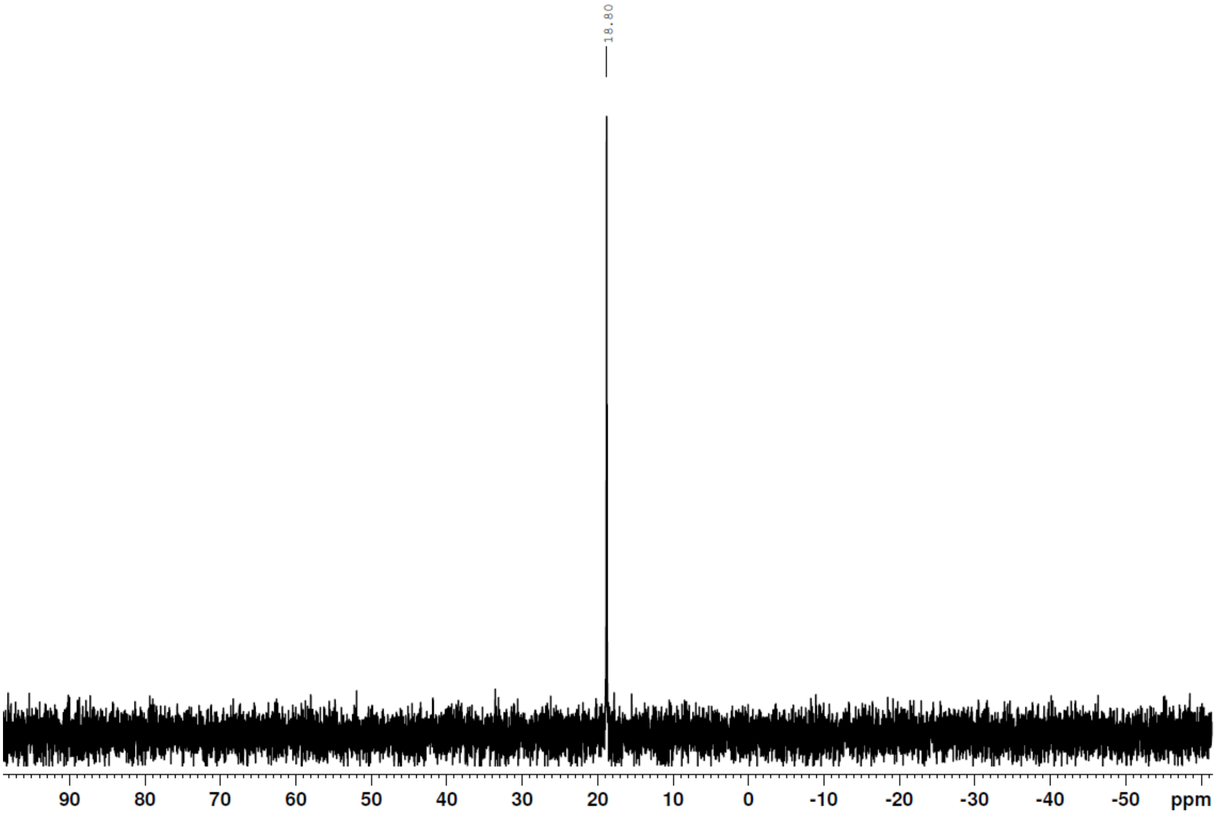

30-Diethoxyphosphorylbetulinic acid ***11***

Yield 66%; mp 266-268 °C; R_f = 0.31 (chloroform/ethanol, 15:1, v/v); IR (KBr) ν (cm^{-1}): 3431, 1681, 1236, 1024, 751; ^{1}H NMR ($CDCl_3$) δ (ppm): 5.07 (m, 1H, H29), 5.03 (m, 1H, H29), 4.13 (m, 4H, 2 x OC$H_2$$CH_3$), 3.21 (m, 1H, H3), 3.02 (m, 1H, H19), 2.60 (m, 2H, H30), 1.20 – 2.30 (m, 23 H, CH, CH_2), 1.35 (m, 6H, 2 x OCH_2CH_3), 0.99 (s, 3H, CH_3), 0.98 (s, 3H, CH_3), 0.94 (s, 3H, CH_3), 0.92 (s, 3H, CH_3), 0.77 (s, 3H, CH_3), 0.70 (m, 1H, H5); ^{13}C NMR ($CDCl_3$) δ (ppm): 180.3, 145.11, 112.9, 79.0, 62.0, 62.0, 61.9, 61.8, 56.3, 55.3, 50.6, 50.5, 46.1, 42.4, 40.7, 38.9, 38.7, 38.3, 37.2, 36.7, 34.3, 32.1, 31.3, 29.7, 28.0, 27.4, 26.5, 21.0, 18.3, 16.4, 16.1, 15.4, 14.7; ^{31}P NMR ($CDCl_3$) δ (ppm): 27.9; HR-MS (APCI) *m/z*: $C_{34}H_{56}O_6P$ $[(M–H)^-]$, Calc. 591.3815; Found 591.3827.

^{1}H NMR

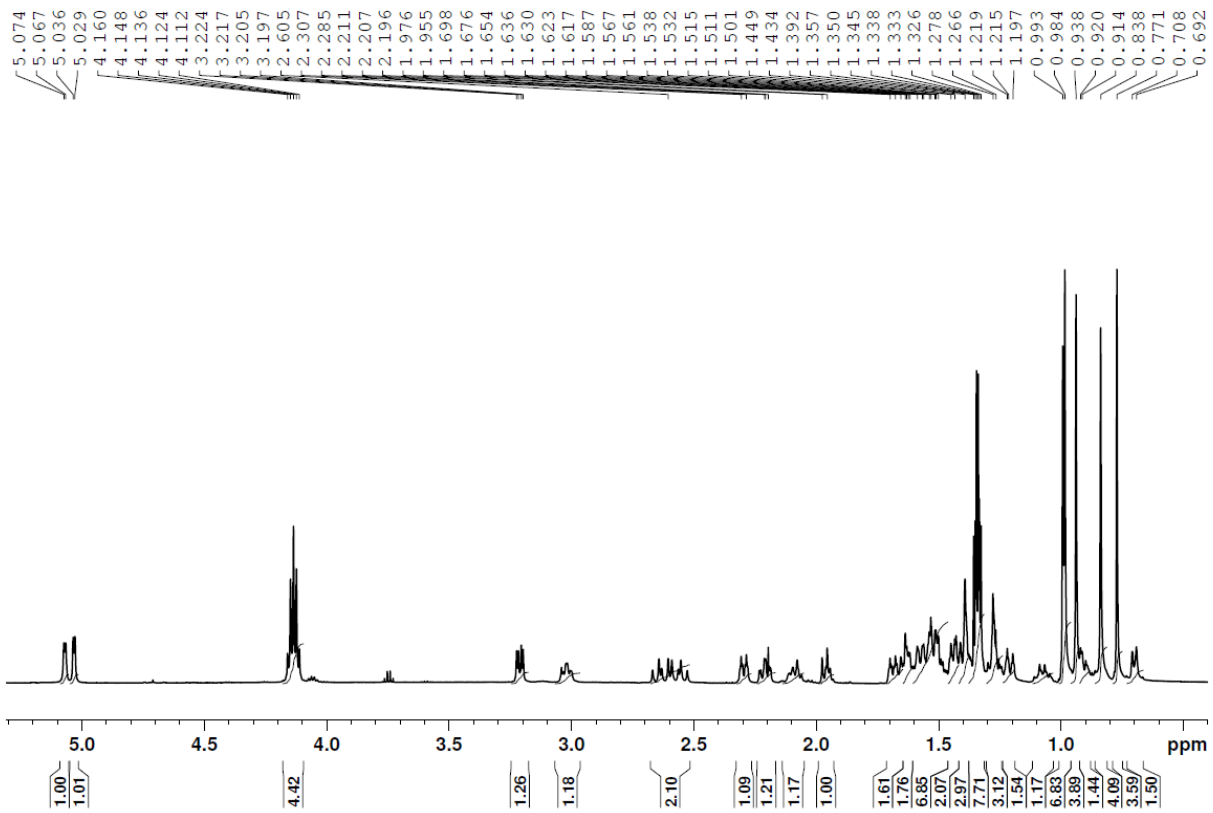

^{13}C NMR

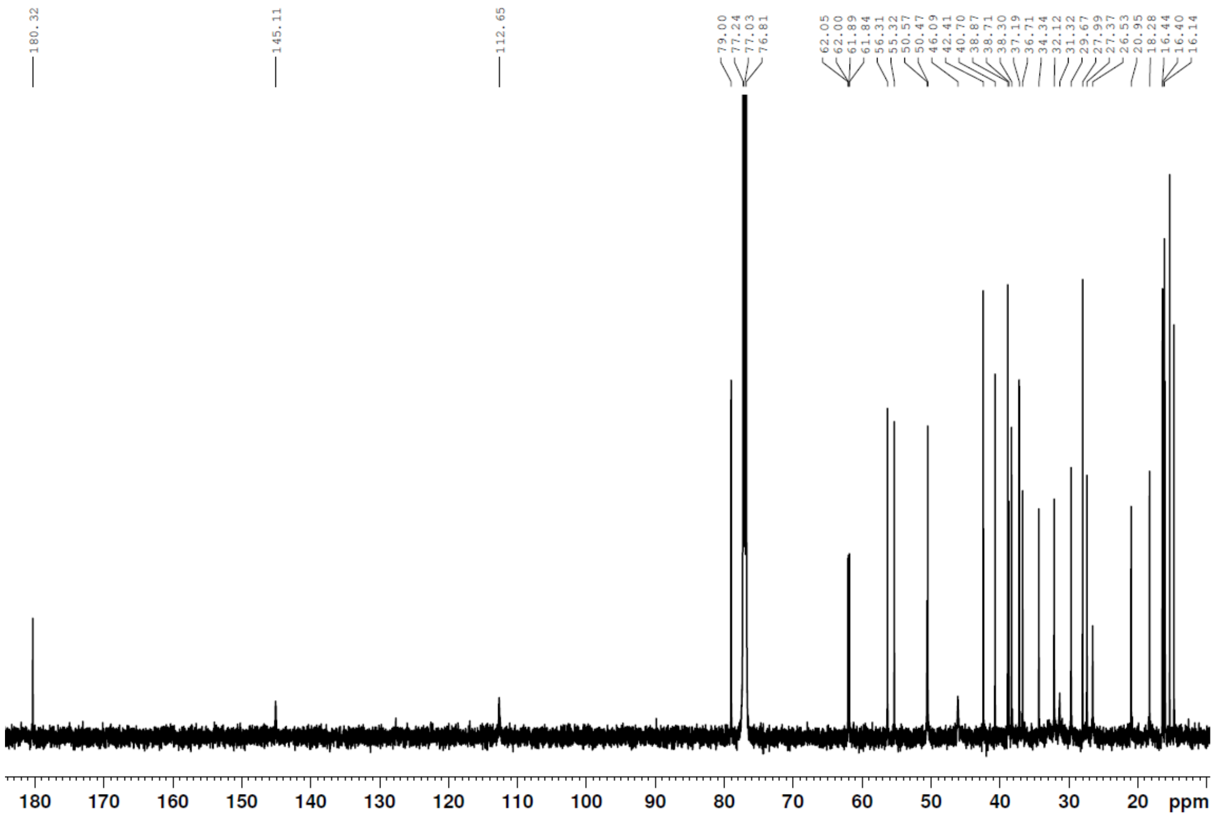

^{31}P NMR

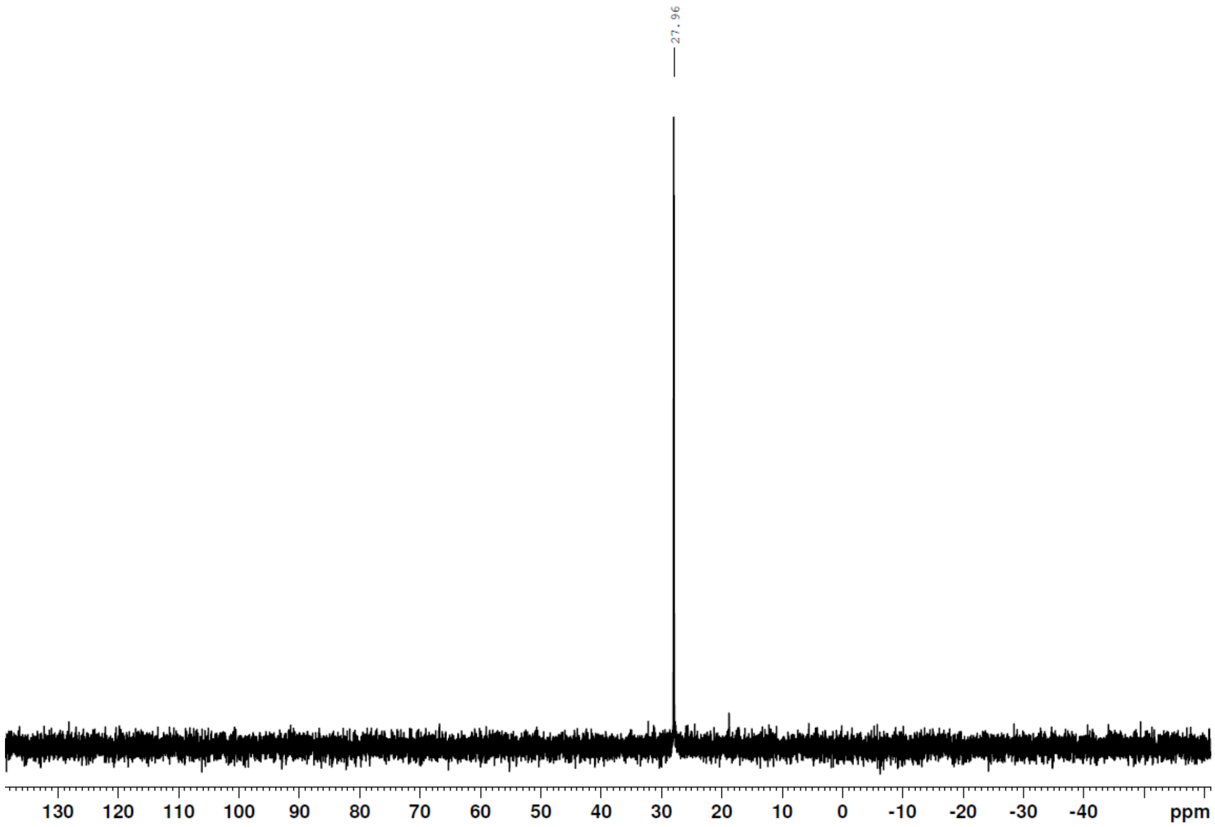

30-Diethoxyphosphoryloxy-3-O-(3’,3’-dimethylsuccinyl)betulinic acid 12a

Yield 28%; mp 110-112 °C; R_f = 0.26 (chloroform/ethanol, 15:1, v/v); IR (KBr) ν (cm^{-1}): 2947, 1732, 1705, 1240, 1031; ^1H NMR ($CDCl_3$) δ (ppm): 4.97 (m, 1H, H29), 4.92 (m, 1H, H29), 4.45 (m, 1H, H3), 4.43 (m, 2H, H30), 4.09 (m, 4H, 2 x OCH_2CH_3), 2.89 (m, 1H, H19), 2.82 (d, 1H, J = 15.6 Hz, CH), 2.38 (d, 1H, J = 15.6 Hz, CH), 0.90 – 2.60 (m, 26 H, CH, CH_2), 1.24 (m, 6H, 2 x OCH_2CH_3), 1.20 (s, 3H, CH_3), 1.17 (s, 3H, CH_3), 0.90 (s, 6H, 2 x CH_3), 0.78 (s, 3H, CH_3), 0.77 (s, 3H, CH_3), 0.72 (s, 3H, CH_3), 0.71 (m, 1H, H5); ^{13}C NMR ($CDCl_3$) δ (ppm): 183.1, 182.5, 170.6, 149.4, 110.3, 81.4, 69.3, 63.9, 63.9, 56.6, 55.0, 49.9, 49.5, 45.5, 44.1, 42.2, 40.7, 40.5, 38.3, 37.8, 37.1, 36.8, 33.8, 32.0, 32.0, 29.9, 28.5, 27.0, 26.4, 25.3, 24.2, 23.6, 21.0, 18.4, 17.4, 16.9, 16.6, 16.2, 16.2, 14.5; ^{31}P NMR ($CDCl_3$) δ (ppm): -1.02; HR-MS (APCI) *m/z*: $C_{40}H_{64}O_{10}P$ [$(M–H)^-$], Calc. 735.4237; Found 735.4229.

^1H NMR

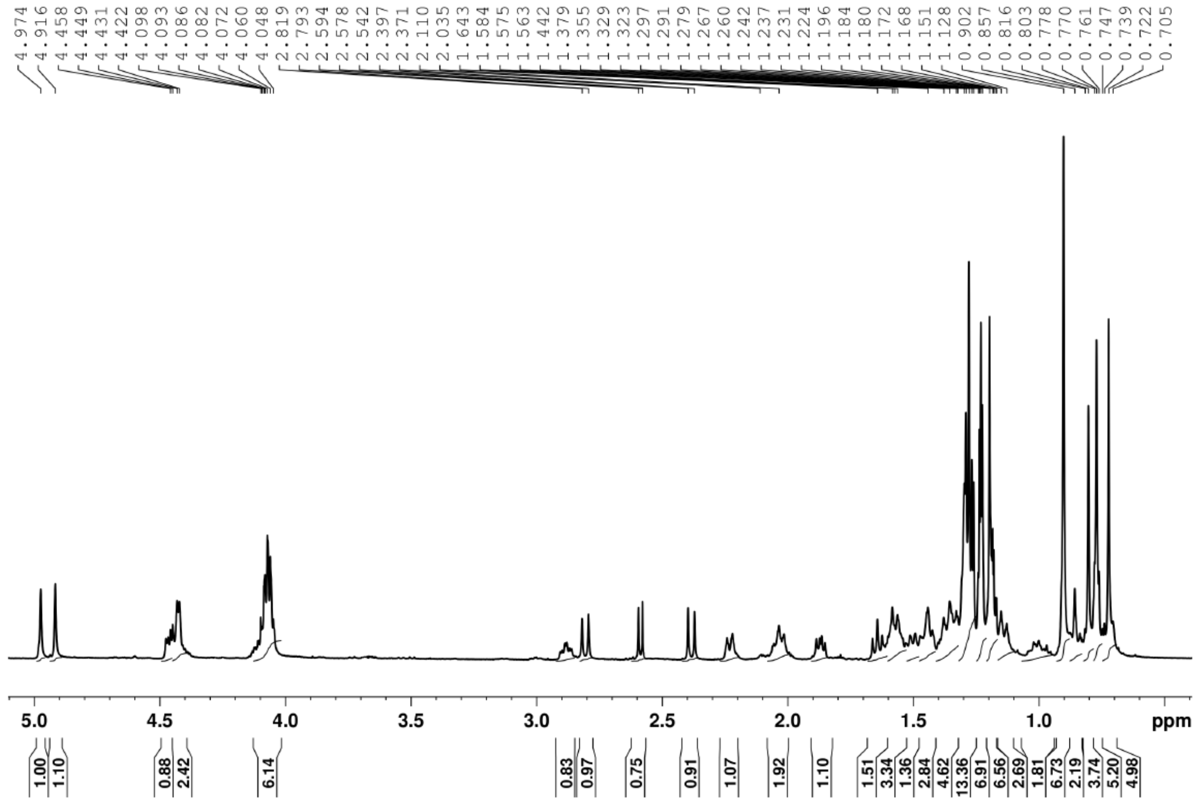

^{13}C NMR

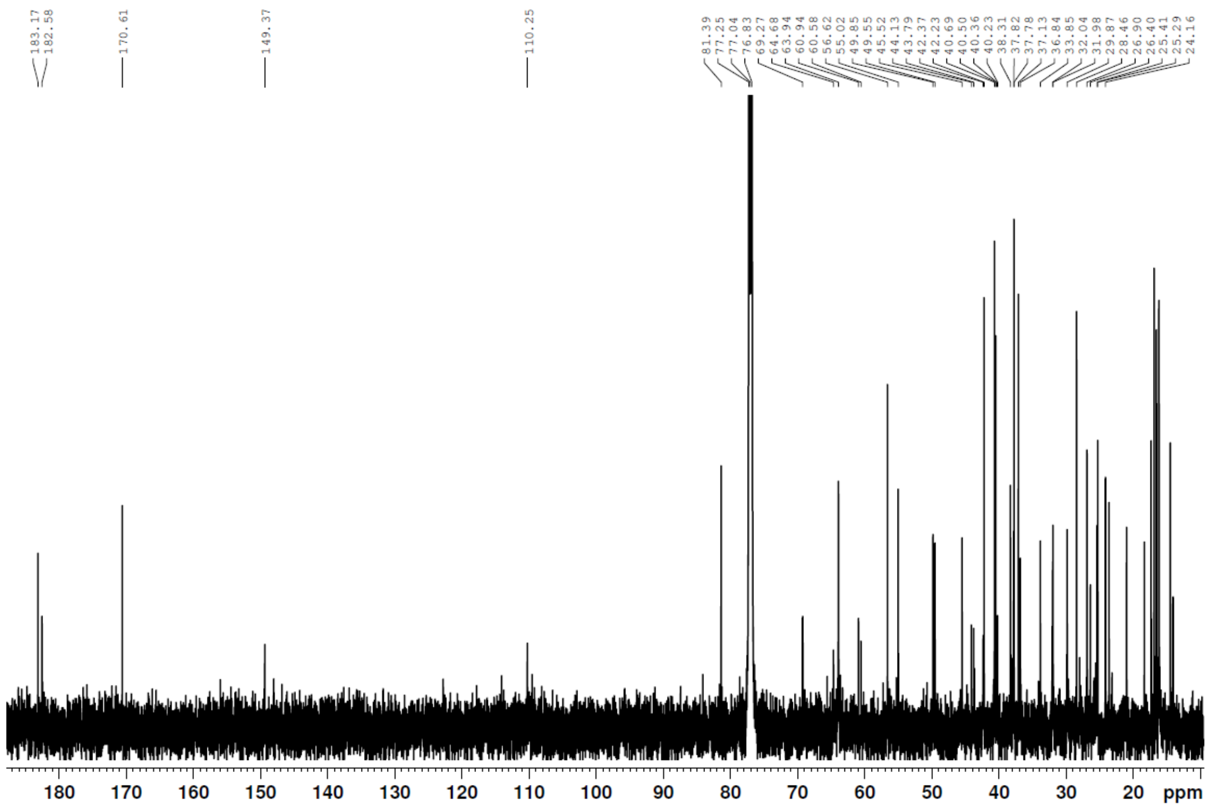

^{31}P NMR

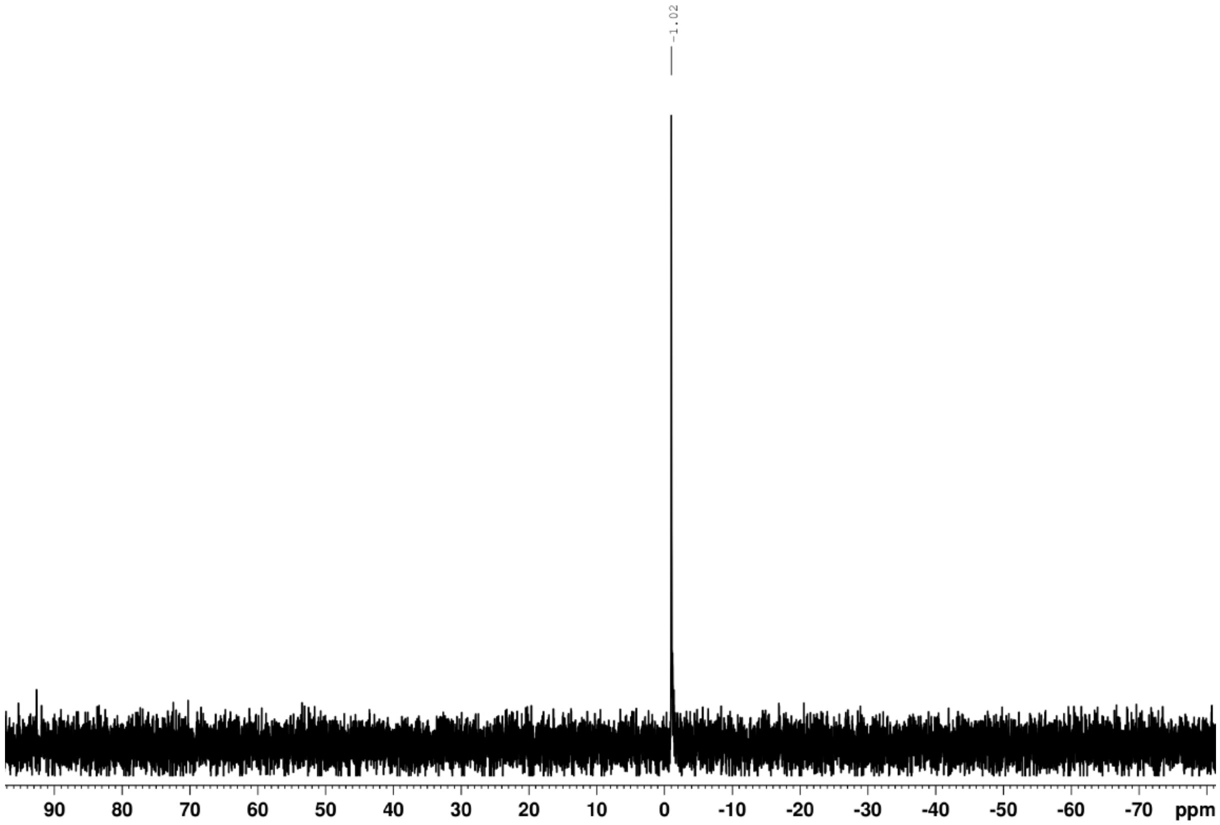

30-Diethoxyphosphoryloxy-3-O-(3,3'-dimethylglutaryl)betulonic acid 12b

Yield 34%; mp 148-150 °C; R_f = 0.28 (chloroform/ethanol, 15:1, v/v); IR (KBr) ν (cm^{-1}): 2945, 1726, 1240, 1031; ^1H NMR ($CDCl_3$) δ (ppm): 5.07 (m, 1H, H29), 5.01 (m, 1H, H29), 4.51-4.52 (m, 1H, H3 and 2H, H30), 4.16 (m, 4H, 2 x OCH_2CH_3), 2.94 (m, 1H, H19), 2.79 (d, 1H, *J* =15.6 Hz, H4'), 2.53 (d, 1H, *J* =15.6 Hz, H4'), 2.39 (d, 1H, *J* =16.2 Hz, H2'), 2.28 (d, 1H, *J* =16.2 Hz, H2'), 1.37 (m, 6H, 2 x OCH_2CH_3), 0.75 – 2.35 (m, 25 H, CH, CH_2), 1.19 (s, 3H, CH_3), 1.11 (s, 3H, CH_3), 0.99 (s, 3H, CH_3), 0.96 (s, 3H, CH_3), 0.88 (s, 3H, CH_3), 0.87 (s, 3H, CH_3), 0.82 (s, 3H, CH_3), 0.82 (m, 1H, H5); ^{13}C NMR ($CDCl_3$) δ (ppm): 182.1, 177.2, 172.1, 149.4, 110.1, 81.0, 69.1, 63.9, 63.9, 56.4, 55.1, 49.9, 49.8, 45.0, 44.0, 42.4, 42.2, 40.6, 38.1, 37.9, 37.8, 37.2, 36.6, 33.9, 32.4, 32.0, 29.8, 28.8, 28.5, 28.4, 26.4, 23.9, 21.0, 18.3, 16.9, 16.8, 16.3, 16.2, 16.2, 14.5; ^{31}P NMR ($CDCl_3$) δ (ppm): -0.94; HR-MS (APCI) *m/z*: $C_{41}H_{66}O_{10}P$ [$(M–H)^-$], Calc. 749.4394; Found 749.4385.

^1H NMR

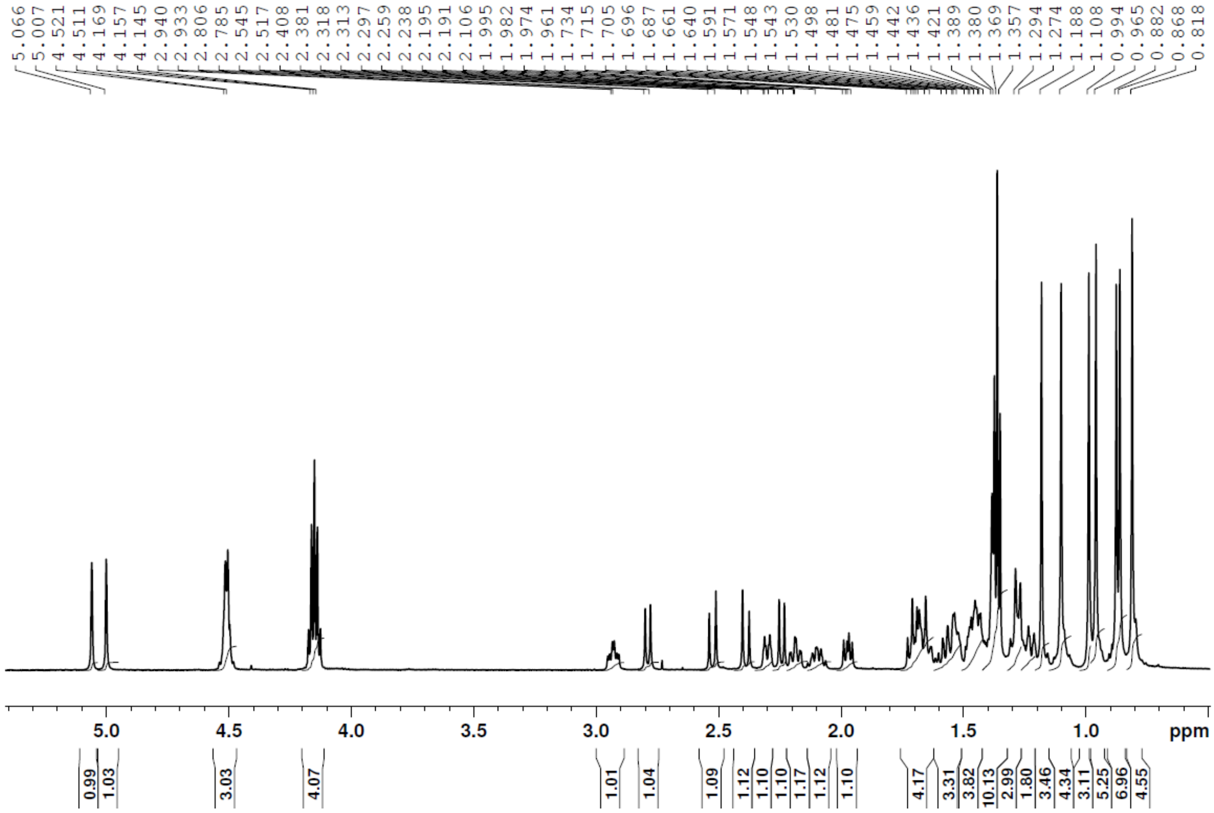

^{13}C NMR

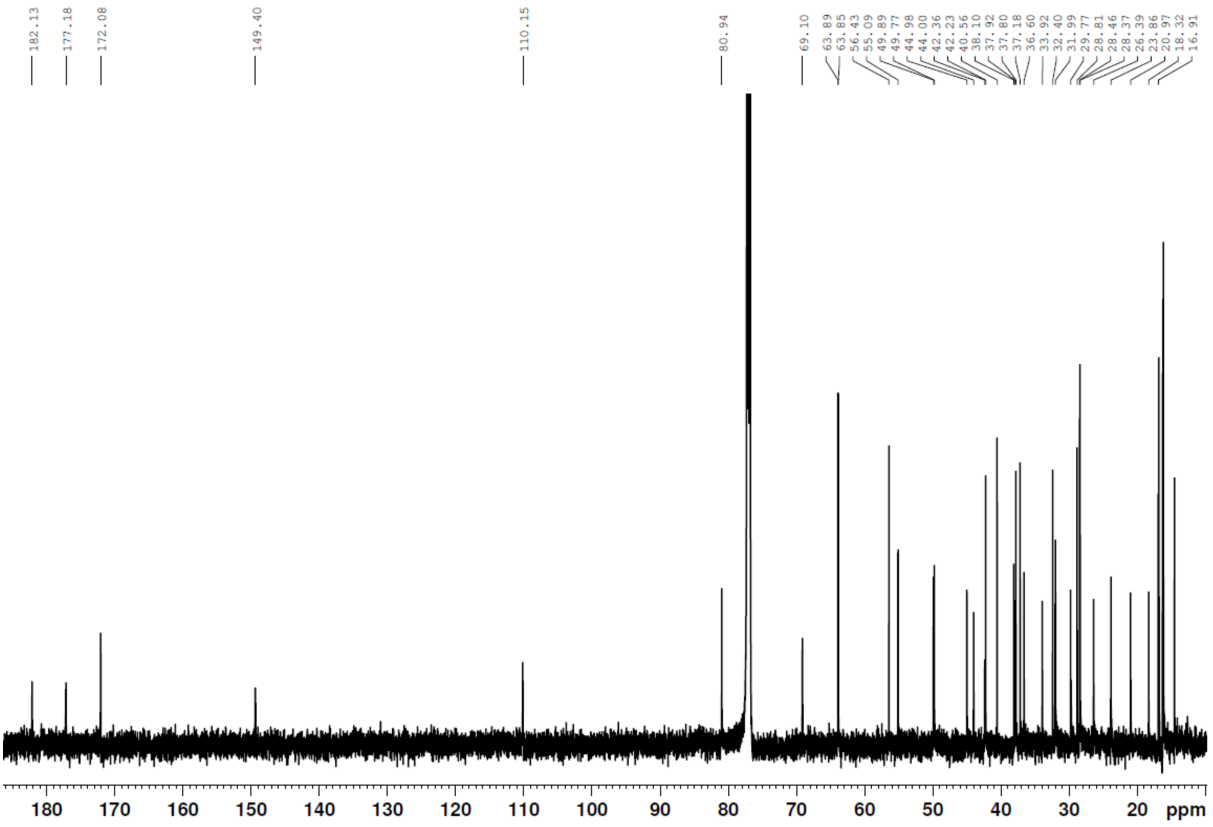

^{31}P NMR

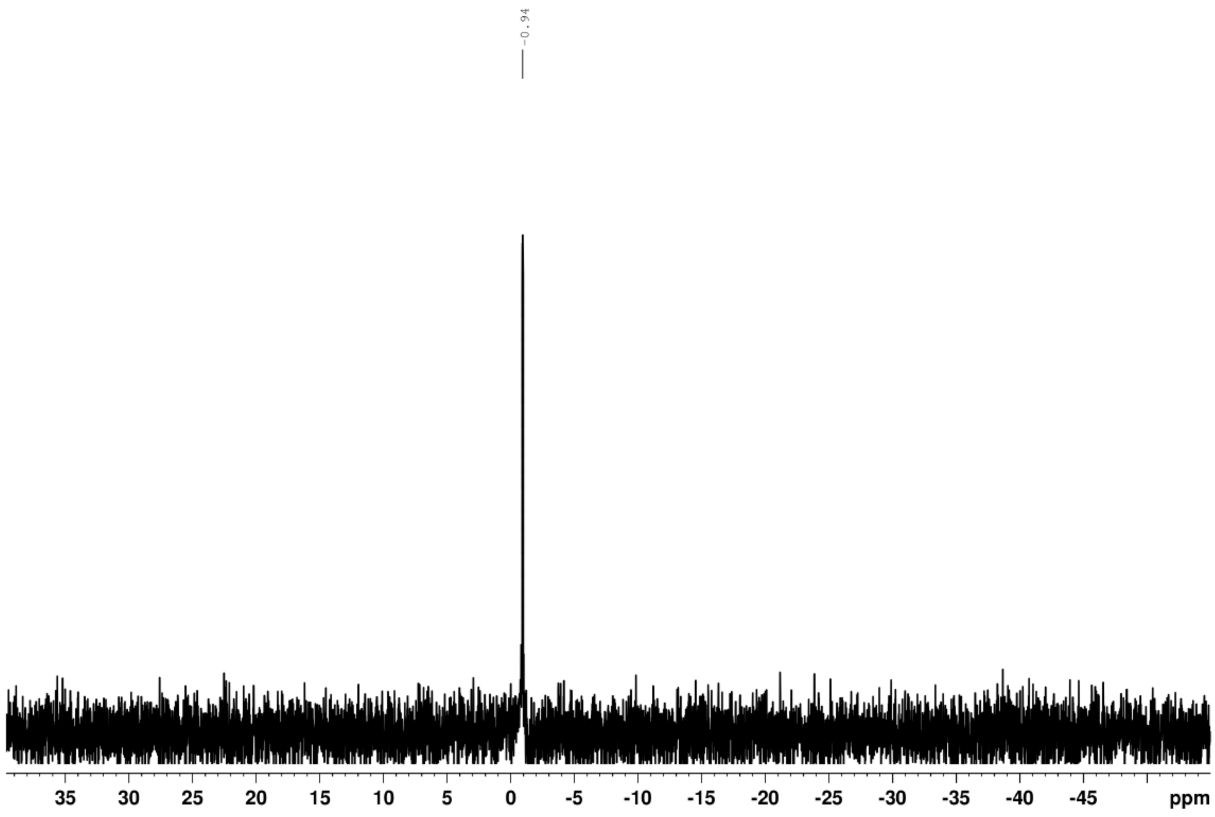

30-Diethoxyphosphoryloxy-3-O-(4’,4’-dimethylglutaryl)betulonic acid 12c

Yield 32%; mp 136-138 °C; R_f = 0.27 (chloroform/ethanol, 15:1, v/v); IR (KBr) ν (cm^{-1}): 3213, 2998, 1732, 1699, 1242, 1031; ^{1}H NMR ($CDCl_3$) δ (ppm): 5.07 (m, 1H, H29), 5.01 (m, 1H, H29), 4.52 (d, 2H, H30), 4.49 (m, 1H, H3), 4.16 (m, 4H, 2 x OCH_2CH$_3$), 2.95 (m, 1H, H19), 1.37 (m, 6H, 2 x OCH$_2$CH_3), 0.75 – 2.35 (m, 27 H, CH, CH$_2$), 1.24 (s, 3H, CH$_3$), 1.22 (s, 3H, CH$_3$), 1.00 (s, 3H, CH$_3$), 0.92 (s, 3H, CH$_3$), 0.87 (s, 3H, CH$_3$), 0.86 (s, 3H, CH$_3$), 0.85 (s, 3H, CH$_3$), 0.81 (m, 1H, H5); ^{13}C NMR ($CDCl_3$) δ (ppm): 183.4, 182.0, 172.9, 131.3, 114.3, 80.8, 69.1, 63.9, 63.9, 60.5, 56.4, 55.1, 50.0, 42.3, 42.2, 41.6, 40.6, 38.4, 38.0, 37.9, 37.1, 35.0, 34.9, 34.0, 32.0, 30.8, 30.2, 29.8, 28.2, 26.5, 24.9, 24.5, 24.2, 23.7, 20.9, 18.2, 17.0, 16.3, 16.2, 16.2, 14.6; ^{31}P NMR ($CDCl_3$) δ (ppm): -0.98; HR-MS (APCI) *m/z*: $C_{41}H_{66}O_{10}P$ [(M–H)$^-$], Calc. 749.4394; Found 749.4386.

^{1}H NMR

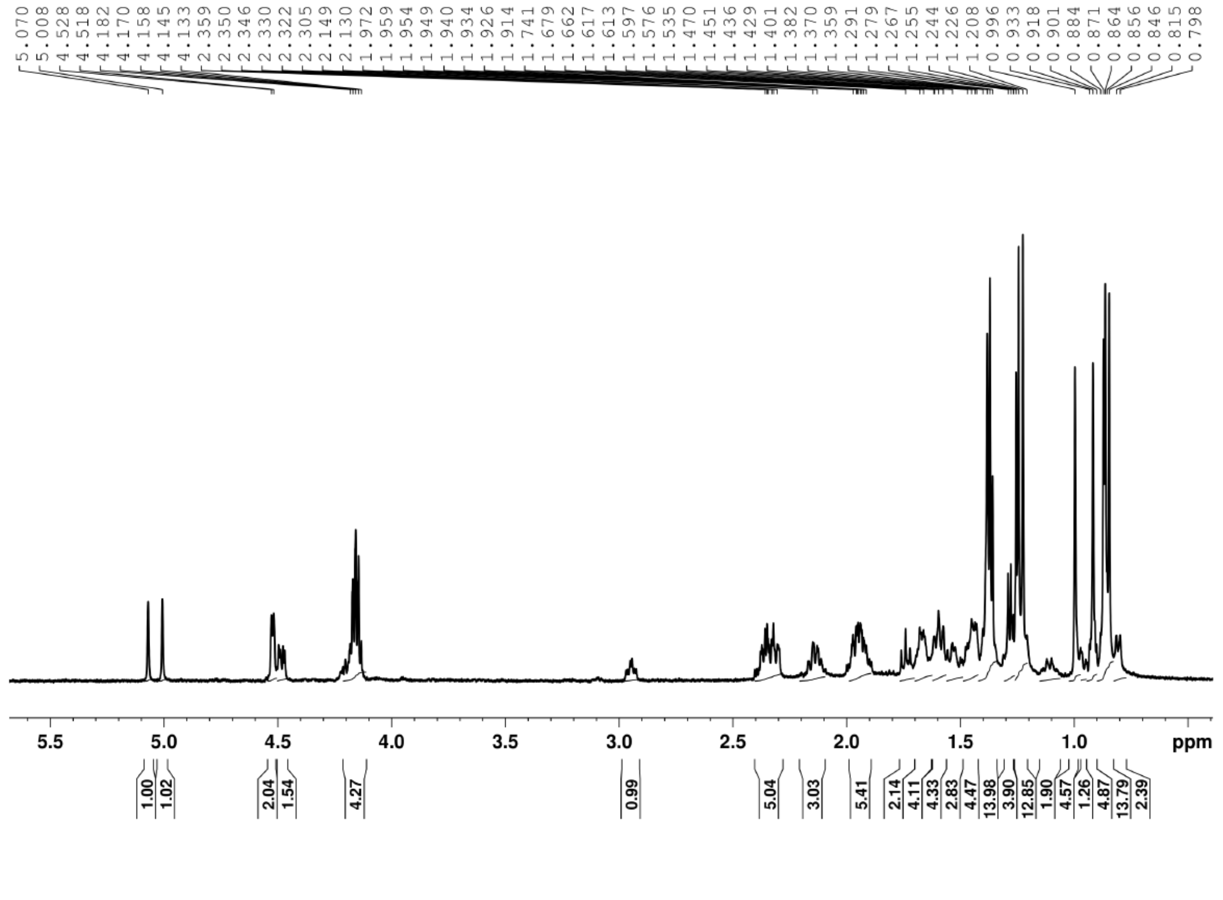

^{13}C NMR

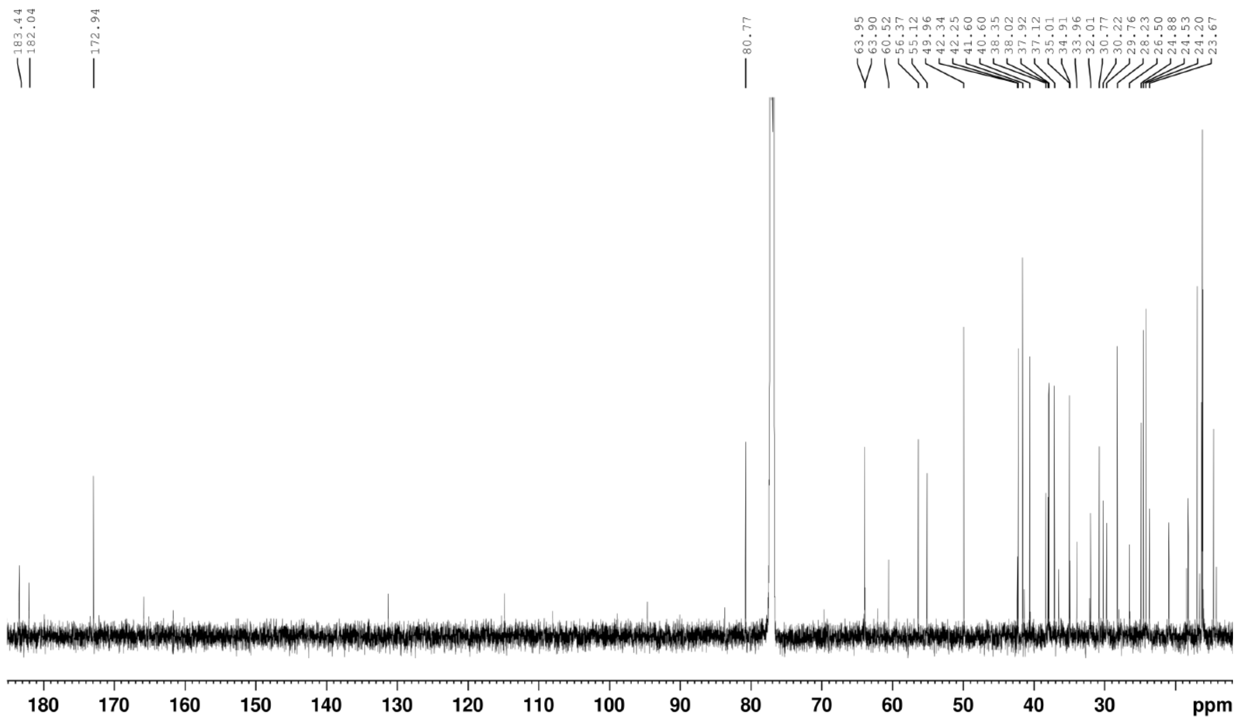

^{31}P NMR

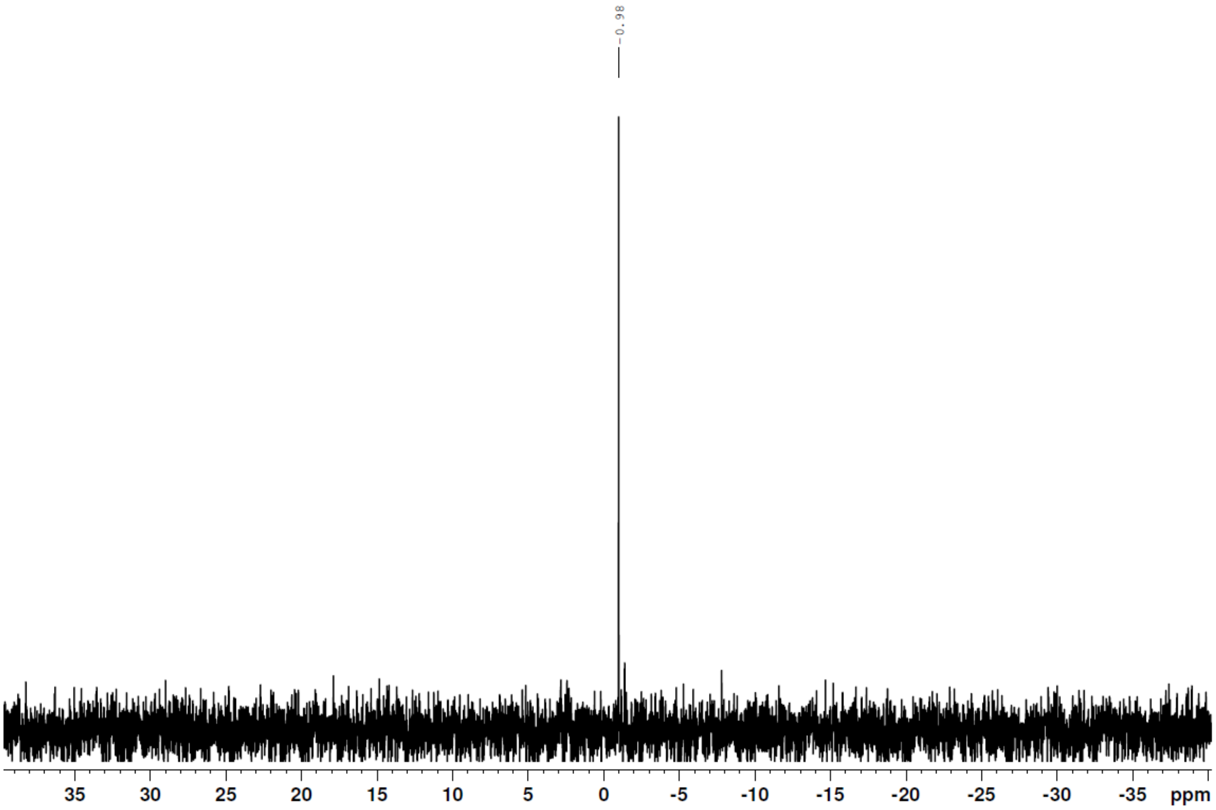

29-Diethoxyphosphoryl-3-O-(3’,3’-dimethylsuccinyl)betulonic acid 13a

Yield 22%; mp 150-154 °C; R_f = 0.13 (chloroform/ethanol, 15:1, v/v); IR (KBr) ν (cm^{-1}): 3550, 2945, 1728, 1219, 1024, 752; ^1H NMR ($CDCl_3$) δ (ppm): 5.39 (m, 1H, H29), 4.47 (m, 1H, H3), 3.97 (m, 4H, 2 x OCH_2CH_3), 3.04 (m, 1H, H19), 2.80 (d, 1H, *J* = 15.6 Hz, CH), 2.40 (d, 1H, *J* = 15.6 Hz, CH), 1.20 – 2.30 (m, 24 H, CH, CH_2), 1.98 (br. s, 3H, CH_3), 1.25 (m, 6H, 2 x OCH_2CH_3), 1.23 (s, 3H, CH_3), 1.20 (s, 3H, CH_3), 0.89 (s, 3H, CH_3), 0.87 (s, 3H, CH_3), 0.79 (s, 3H, CH_3), 0.77 (s, 3H, CH_3), 0.73 (s, 3H, CH_3); ^{13}C NMR ($CDCl_3$) δ (ppm): 183.0, 182.0, 170.5, 111.3, 81.3, 61.3, 56.6, 55.1, 50.1, 49.6, 49.5, 45.5, 42.3, 42.2, 40.7, 40.5, 38.2, 37.9, 37.2, 37.1, 33.9, 33.8, 30.9, 30.6, 29.8, 29.7, 28.4, 28.0, 26.8, 26.8, 25.7, 24.3, 23.6, 18.4, 17.3, 16.9, 16.5, 16.4, 16.3, 14.7; ^{31}P NMR ($CDCl_3$) δ (ppm): 18.6; HR-MS (APCI) *m/z*: $C_{40}H_{64}O_9P$ [$(M–H)^-$], Calc. 719.4288; Found 719.4275.

^1H NMR

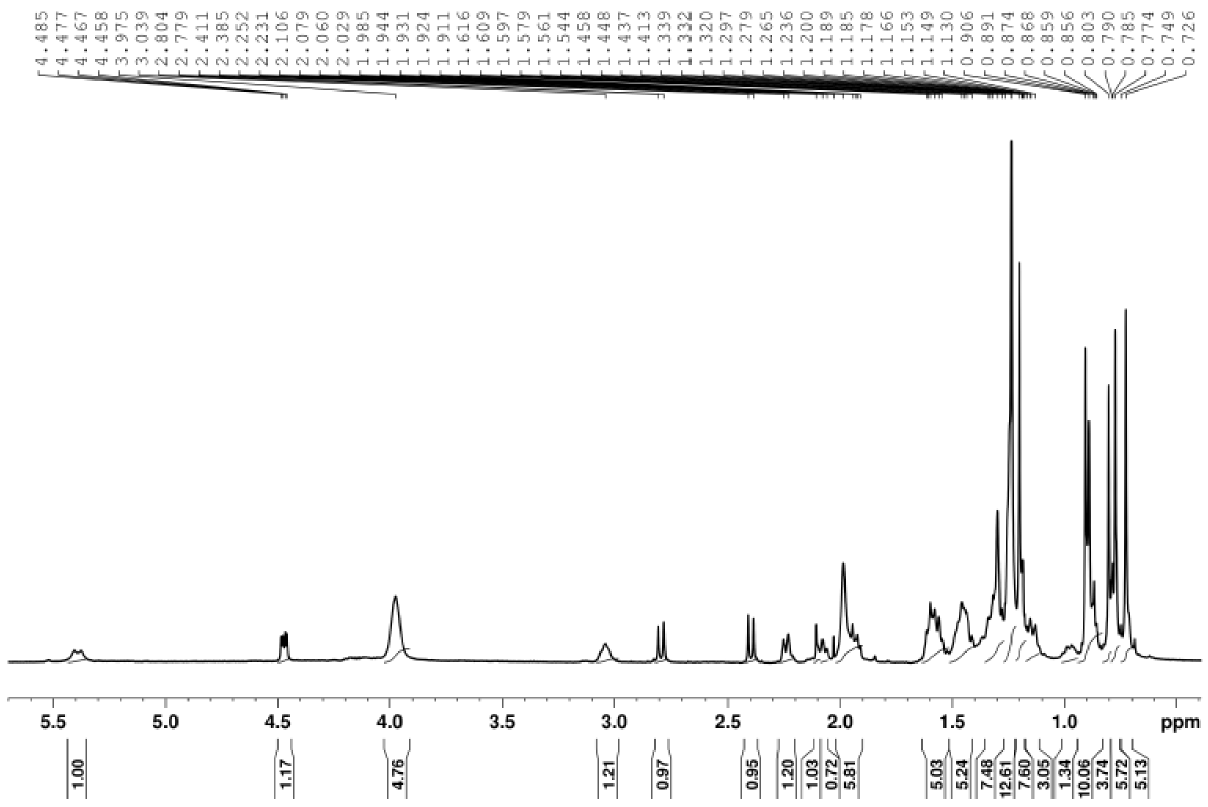

^{13}C NMR

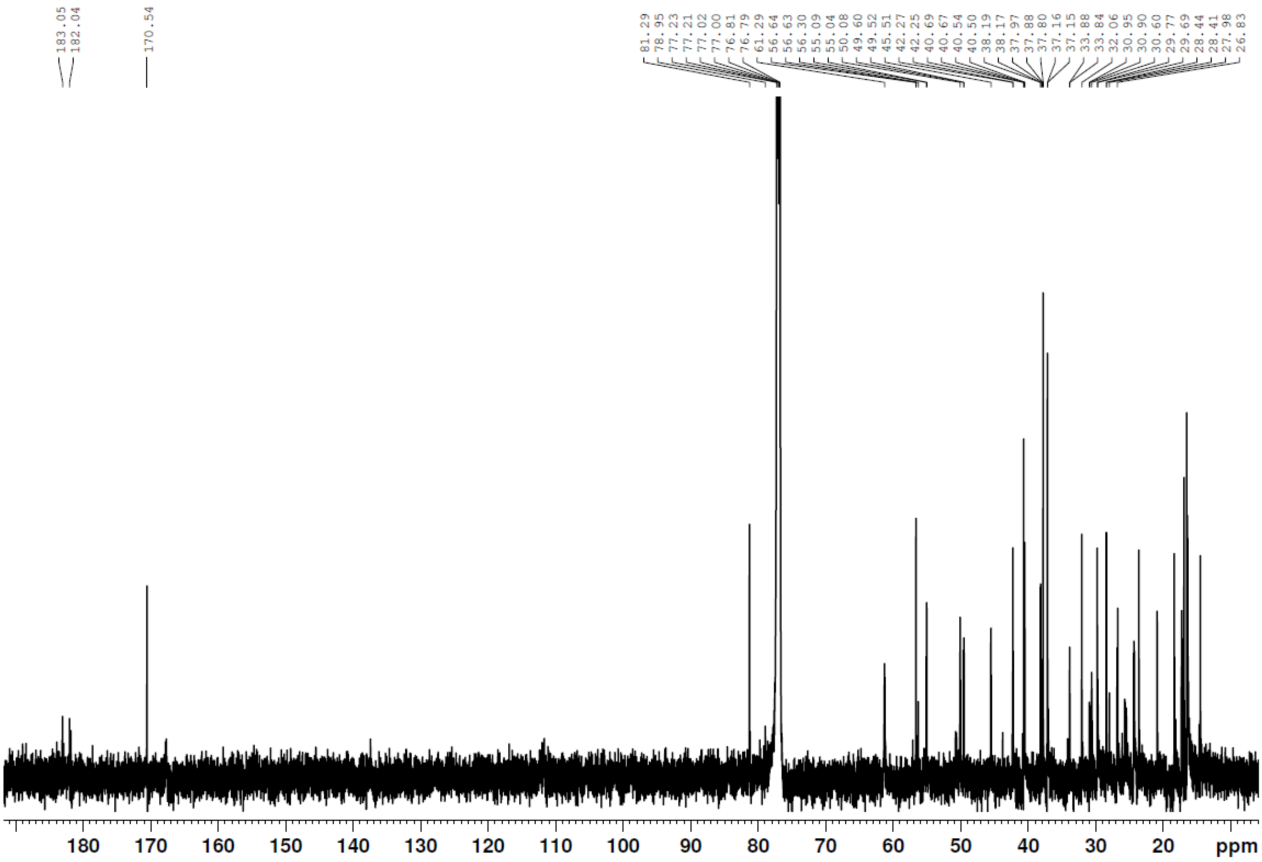

^{31}P NMR

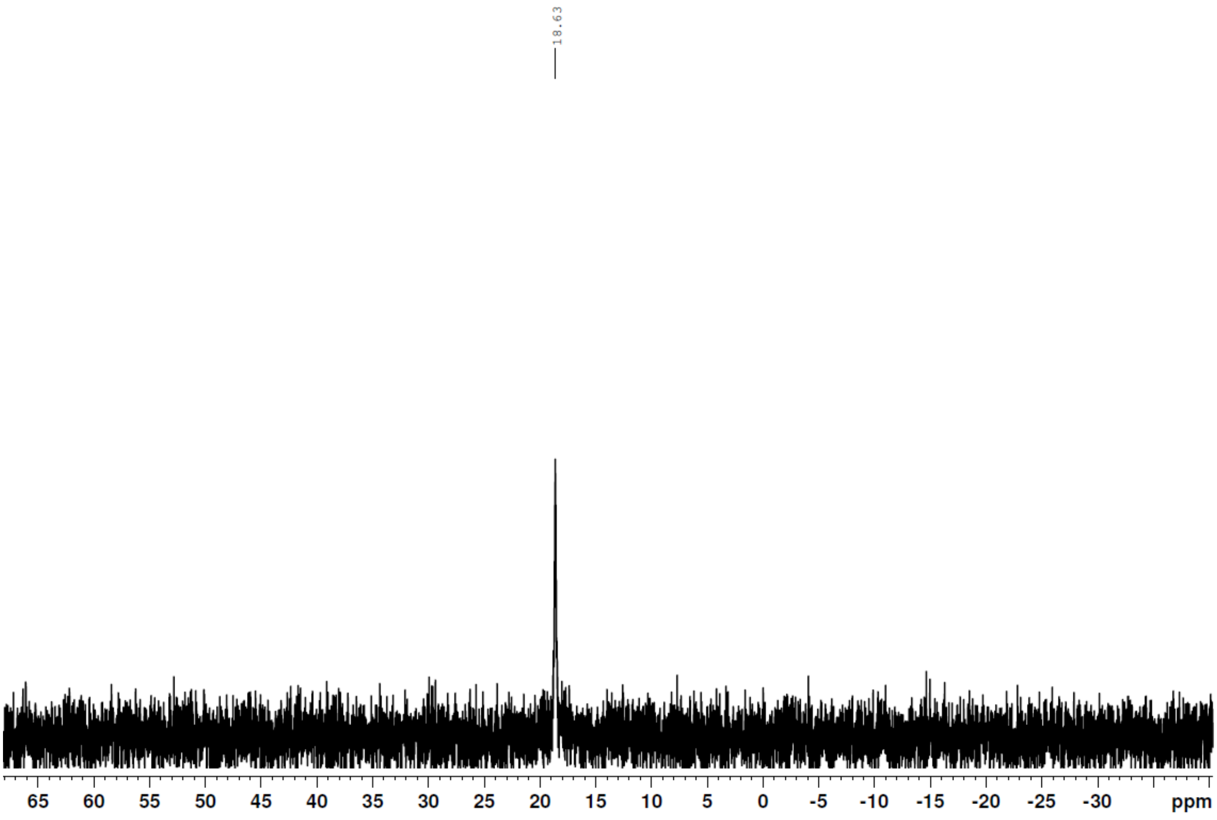

29-Diethoxyphosphoryl-3-O-(3',3'-dimethylglutaryl)betulinic acid 13b

Yield 41%; mp 131-133 °C; R_f = 0.12 (chloroform/ethanol, 15:1, v/v); IR (KBr) ν (cm^{-1}): 2945, 1716, 1220, 1028, 754; ^1H NMR ($CDCl_3$) δ (ppm): 5.39 (d, 1H, $J_{H\text{-}P}$ = 19.2 Hz, H29), 4.43 (m, 1H, H3), 3.98 (m, 4H, 2 x OCH_2CH$_3$), 3.00 (m, 1H, H19), 2.66 (d, 1H, J =16.2 Hz, H4'), 2.43 (d, 1H, J =16.2 Hz, H4'), 2.32 (d, 1H, J =17.4 Hz, H2'), 2.28 (d, 1H, J =17.4 Hz, H2'), 0.90 – 2.50 (m, 26 H, CH, CH_2); 1.98 (s, 3H, CH_3), 1.25 (m, 6H, 2 x OCH_2CH_3), 1.10 (s, 3H, CH_3), 1.03 (s, 3H, CH_3), 0.89 (s, 3H, CH_3), 0.87 (s, 3H, CH_3), 0.79 (s, 3H, CH_3), 0.78 (s, 3H, CH_3), 0.73 (s, 3H, CH_3), 0.72 (m, 1H, H5); ^{13}C NMR ($CDCl_3$) δ (ppm): 181.9, 177.1, 172.0, 80.9, 68.3, 61.3, 60.6, 58.5, 56.4, 55.1, 50.0, 49.8, 45.1, 45.0, 44.2, 43.8, 42.3, 40.5, 37.9, 37.8, 37.1, 37.0, 33.9, 32.8, 32.4, 32.0, 31.0, 30.5, 29.6, 25.7, 23.8, 20.8, 18.2, 16.4, 16.2, 16.1, 16.0,14.6; ^{31}P NMR ($CDCl_3$) δ (ppm): 18.7; HR-MS (APCI) *m/z*: $C_{41}H_{66}O_9P$ [(M–H)$^-$], Calc. 733.4445; Found 733.4439.

^1H NMR

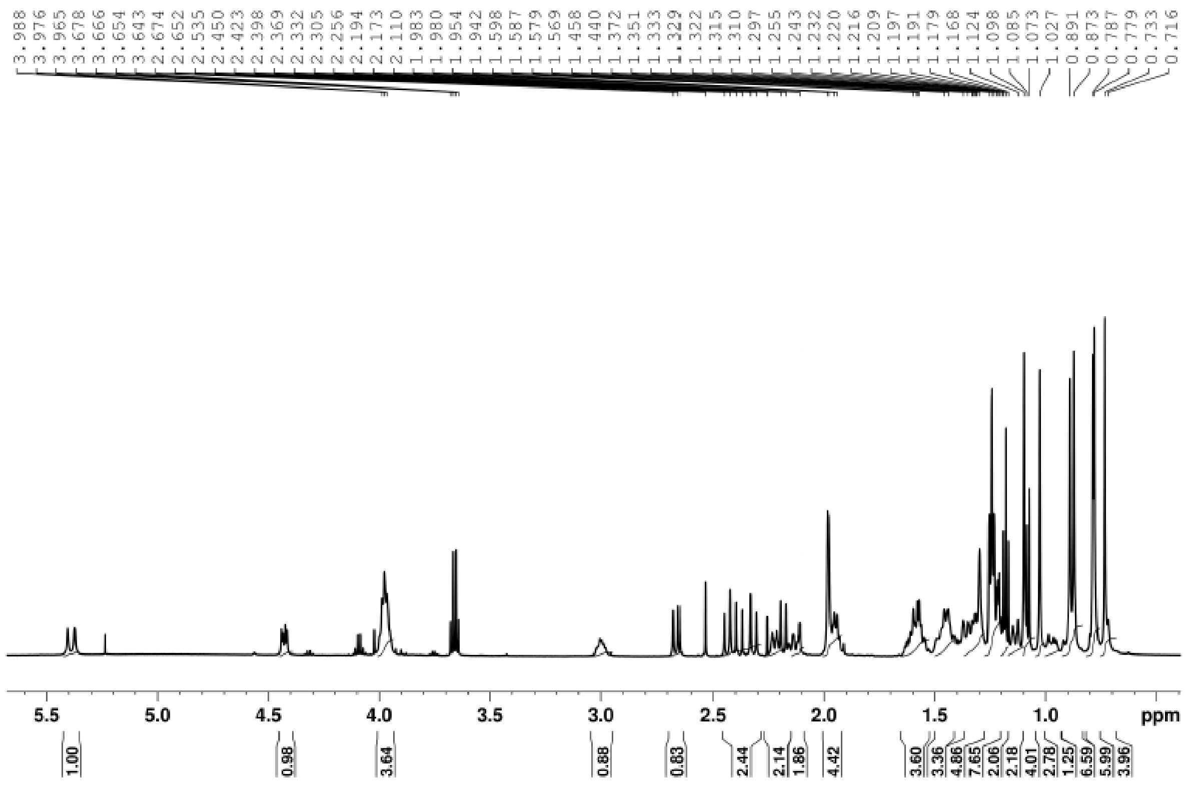

^{13}C NMR

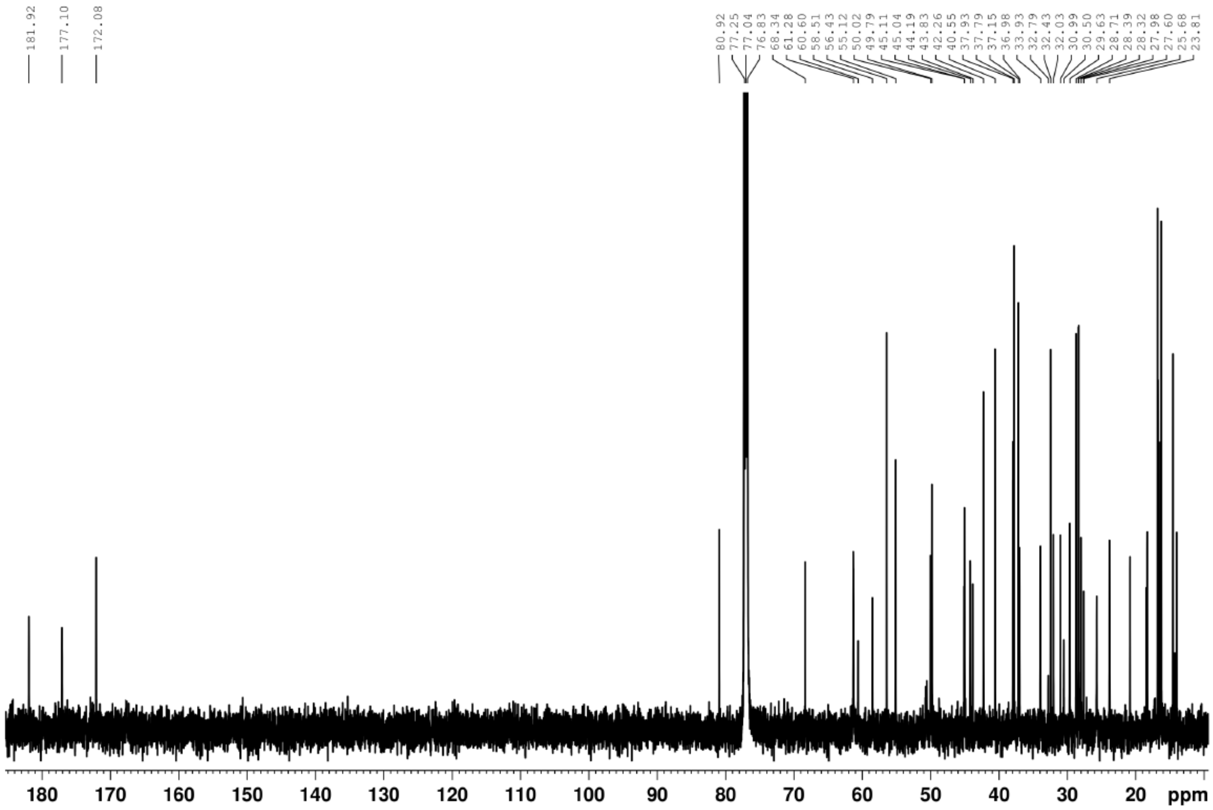

^{31}P NMR

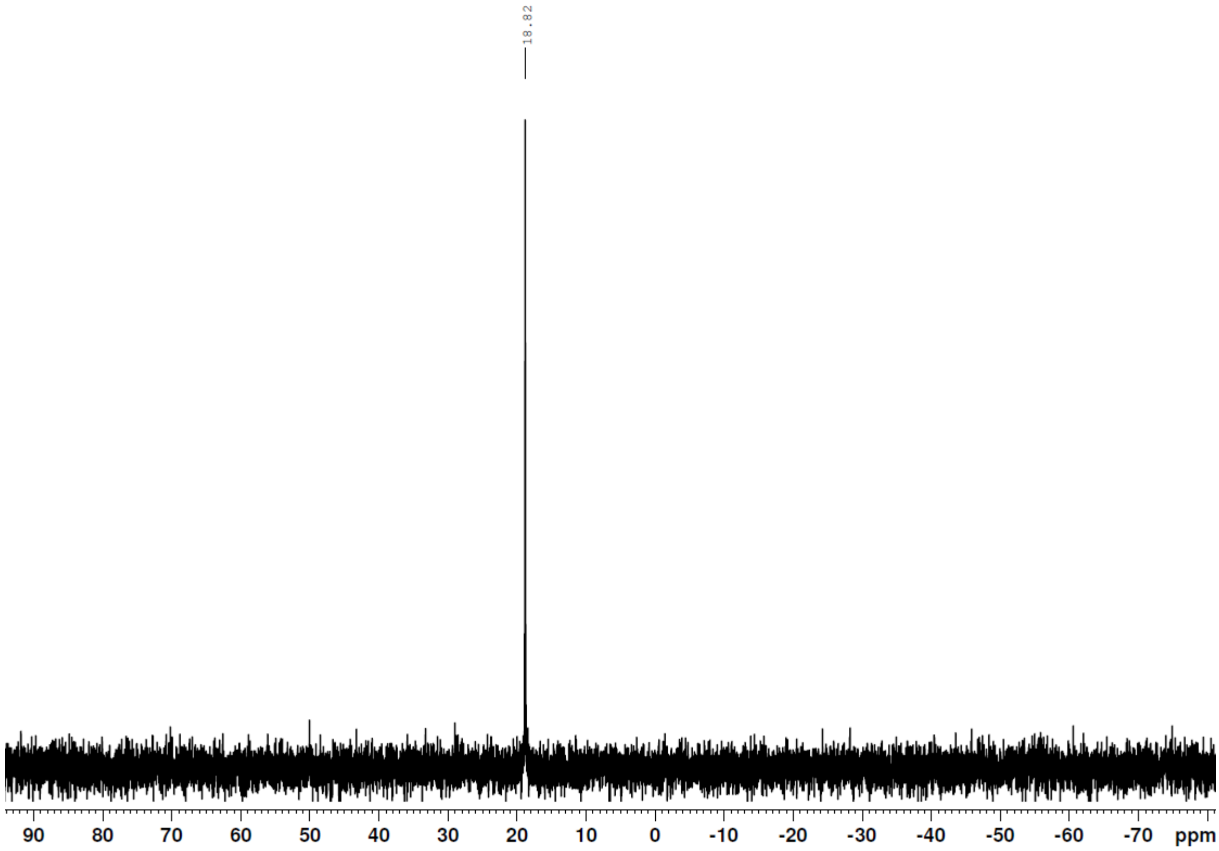

29-Diethoxyphosphoryl-3-O-(4',4'-dimethylglutaryl)betulinic acid 13c

Yield 32%; mp 141-146 °C; R_f = 0.30 (chloroform/ethanol, 15:1, v/v); IR (KBr) ν (cm^{-1}): 3647, 2945, 1716, 1220, 1028, 754; ^{1}H NMR ($CDCl_3$) δ (ppm): 5.48 (d, 1H, $J_{H\text{-}P}$ = 18.6 Hz, H29), 4.49 (m, 1H, H3), 4.06 (m, 4H, 2 x OCH_2CH$_3$), 3.10 (m, 1H, H19), 2.07 (s, 3H, CH_3), 1.20 – 2.50 (m, 27 H, CH, CH_2), 1.33 (m, 6H, 2 x OCH$_2$CH_3), 1.27 (s, 3H, CH_3), 1.23 (s, 3H, CH_3), 0.98 (s, 3H, CH_3), 0.92 (s, 3H, CH_3), 0.86 (s, 6H, 2x CH_3), 0.84 (s, 3H, CH_3), 0.70 (m, 1H, H5); ^{13}C NMR ($CDCl_3$) δ (ppm): 183.3, 181.8, 173.0, 80.8, 61.4, 56.4, 55.3, 50.1, 42.3, 41.6, 41.5, 41.2, 40.7, 38.7, 38.2, 37.9, 37.1, 37.0, 35.0, 34.1, 33.9, 32.0, 30.7, 30.0, 29.7, 29.6, 28.1, 25.9, 25.0, 24.8, 24.5, 23.6, 20.8, 18.2, 16.4, 16.2, 16.1, 16.0, 14.6; ^{31}P NMR ($CDCl_3$) δ (ppm): 18.7; HR-MS (APCI) *m/z*: $C_{41}H_{66}O_9P$ [$(M\text{–}H)^-$], Calc. 733.4445; Found 733.4451.

^{1}H NMR

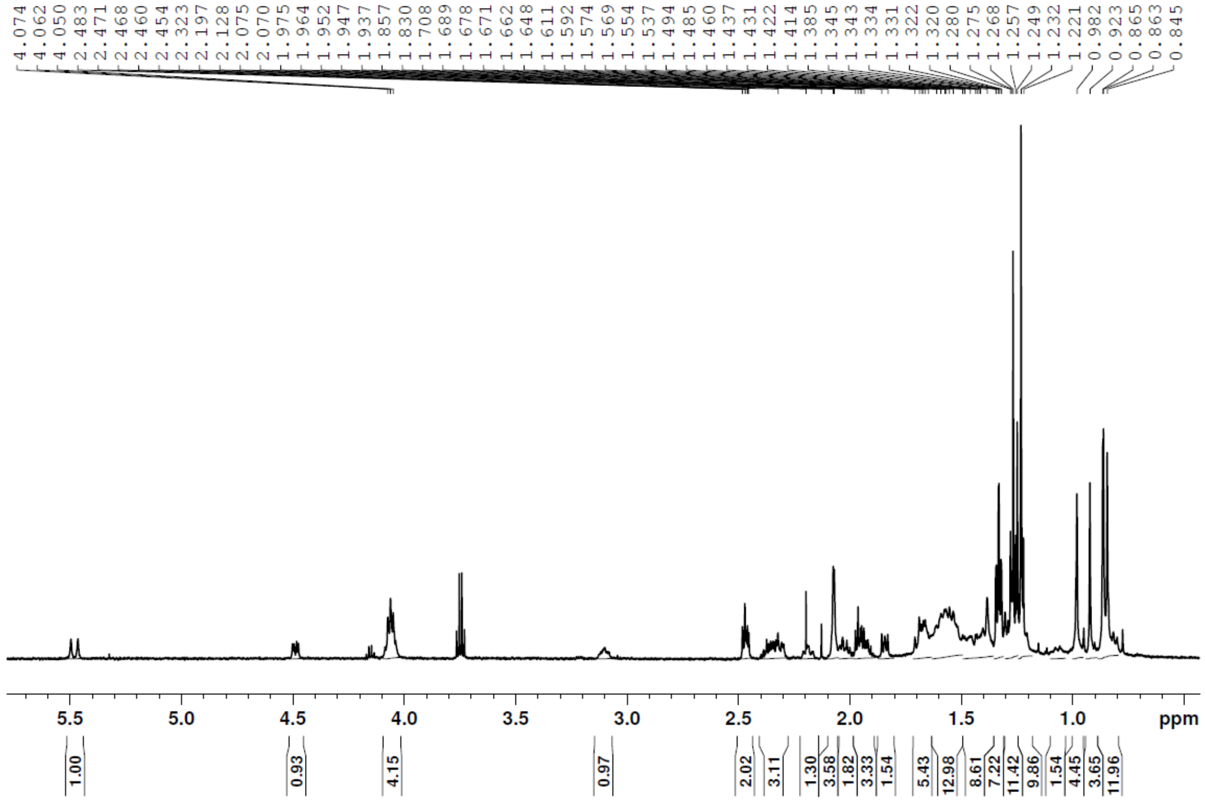

^{13}C NMR

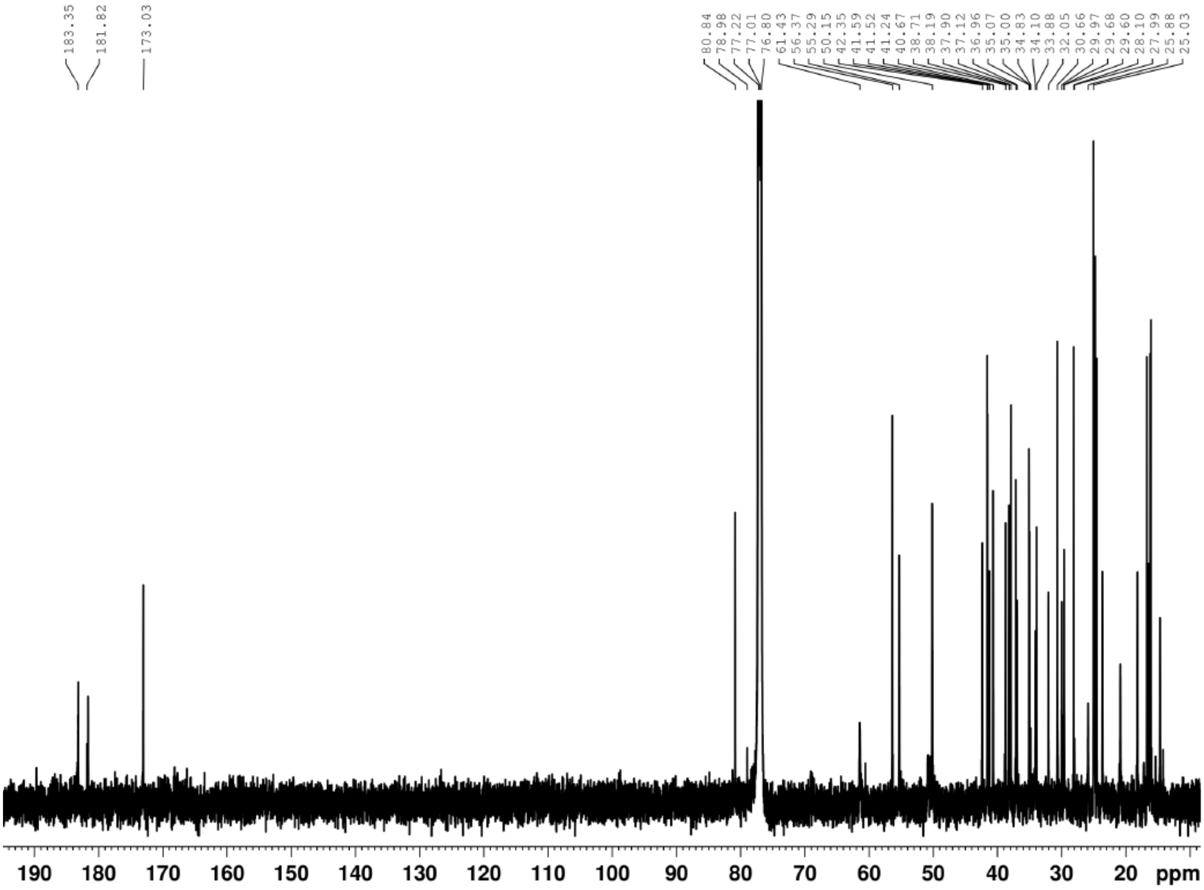

^{31}P NMR

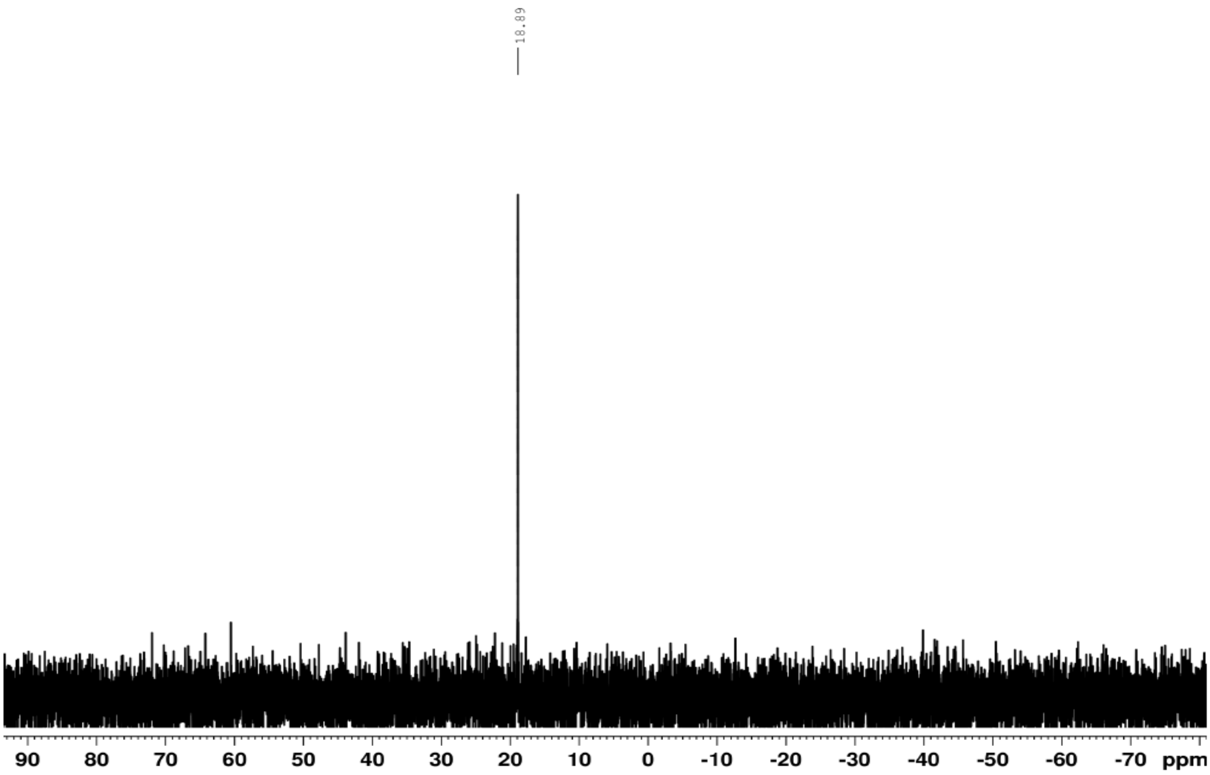

30-Diethoxyphosphoryl-3-O-(3',3'-dimethylsuccinyl)betulinic acid 14a

Yield 20%; mp 122-126 °C; R_f = 0.21 (chloroform/ethanol, 15:1, v/v); IR (KBr) ν (cm^{-1}): 3500, 2945, 1705, 1219, 1026, 752; ^{1}H NMR ($CDCl_3$) δ (ppm): 5.07 (m, 1H, H29), 5.03 (m, 1H, H29), 4.55 (m, 1H, H3), 4.13 (m, 4H, 2 x OC*H*$_2$CH$_3$), 3.13 (m, 1H, H19), 2.89 (d, 1H, *J* = 15.6 Hz, CH), 2.64 (m, 2H, H30), 2.48 (d, 1H, *J* = 15.6 Hz, CH), 0.80 – 2.40 (m, 23 H, CH, CH$_2$), 1.32 (m, 6H, 2 x OCH$_2$C*H*$_3$), 1.30 (s, 3H, CH$_3$), 1.28 (s, 3H, CH$_3$), 0.99 (s, 6H, CH$_3$), 0.90 (s, 3H, CH$_3$), 0.85 (s, 3H, CH$_3$), 0.81 (s, 3H, CH$_3$), 0.78 (m, 1H, H5); ^{13}C NMR ($CDCl_3$) δ (ppm): 182.8, 182.2, 170.6, 110.6, 81.4, 61.9, 61.8, 56.7, 50.6, 49.6, 42.4, 40.7, 40.5, 38.3, 37.9, 36.9, 33.9, 32.0, 30.9, 29.8, 28.5, 27.3, 26.9, 24.2, 23.6, 21.0, 19.7, 18.4, 17.4, 16.9, 16.6, 16.4, 14.5, 14.1; ^{31}P NMR ($CDCl_3$) δ (ppm): 27.9; HR-MS (APCI) *m/z*: $C_{40}H_{64}O_9P$ [(M–H)$^-$], Calc. 719.4288; Found 719.4276.

^{1}H NMR

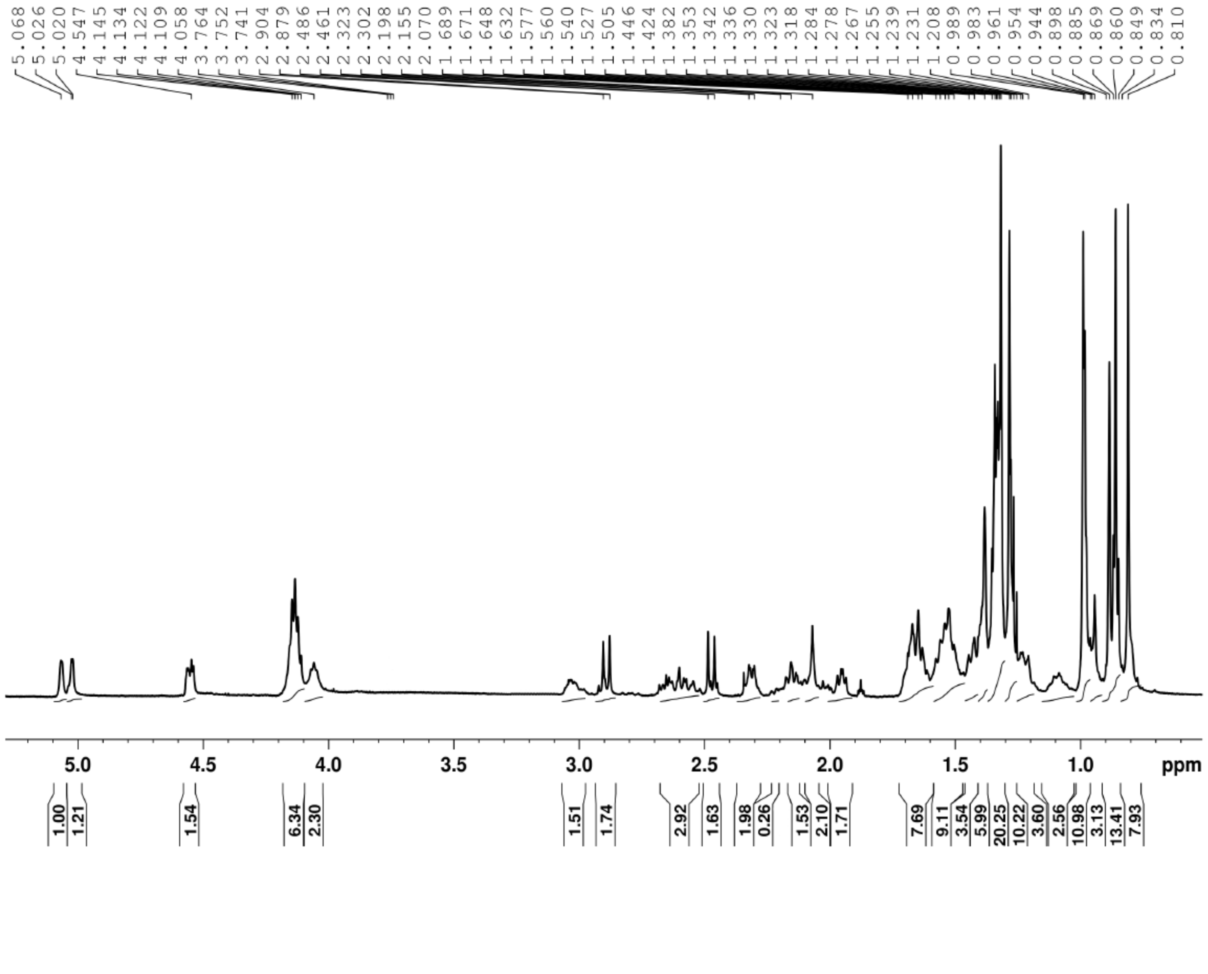

^{13}C NMR

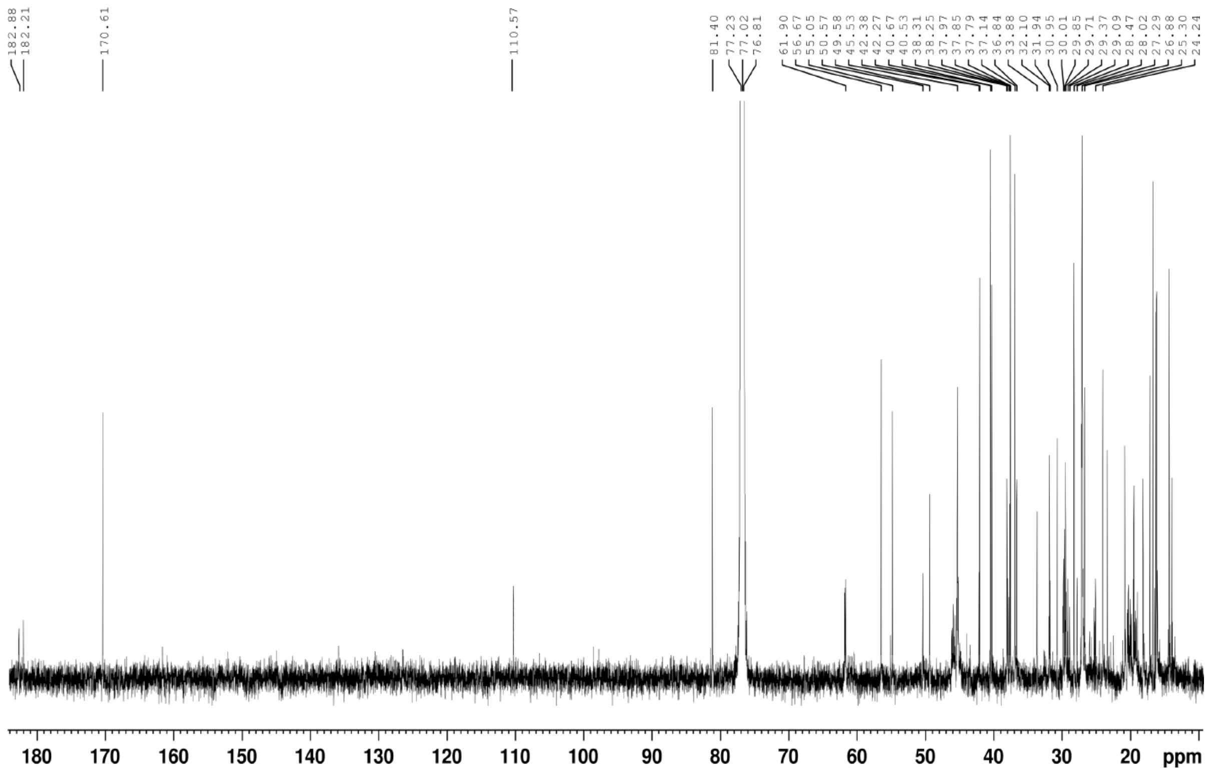

^{31}P NMR

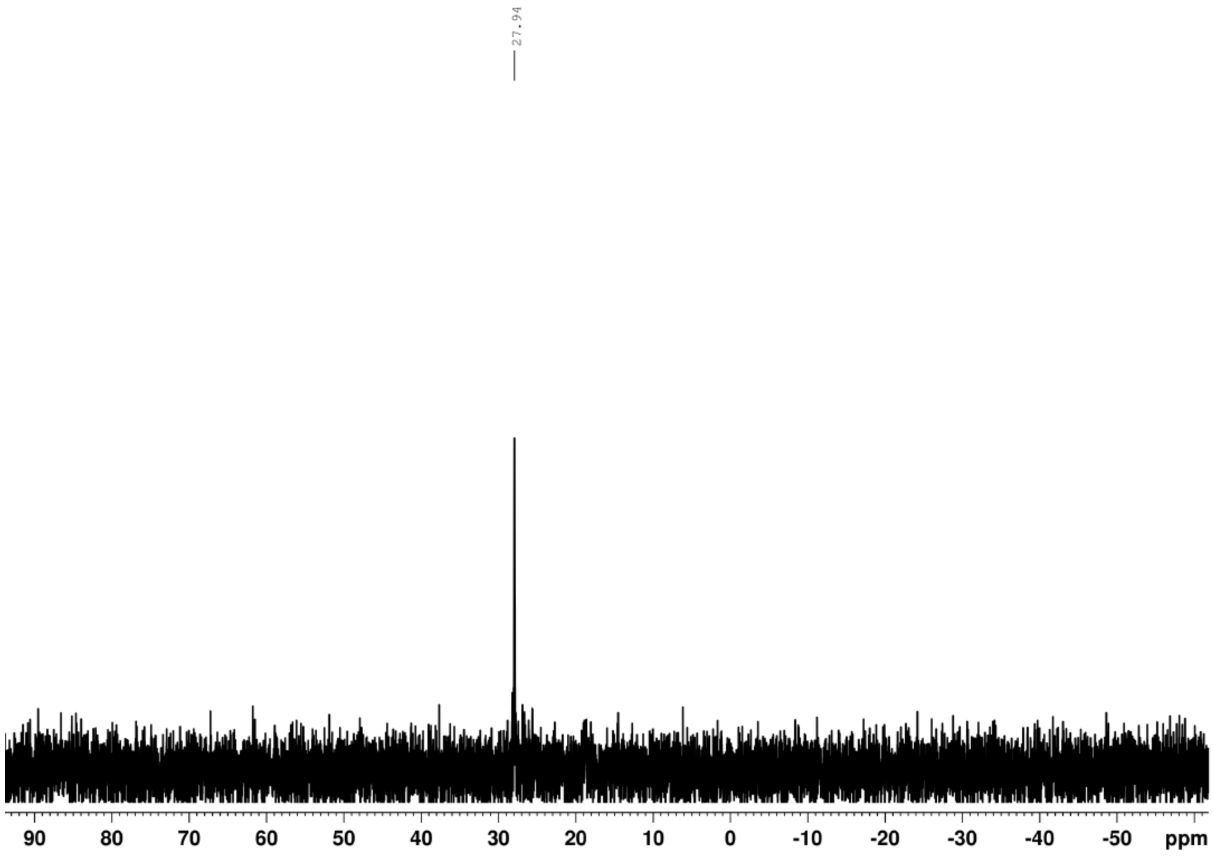

30-Diethoxyphosphoryl-3-O-(3’,3’-dimethylglutaryl)betulinic acid 14b

Yield 27%; mp 129-131 °C; R_f = 0.22 (chloroform/ethanol, 15:1, v/v); IR (KBr) ν (cm^{-1}): 3325, 2947, 1701, 1728, 1221, 1028, 789; ^{1}H NMR ($CDCl_3$) δ (ppm): 4.98 (m, 1H, H29), 4.93 (m, 1H, H29, 4.43 (m, 1H, H3), 4.05 (m, 4H, 2 x OC$H_2$$CH_3$), 2.95 (m, 1H, H19), 2.68 (d, 1H, *J* =13.2 Hz, H4’), 2.59 (m, 2H, H30), 2.43 (d, 1H, *J* =13.2 Hz, H4’), 2.31 (d, 1H, *J* =16.2 Hz, H2’), 2.17 (d, 1H, *J* =16.2 Hz, H2’), 1.27 (m, 6H, 2 x OCH_2CH_3), 1.10 – 2.30 (m, 29H, CH, CH_2), 1.10 (s, 3H, CH_3), 1.02 (s, 3H, CH_3), 0.90 (s, 3H, CH_3), 0.87 (s, 3H, CH_3), 0.79 (s, 3H, CH_3), 0.78 (s, 3H, CH_3), 0.73 (s, 3H, CH_3), 0.70 (m, 1H, H5); ^{13}C NMR ($CDCl_3$) δ (ppm): 182.1, 177.1, 172.1, 81.0, 68.3, 62.3, 58.6, 56.5, 55.1, 50.5, 49.8, 45.0, 44.1, 42.3, 40.6, 38.1, 38.0, 37.8, 37.2, 36.6, 34.0, 32.4, 34.1, 32.1, 31.0, 29.7, 28.8, 28.5, 28.3, 26.2, 23.9, 21.0, 18.3, 16.9, 16.8, 16.6, 16.3, 14.6; ^{31}P NMR ($CDCl_3$) δ (ppm): 28.0; HR-MS (APCI) *m/z*: $C_{41}H_{66}O_9P$ [$(M–H)^-$], Calc. 733.4445; Found 733.4435.

^{1}H NMR

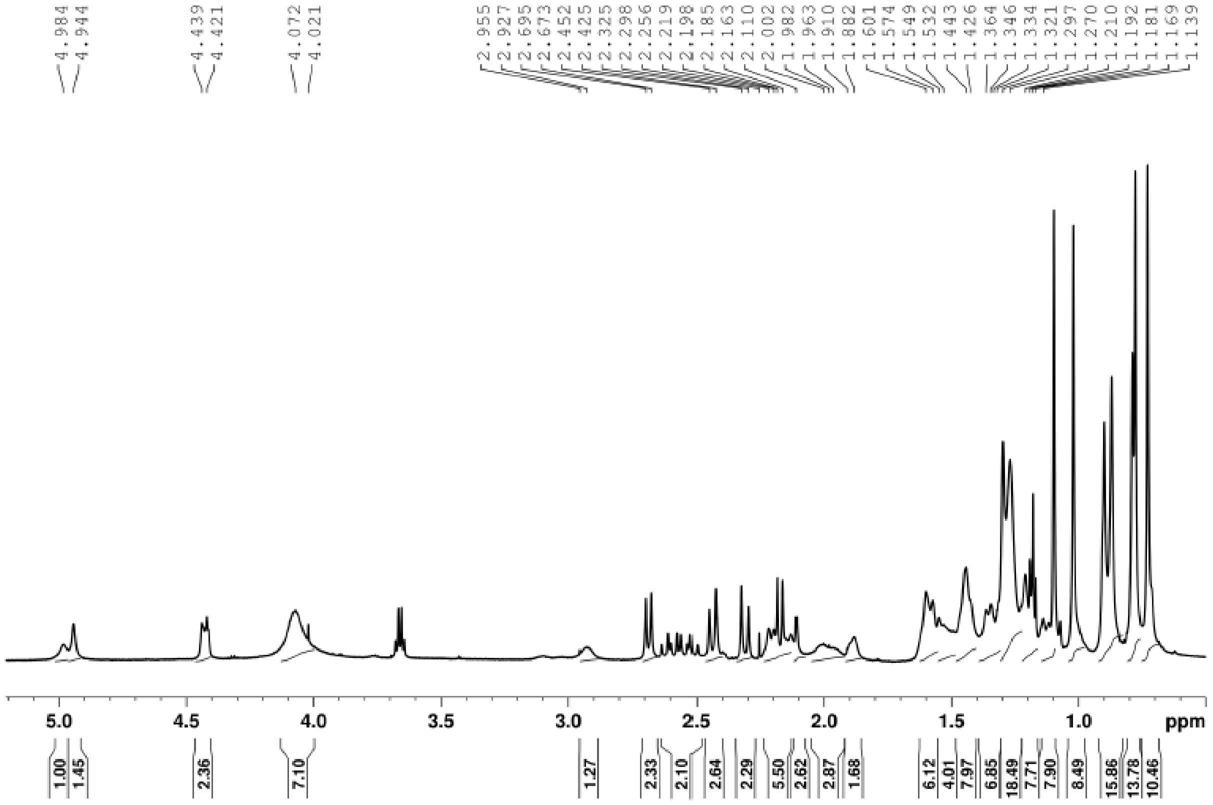

^{13}C NMR

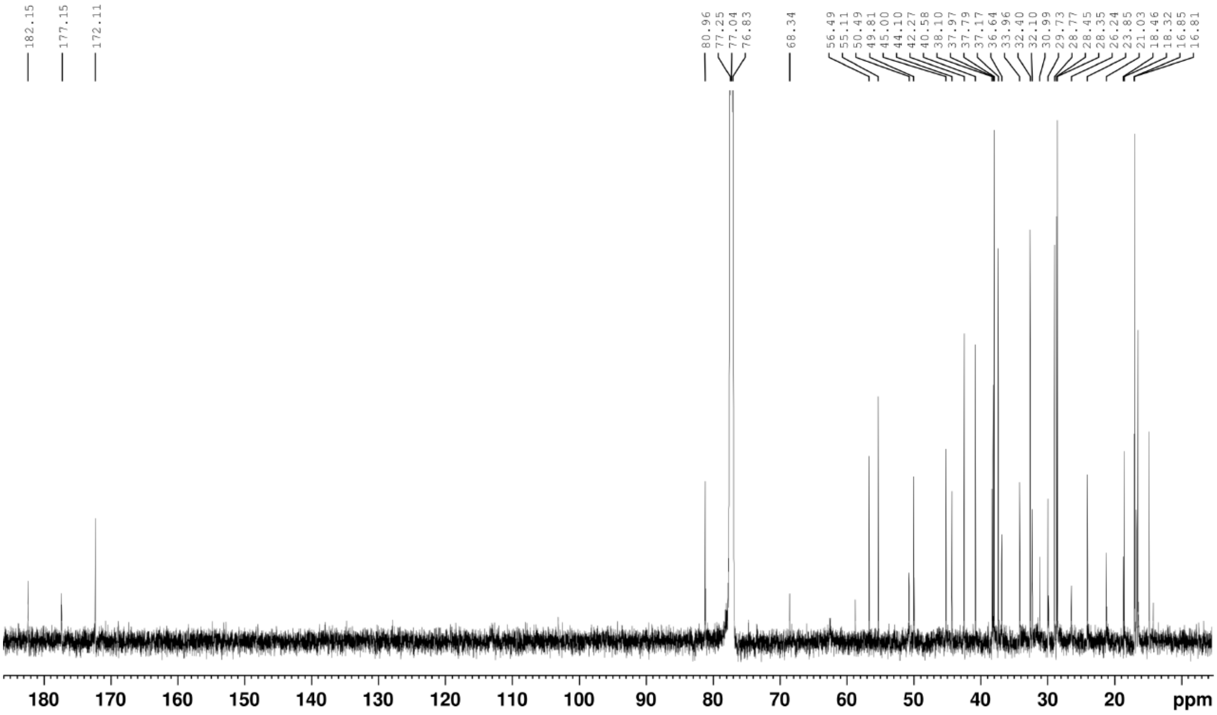

^{31}P NMR

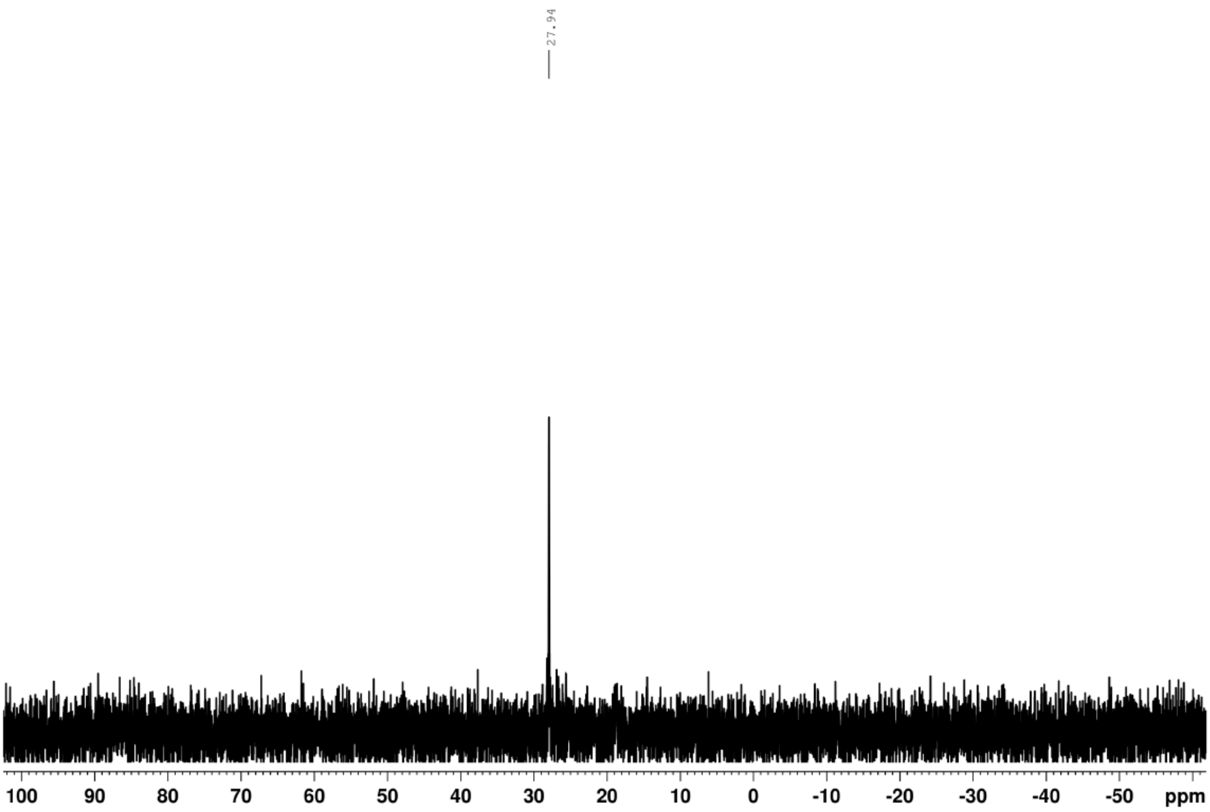

30-Diethoxyphosphoryl-3-O-(4',4'-dimethylglutaryl)betulinic acid 14c

Yield 26%; mp 119-124 °C; R_f = 0.28 (chloroform/ethanol, 15:1, v/v); IR (KBr) ν (cm^{-1}): 3543, 2945, 1705, 1219, 1026, 751; ^1H NMR ($CDCl_3$) δ (ppm): 4.98 (m, 1H, H29), 4.93 (m, 1H, H29), 4.90 (m, 1H, H3), 4.04 (m, 4H, 2 x OCH_2CH_3), 2.93 (m, 1H, H19), 2.5 (m, 2H, H30), 1.25 (m, 6H, 2 x OCH_2CH_3), 1.20 – 2.30 (m, 27H, CH, CH_2), 1.15 (s, 3H, CH_3), 1.13 (s, 3H, CH_3), 0.90 (s, 3H, CH_3), 0.83 (s, 3H, CH_3), 0.77 (s, 3H, CH_3), 0.75 (s, 3H, CH_3), 0.72 (s, 3H, CH_3), 0.75 (m, 1H, H5); ^{13}C NMR ($CDCl_3$) δ (ppm): 183.3, 182.2, 173.0, 80.8, 68.3, 62.0, 61.9, 58.51, 56.4, 55.2, 50.7, 50.0, 42.3, 41.6, 40.6, 38.3, 38.1, 37.9, 37.1, 36.6, 35.1, 34.0, 32.1, 31.0, 30.7, 29.7, 28.2, 26.3, 24.7, 24.3, 23.7, 21.0, 18.2, 16.8, 16.4, 16.1, 16.0, 14.7; ^{31}P NMR ($CDCl_3$) δ (ppm): 28.0; HR-MS (APCI) *m/z*: $C_{41}H_{66}O_9P$ [$(M–H)^-$], Calc. 733.4445; Found 733.4451.

^1H NMR

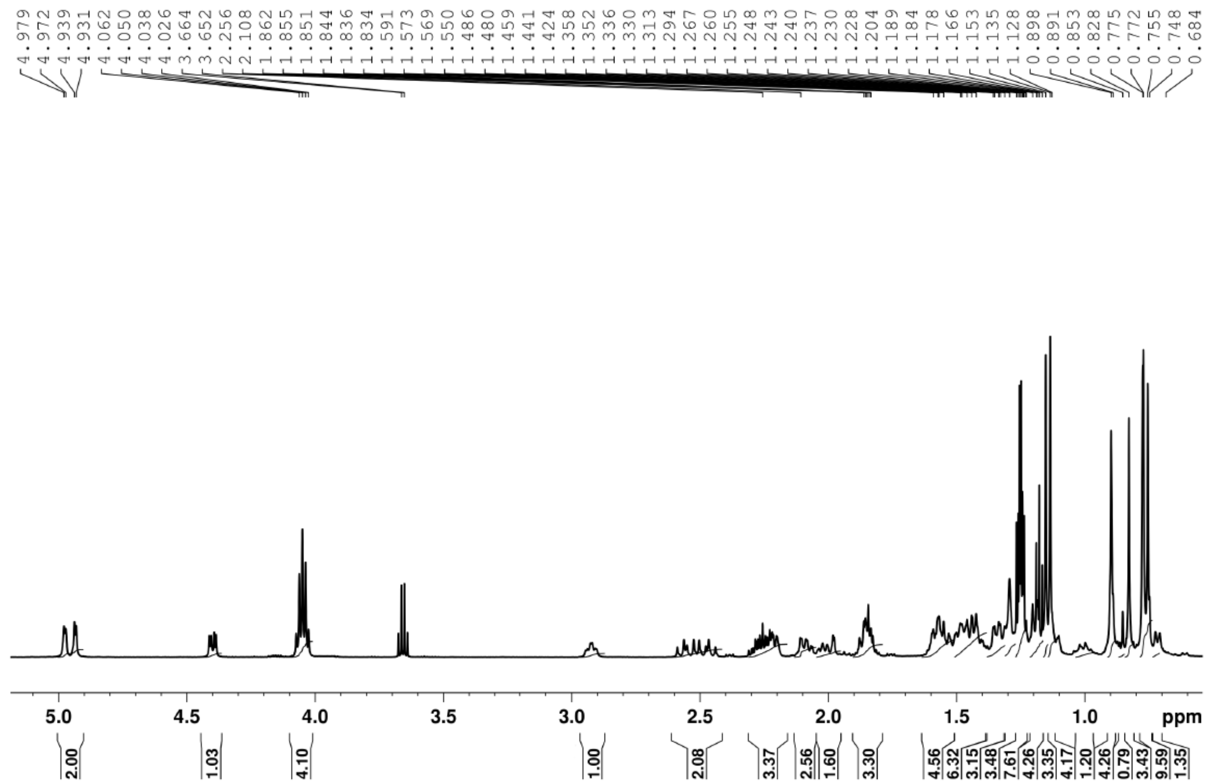

^{13}C NMR

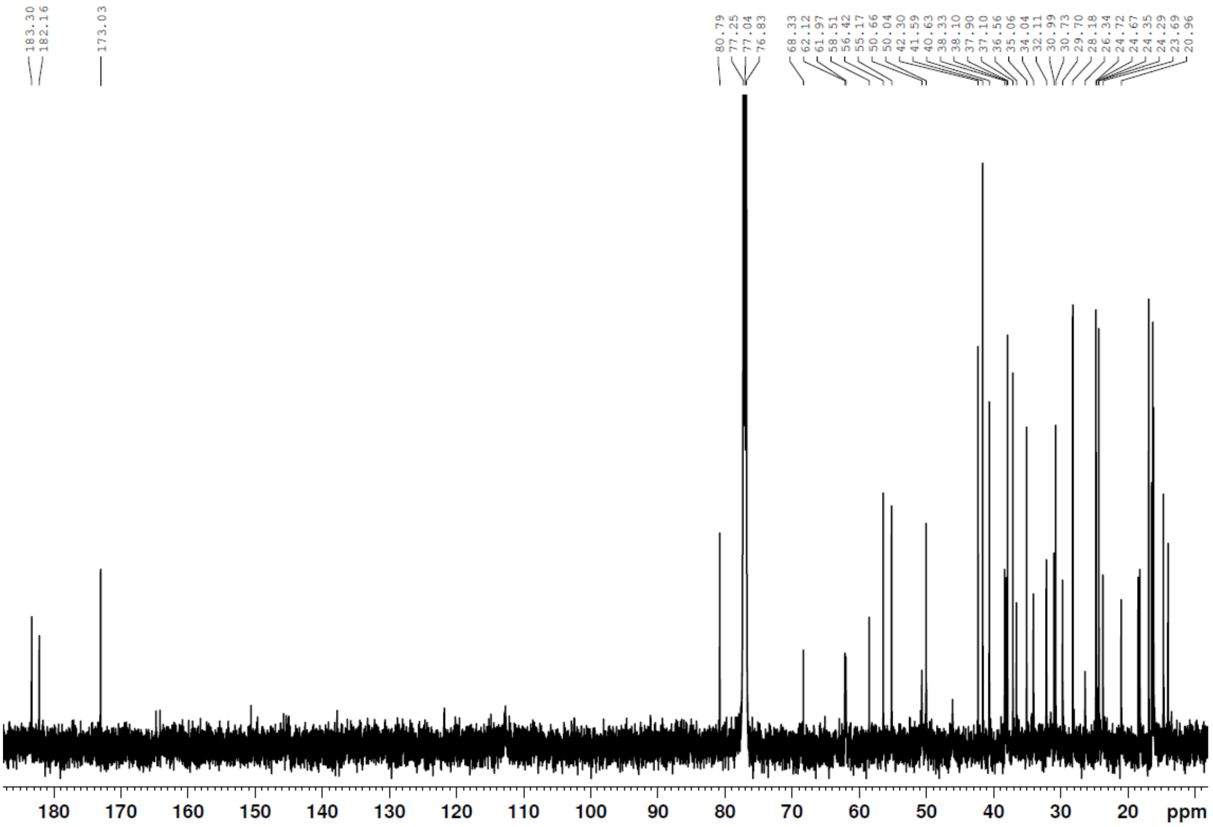

^{31}P NMR

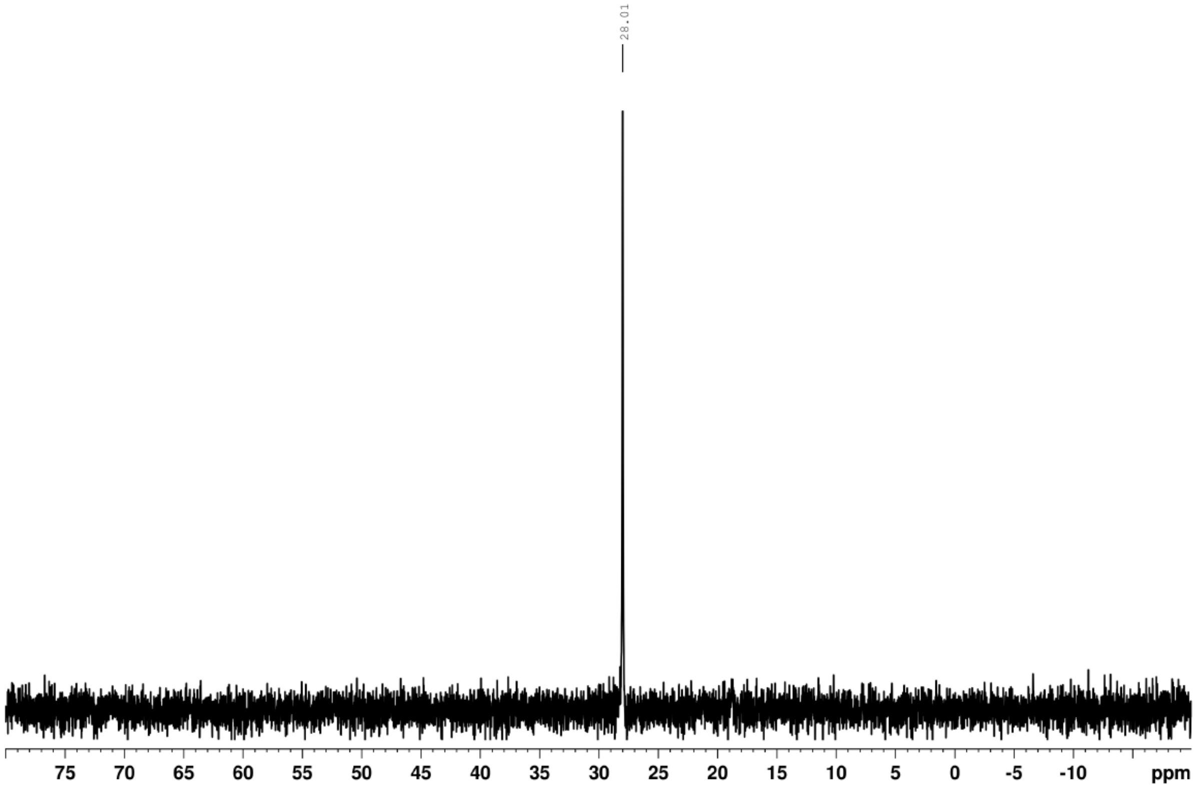

Charts of changes in cytotoxicity of compounds in the tested concentration range

Figure S1

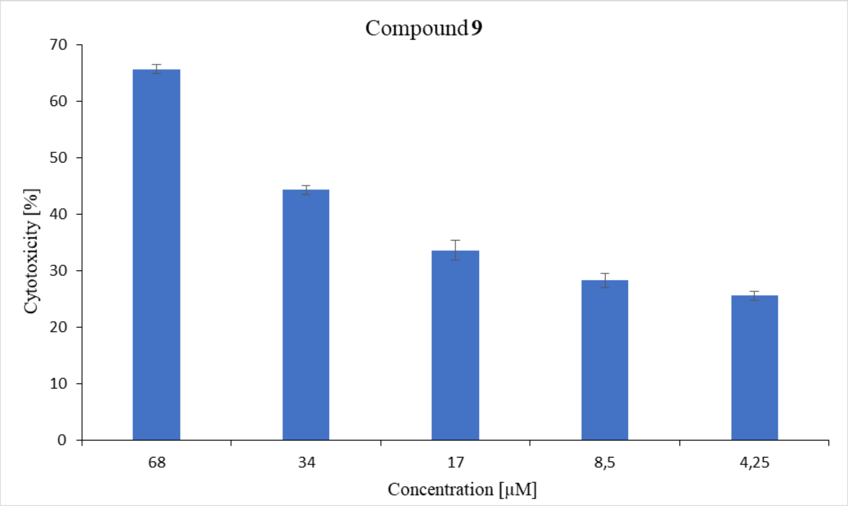

Figure S2

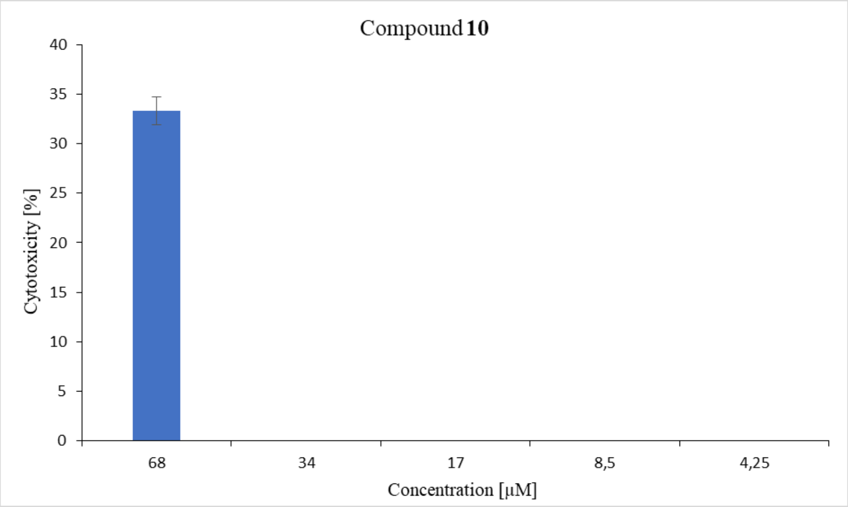

Figure S3

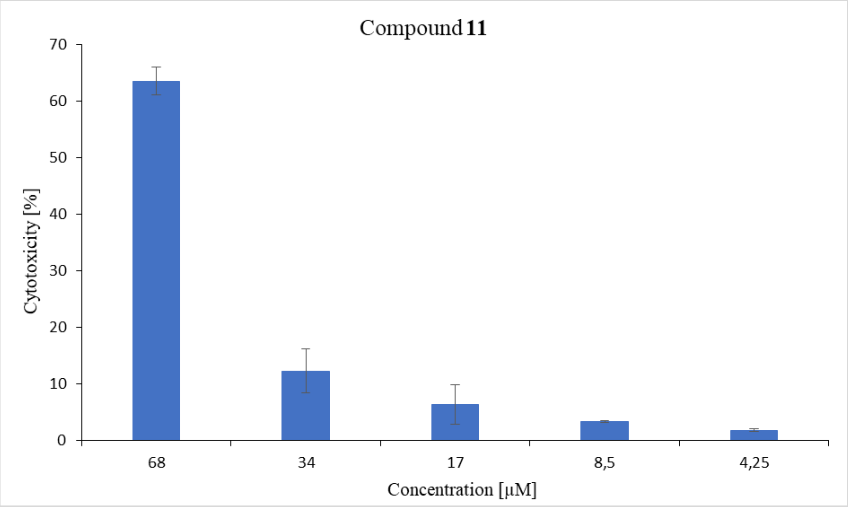

Figure S4

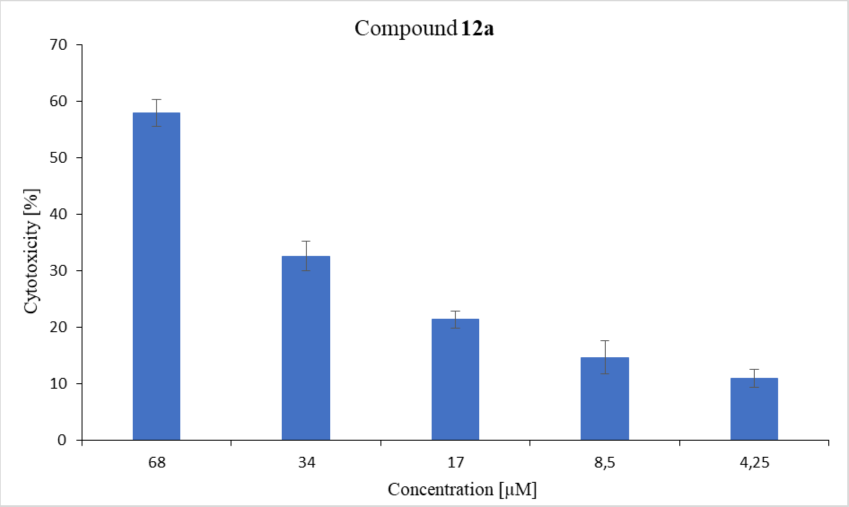

Figure S5

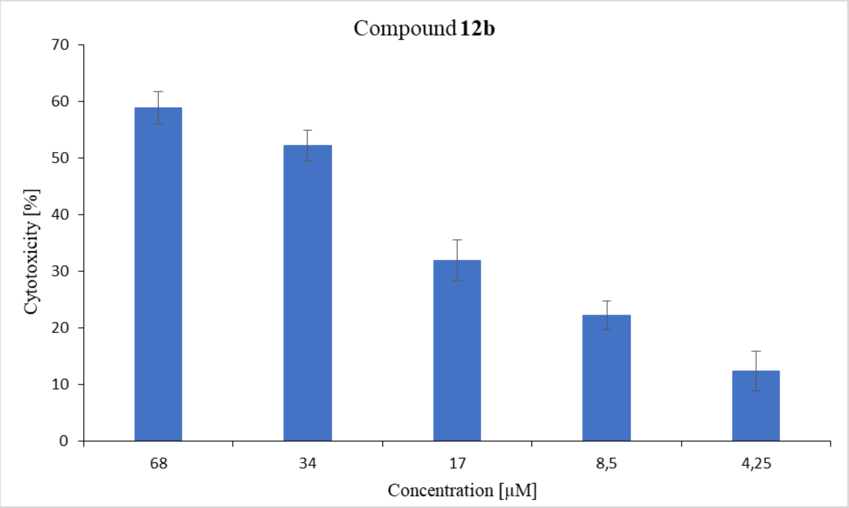

Figure S6

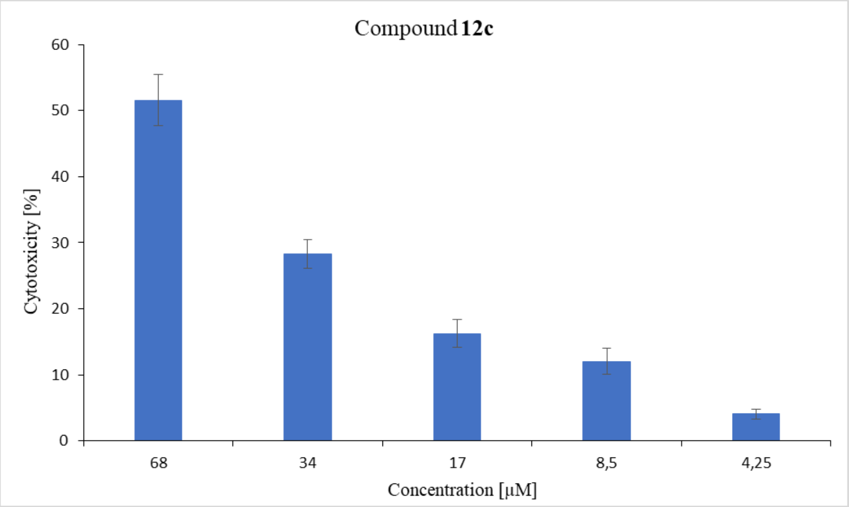

Figure S7

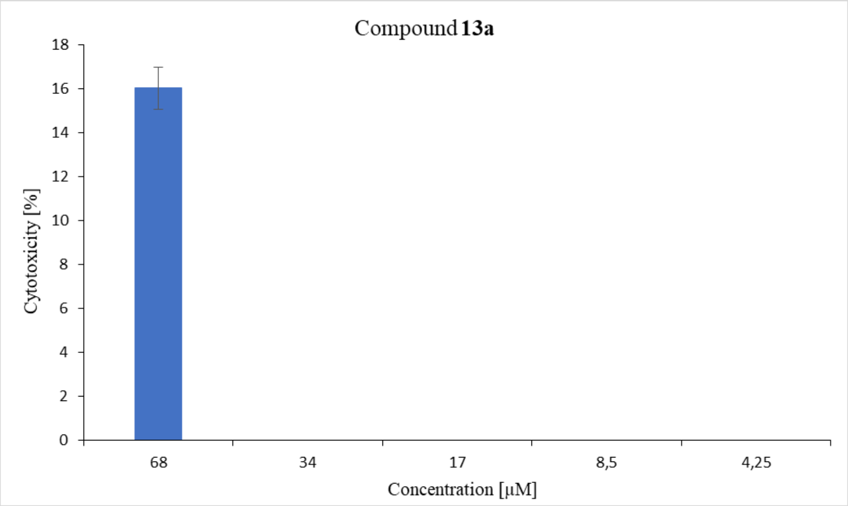

Figure S8

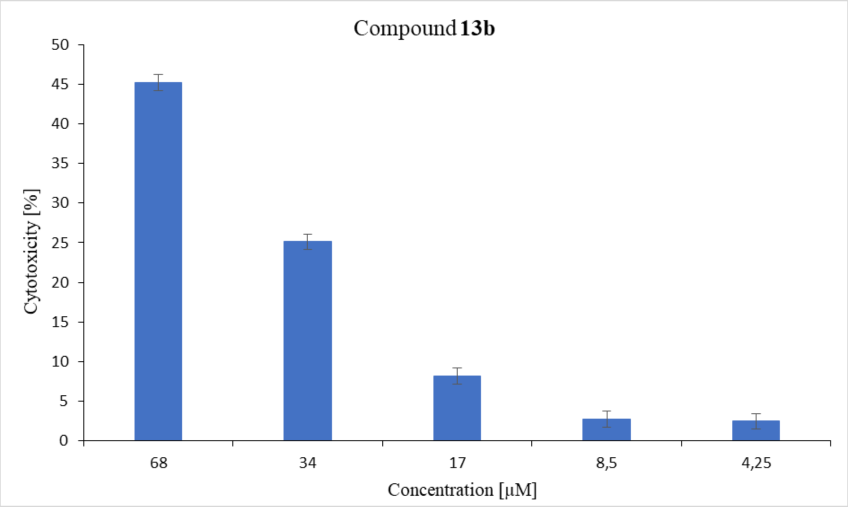

Figure S9

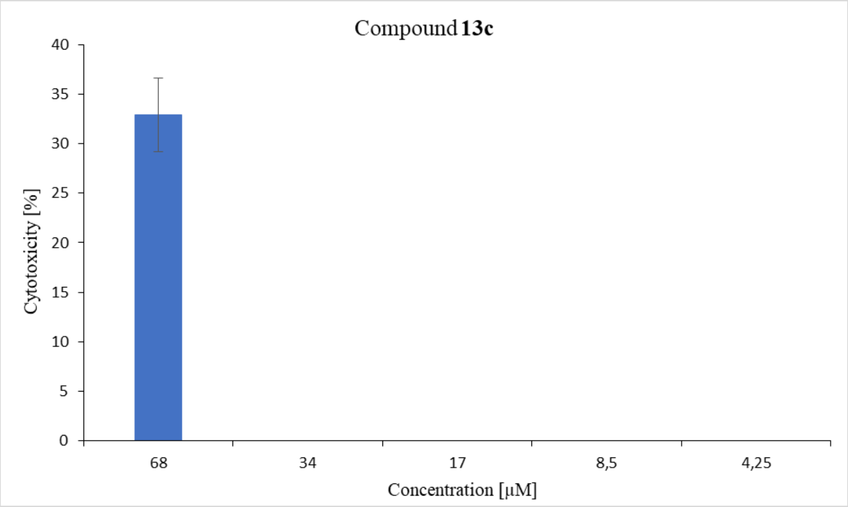

Figure S10

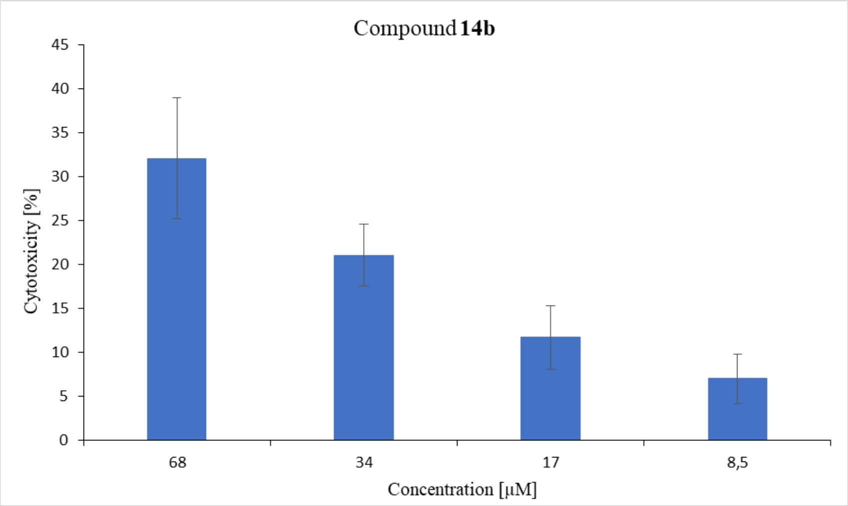

Figure S11

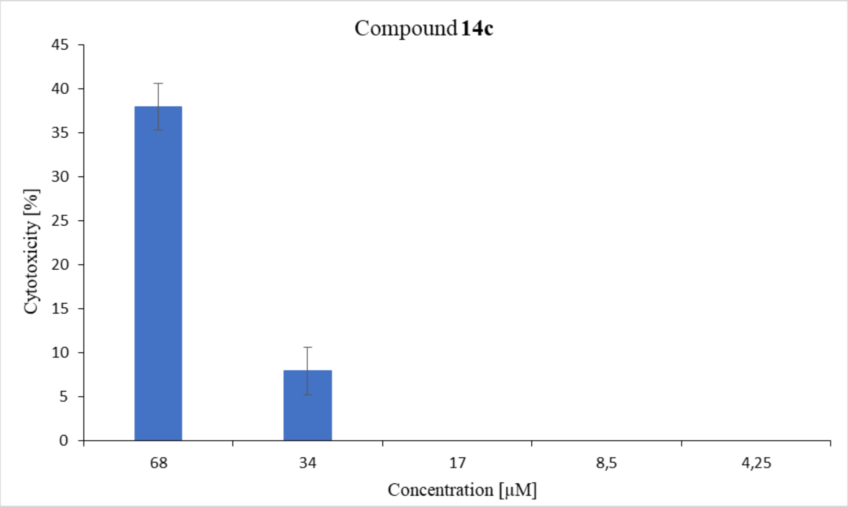

Figure S12

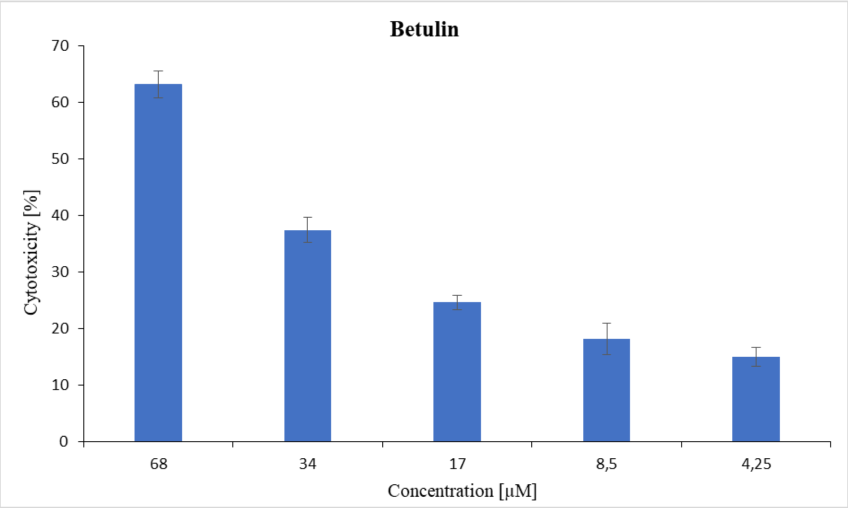

Figure S13

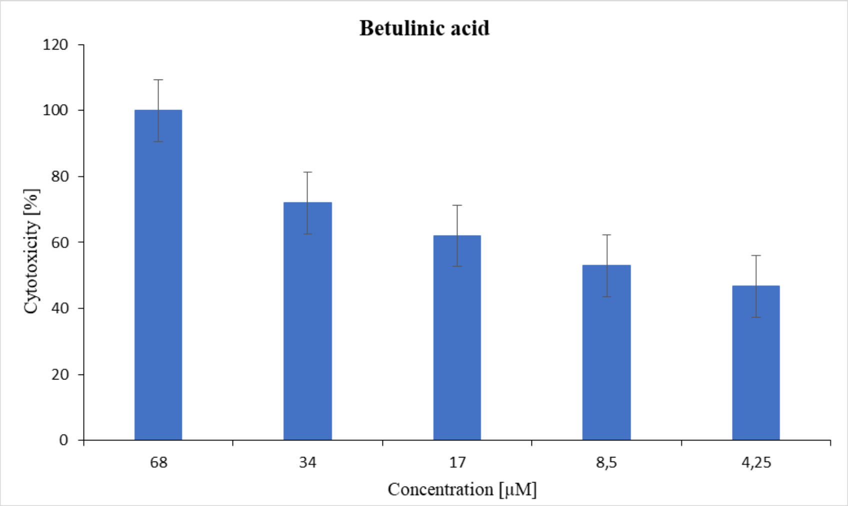

Betulinic acid
Cytotoxicity [%]
120
100
80
60
40
20
0
68
34
17
8,5
4,25
Concentration [µM]
